# MLN4924 suppresses tumor metabolism and growth of clear cell renal cell carcinoma by stabilizing nuclear FBP1

**DOI:** 10.1038/s41420-025-02426-8

**Published:** 2025-05-26

**Authors:** Yajing Yang, Yan Ma, Shiyin Fan, Jie Zhu, Bin Ye, Ruonan Zhang, Jiaxi Li, Hongchen Li, Zhencang Zheng, Yufeng Li, Lei Lv

**Affiliations:** 1 Ministry of Education Key Laboratory of Metabolism and Molecular Medicine, Department of Biochemistry and Molecular Biology, School of Basic Medical Sciences, Fudan University, Shanghai, 200032 China; 2 Clinical Laboratory, Taizhou Central Hospital (Taizhou University Hospital), Taizhou, 318000 Zhejiang China; 3 Department of paediatrics, Taizhou Central Hospital(Taizhou University Hospital), Taizhou, 318000 Zhejiang China; 4 Tongji Hospital, Shanghai Key Laboratory of Signaling and Disease Research, Frontier Science Center for Stem Cell Research, School of Life Sciences and Technology, Tongji University, Shanghai, 200092 China; 5 Department of Critical Care Medicine, Taizhou Central Hospital (Taizhou University Hospital), Taizhou, 318000 Zhejiang China; 6 Department of Pediatric Nephrology, Rheumatology and Immunology, Xinhua Hospital Affiliated to Shanghai Jiaotong University School of Medicine, Shanghai, 200092 China

**Keywords:** Drug development, Cancer metabolism, Mechanisms of disease

## Abstract

Fructose-1, 6-bisphosphatase (FBP1) is a tumor suppressor and frequently deficient in various cancers, including clear cell renal cell carcinoma (ccRCC). VHL inactivation mutations are usually observed in ccRCC, which can lead to abnormal activation of the HIF signaling pathway. FBP1 could enter the nucleus and restrain HIF function in a non-enzymatic manner. However, its regulatory mechanism in ccRCC tumorigenesis remains poorly understood. Here, we report that nuclear FBP1 is degraded through the ubiquitin-proteasome pathway, and CUL4B acts as Cullin-RING E3 ubiquitin ligase (CRL) to promote the degradation of FBP1 in nucleus, while the neddylation inhibitor MLN4924 could inactivate CUL4B E3 ligase, block proteasomal degradation of FBP1 and suppress HIF target gene expression, including GLUT1, LDHA, PDK1 and VEGF, leading to decreased glucose uptake and lactate and NADPH production, thereby repressing tumor growth of ccRCC. Furthermore, MLN4924 sensitizes ccRCC to γ-glutamylcysteine synthetase inhibitor Buthionine sulfoximine (BSO) treatment in vivo. Collectively, these findings proposed that MLN4924 could inhibit the tumor growth of VHL deficiency-driven ccRCC by stabilizing FBP1, providing new target and strategy for clinic treatment of ccRCC.

## Introduction

Clear cell renal cell carcinoma (ccRCC) is the most common pathological type of renal cell carcinoma (RCC), mainly occurring in elderly men over 60 years old [1]. Due to the clinical asymptomatic nature and relatively difficult to diagnose, nearly one-third of patients have metastatic spread at the time of diagnosis, and almost half of the patients die from the disease [2]. Emerging treatment modalities, such as cytokine treatment, targeted therapy, and immune checkpoint blockade, have improved the survival of ccRCC patients in certain extent, but these treatment strategies are still limited [3]. Thus, further exploration of the mechanisms underlying ccRCC pathogenesis has great clinical significance for managing patients.

Inactivation of the von Hippel-Lindau (VHL) tumor-suppressor gene accounts for ~90% of all ccRCC cases [4]. The VHL-HIF is a well-studied signaling pathway for the initiation and progression of ccRCC [5]. HIF overexpression modulates the metabolism of cancer cells and upregulates genes associated with metastasis. Fructose-1, 6-bisphosphatase (FBP1), a rate-limiting enzyme in gluconeogenesis, plays a key role in regulating glucose metabolism through facilitating gluconeogenesis and constraining glycolysis [6, 7]. FBP1 deficiency is associated with hypoglycemia and metabolic acidosis [8]. Apart from its canonical role in glucose metabolism, FBP1 has been reported to be downregulated in certain cancers and correlated with patient survival [9–11]. Current research believes that FBP1 inhibits tumors progression mainly through two distinct molecular mechanisms. On the one hand, the loss of FBP1 promotes tumor progression by enhancing glycolysis and accelerating cancer cells growth, thereby leading to a poor prognosis in patients with breast cancer, pancreatic cancer and liver cancer. On the other hand, recent study indicated that FBP1 could enter the nucleus and directly inhibit HIF function in a non-catalytic-activity-manner, thus restraining ccRCC progression [12]. Recently, a study showed that overexpressed FBP1 inhibited HIF-1α protein expression under hypoxic conditions, related to cell metabolism in breast cancer cells [13]. Likewise, FBP1 could antagonize the function of HIF1/2α in metabolic reprogramming to restrain ccRCC growth [14]. All these findings indicated that FBP1 may be closely associated with the VHL-HIF signaling pathway. Although the roles of FBP1 in multifarious cancers have been described, but how nuclear FBP1 protein is regulated by posttranslational modifications remains poorly understood in ccRCC.

Ubiquitination is a common type of post-translational modification, which plays a crucial role in regulating protein turnover and participates in the diverse signaling pathways associated with tumor initiation and progression [15]. Cullin-RING E3 ligases (CRLs) are the largest class of E3 ubiquitin ligases in the ubiquitin-proteasome system, which transfers ubiquitin to substrates and regulates the cellular protein degradation [16]. A typical CRLs is a polyprotein complex containing four components: Cullin family protein (cullins 1, 2, 3, 4A, 4B, 5, 7, and 9), RING protein (RBX1 and RBX2/SAG), adaptor protein and substrate recognition subunit [17]. Neddylation is a kind of ubiquitination-like post-translational modification, in which NEDD8, a ubiquitin-like peptide, is transferred to the substrate protein to regulate its biological activation. Cullin family protein is considered to be the substrate of neddylation, and cullin neddylation is a necessary requirement for CRL function. MLN4924, a highly selective small molecule inhibitor of NEDD8 activating enzyme (NAE) [18], leads to the inactivation of CRLs. MLN4924 has demonstrated effective anti-cancer activity in preclinical studies as a single agent or in combination with chemotherapy drugs [19]. Additionally, MLN4924 has been widely utilized in preclinical studies involving in cell culture, xenograft models, and transgenic mouse models [20].

Reduced glutathione (GSH) is a tripeptide with antioxidant properties that protects cell membranes from the detrimental effects of reactive oxygen species (ROS) generated during cell metabolism [21]. The first stage of GSH synthesis involves the interaction of cysteine and glutamate, catalyzed by γ-glutamylcysteine synthetase (γGCS) to form γ-glutamylcysteine, which is a crucial step in GSH production. Buthionine sulfoximine (BSO), as an inhibitor of GSH synthesis by inhibiting γ-GCS, has been widely used in pharmacological and biomedical studies. Its clinical potential in cancer therapy was increasingly recognized, as it exhibits less toxicity than its precursor, methionine sulfoximine (MSO), and may be safely administered via intravenous injection in humans [22].

This study aimed to provide a comprehensive understanding of how MLN4924 enhances the protein levels of FBP1 in the nucleus and its implications in VHL-deficient ccRCC progression. In our investigation, we observed that CUL4B binds to FBP1, promoting its ubiquitin-mediated proteasomal degradation in ccRCC. Additionally, we found that lactate production, glucose uptake, and NADPH generation decreased when CUL4B was knocked out and ccRCC cells were treated with MLN4924. Importantly, the combined treatment of MLN4924 and BSO effectively suppressed ccRCC tumor growth in vivo. Our study revealed a previously unknown regulatory pathway of FBP1 in ccRCC, providing a new strategy for clinical interventions of ccRCC patients.

## Results

### Nuclear FBP1 is degraded through the ubiquitin-proteasome pathway

**Fig. 1:**
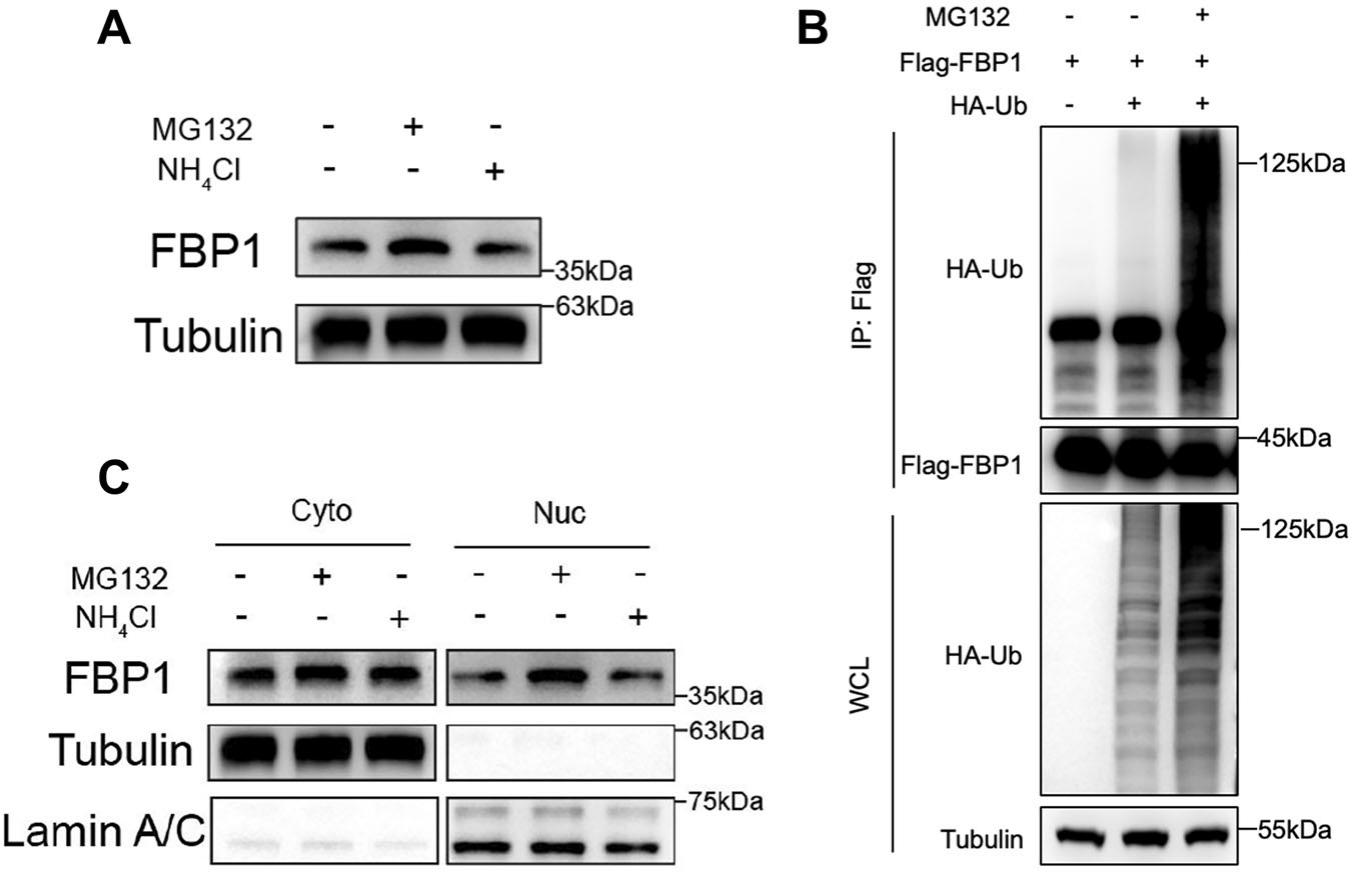
Nuclear FBP1 is degraded through the ubiquitin-proteasome pathway. **A** Western blot was performed to analyze FBP1 expression with the treatment of DMSO, MG132 (10 μM), or NH_4_Cl (10 mM) in 786-O cells. **B** Immunoprecipitation and Western blot with the indicated antibodies were performed to determine the level of FBP1 ubiquitylation in HEK293T cells. **C** Western blot was performed to assess the cytoplasmic and nuclear expression of FBP1 after the treatment of DMSO, MG132 (10 μM) or NH_4_Cl (10 mM) in 786-O cells.

The role of FBP1 in tumor metabolism and growth is independent of its enzymatic activity and instead is mediated by FBP1 entering nucleus, where it inhibits the function of HIF in ccRCC [23]. However, the specific mechanisms by which posttranslational modifications regulate nuclear FBP1 protein remain poorly understood. To elucidate these mechanisms, we first treated 786-O cells with the proteasome inhibitor MG132 and the autophagy-lysosome inhibitor NH_4_Cl separately. We found that only the presence of MG132 led to a significant increase of FBP1 protein levels (Fig. 1A). Then, we induced ectopic expression of Flag-tagged FBP1 and HA-tagged Ub in HEK293T cells and treated them with MG132 and DMSO, respectively. We observed a significant increase in ubiquitination levels of FBP1 upon treatment with MG132 (Fig. 1B). Interestingly, when we performed nuclear-cytoplasmic fractionation of 786-O cells, we observed a remarkable increase of FBP1 protein in the nucleus after treatment with MG132, while FBP1 protein levels showed no significant change in the cytoplasm (Fig. 1C). These observations indicate that FBP1 protein in the nucleus may undergo degradation through the ubiquitin-proteasome pathway.

### MLN4924 upregulates the protein levels of FBP1 in nucleus

**Fig. 2:**
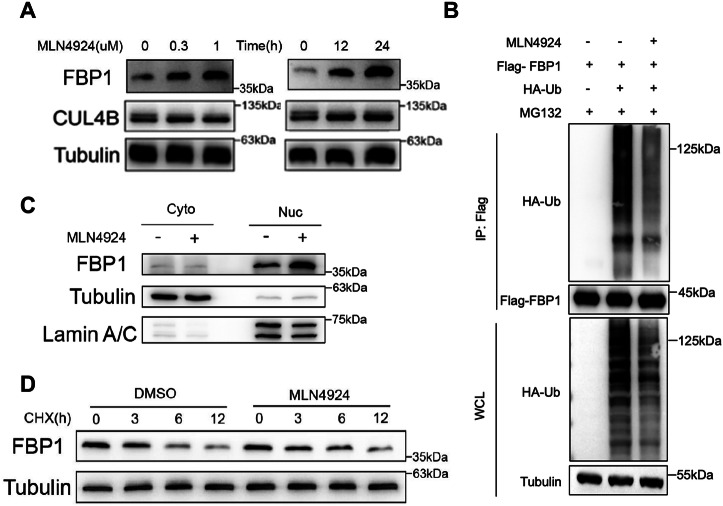
MLN4924 upregulates the protein levels of FBP1 within the nucleus. **A** 786-O cells were treated with MLN4924 at the indicated concentrations or times. The protein expression levels of FBP1 were measured by Western blot. **B** Immunoprecipitation and Western blot with the indicated antibodies were performed to determine the level of FBP1 ubiquitylation with or without MLN4924 in HEK293T cells. **C** Western blot was performed to assess the cytoplasmic and nuclear expression of FBP1 after the treatment of DMSO or MLN4924 (1 μM, 24 h) in 786-O cells. **D** 786-O cells were pretreated with DMSO or MLN4924 (1 μM) for 12 h, and then treated with 100 μg/mL cycloheximide (CHX). Cell proteins were harvested at the indicated times for analysis.

Since MLN4924 can inhibit the neddylation of Cullin-RING E3 ubiquitin ligases (CRLs) by suppressing NEDD8, thereby causing the inactivation of CRLs and the accumulation of associated substrates [18]. Hence, we sought to explore whether CRLs are involved in the degradation process of nuclear FBP1. Herein, we found that MLN4924 significantly increased FBP1 protein levels in 786-O cells, in addition, elevated FBP1 levels were observed by the treatment of MLN4924 in both time-dependent and dose-dependent manners (Fig. 2A). Moreover, MLN4924 could largely rescue the ubiquitination level of FBP1 in the presence of MG132 (Fig. 2B). Similar to the observations with MG132 treatment, we found that MLN4924 resulted in dramatically enhanced FBP1 accumulation in the nucleus (Fig. 2C). Meanwhile, we tested whether MLN4924 regulates the stability of FBP1. The cycloheximide (CHX) chase experiment revealed that the half-life of the FBP1 protein was noticeably extended by the treatment of MLN4924 in 786-O cells (Fig. 2D). Taken together, these results strongly indicate that MLN4924 could enhance the protein levels of FBP1 within the nucleus.

### CUL4B degrades FBP1 protein in nucleus

**Fig. 3:**
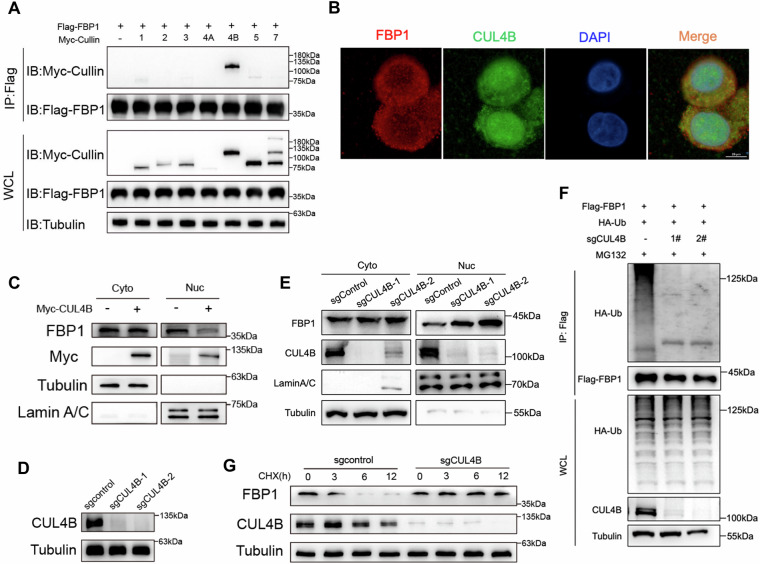
CUL4B degrades the FBP1 protein in the nucleus. **A** Screening the Cullin-RING E3 ubiquitin ligase of FBP1 by transfecting Flag-FBP1 and Myc-Cullins as indicated. Immunoprecipitation and Western blot analysis confirmed the interaction between FBP1 and CUL4B. **B** Co-localization of FBP1 and CUL4B in 786-O cells was detected by immunofluorescence. **C** Western blot was performed to assess the cytoplastic and nuclear expression of FBP1 after transfection with CUL4B plasmids. **D** Expression of CUL4B was identified in wild-type (WT) 786-O cells and two distinct CUL4B knockout 786-O cell lines by Western blot. **E** Western blot was performed to assess the cytoplasmic and nuclear expression of FBP1 after CUL4B knockout. **F** Immunoprecipitation and Western blot with the indicated antibodies were performed to determine the level of FBP1 ubiquitylation in WT 786-O cells and two distinct CUL4B knockout cell lines. **G** sgControl and sgCUL4B 786-O cells were incubated with 100 μg/mL CHX at the indicated time points for Western blot analysis.

Previous results showed that CRLs are involved in the degradation process of nuclear FBP1, as evidenced by the ability of MLN4924 to increase the levels of nuclear FBP1 protein. To find out which CRLs is responsible for the degradation process of nuclear FBP1, we induced overexpression of Myc-Cullins (Myc-CUL1, Myc-CUL2, Myc-CUL3, Myc-CUL4A, Myc-CUL4B, Myc-CUL5, Myc-CUL7) in HEK293T cells and determined combination or not with FBP1 protein by Co-IP experiments. We found that only CUL4B exhibited a physical interaction with FBP1 (Fig. 3A). Coincidently, CUL4B is also localized in the nucleus [24]. We performed confocal microscopy to assess the colocalization of CUL4B and FBP1. Interestingly, results revealed that CUL4B and FBP1 colocalize in both the nucleus and cytoplasm (Fig. 3B). We next generated overexpression of Myc-CUL4B in 786-O cells and observed that CUL4B mainly resulted in a significant decrease in FBP1 protein levels within the nucleus, (Fig. 3C). Conversely, increased FBP1 in the nucleus was found in 786-O cells when CUL4B was knocked out by CRISPR-Cas9 technology (Fig. 3D, E). The ubiquitination level was also significantly reduced upon CULB4 knockout (Fig. 3F). Furthermore, similar to MLN4924 treatment, CUL4B was knocked out significantly prolonged the half-life of FBP1 (Fig. 3G). These results reveal that CUL4B binds to FBP1 and facilitates the ubiquitin-mediated proteasomal degradation of FBP1.

### MLN4924-CUL4B axis inhibits HIF signaling and tumor metabolism

**Fig. 4:**
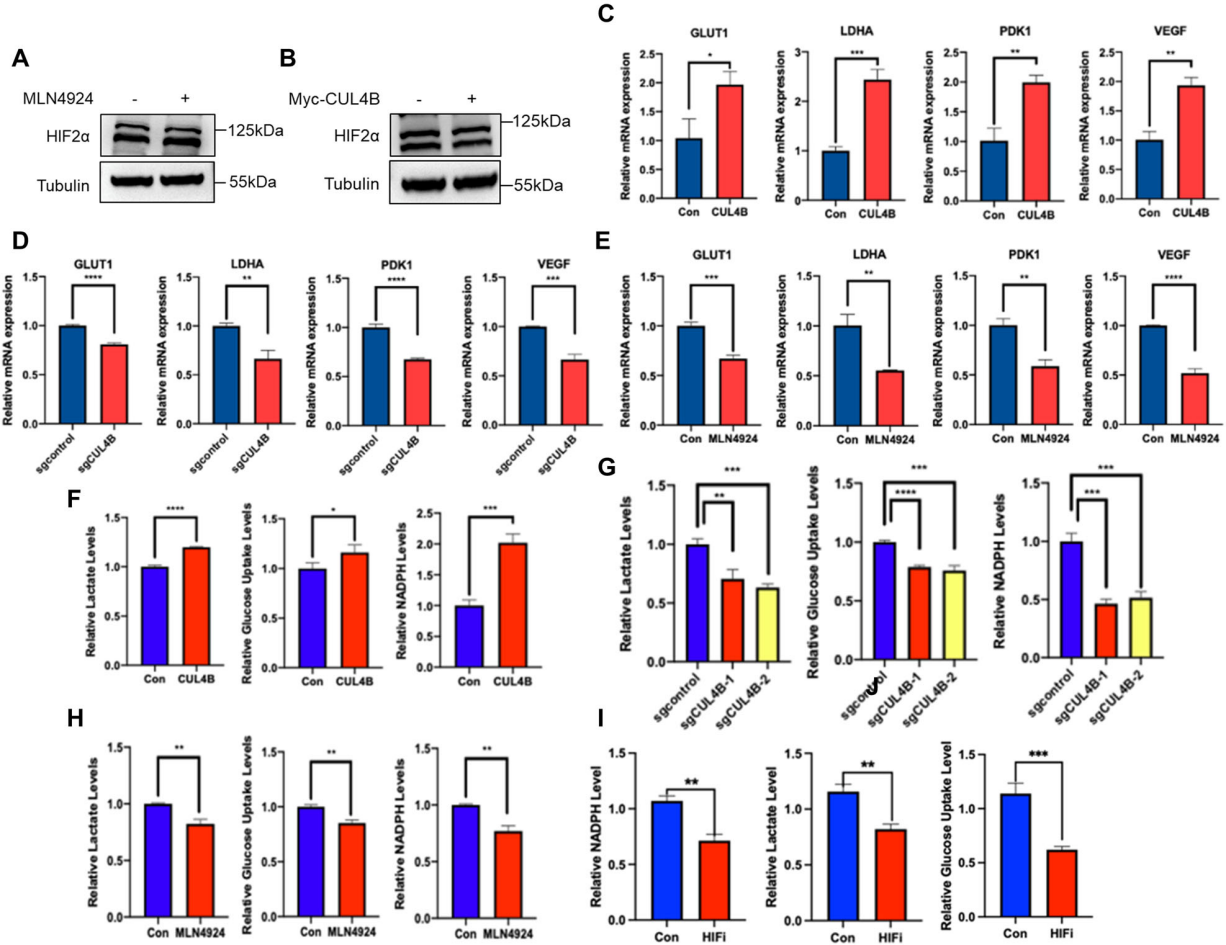
MLN4924 inhibits downstream effectors of HIF and correlates with tumor metabolism. **A** Western blot was performed to assess the protein expression levels of HIF-2α after treated with MLN4924 (1 μM). **B** Western blot was performed to assess the protein expression levels of HIF-2α after transfection with CUL4B plasmids. **C** GLUT1, LDHA, PDK1 and VEGF mRNA levels were quantified by qPCR after overexpressed of CUL4B in 786-O cells. **D** WT and CUL4B knockout 786-O cells were used to measure GLUT1, LDHA, PDK1 and VEGF mRNA levels. **E** GLUT1, LDHA, PDK1 and VEGF mRNA levels were quantified by qPCR with or without MLN4924 treatment in 786-O cells. **F** Lactate, glucose uptake and NADPH levels were measured after overexpressed of CUL4B in 786-O cells. **G** WT and CUL4B knockout 786-O cells were used to measure lactate, glucose uptake and NADPH levels. **H** Lactate, glucose uptake and NADPH levels were measured with or without MLN4924 treatment in 786-O cells. **I** Lactate, glucose uptake and NADPH levels were measured with or without HIF-2α inhibitor treatment in 786-O cells. HIFi, HIF-2α inhibitor. *P < 0.05, **P < 0.01, ***P < 0.001, ****P < 0.001.

As mentioned above, FBP1 can translocate into the nucleus to inhibit the function of HIF in VHL-deficient ccRCC, while FBP1 is degraded by CUL4B via ubiquitin-mediated proteasomal degradation. Interestingly, we observed no significant changes in HIF2α protein levels in 786-O cells treated with MLN4924 or overexpressing CUL4B (Fig. 4A, B). This led us to hypothesize that the MLN4924-CUL4B axis might influence the HIF signaling by regulating the expression of HIF downstream target genes. HIF promotes the transcription of glucose transporter type 1 (GLUT1), pyruvate dehydrogenase kinase 1 (PDK1), and vascular endothelial growth factor (VEGF) genes, which are associated with glycolysis, pentose phosphate pathway and angiogenesis [12]. Based on these, we further examined the contribution of CUL4B to the downstream gene of HIF. The upregulation of GLUT1, LDHA, PDK1, and VEGF expression was observed in CUL4B overexpression 786-O cells (Fig. 4C). Conversely, the expression of GLUT1, LDHA, PDK1, and VEGF was suppressed when CUL4B was knocked out in 786-O cells (Fig. 4D). Similarly, the expression of GLUT1, LDHA, PDK1, and VEGF was suppressed in the presence of MLN4924 treatment (Fig. 4E). We next investigated whether CUL4B and MLN4924 affect the tumor metabolism in ccRCC. Our results indicated that overexpression of CUL4B led to an increase in lactate production, glucose uptake, and NADPH generation, and there was a opposite results occurred when CUL4B was knocked out in 786-O cells (Fig. 4F, G). Similarly, lactate production, glucose uptake, and NADPH generation were reduced after MLN4924 and HIF-2α inhibitor treatment (Fig. 4H, I). Collectively, these findings support the critical role of CUL4B and MLN4924 in tumor metabolism.

### The combination of MLN4924 and BSO effectively suppresses tumor growth of ccRCC in vivo

**Fig. 5:**
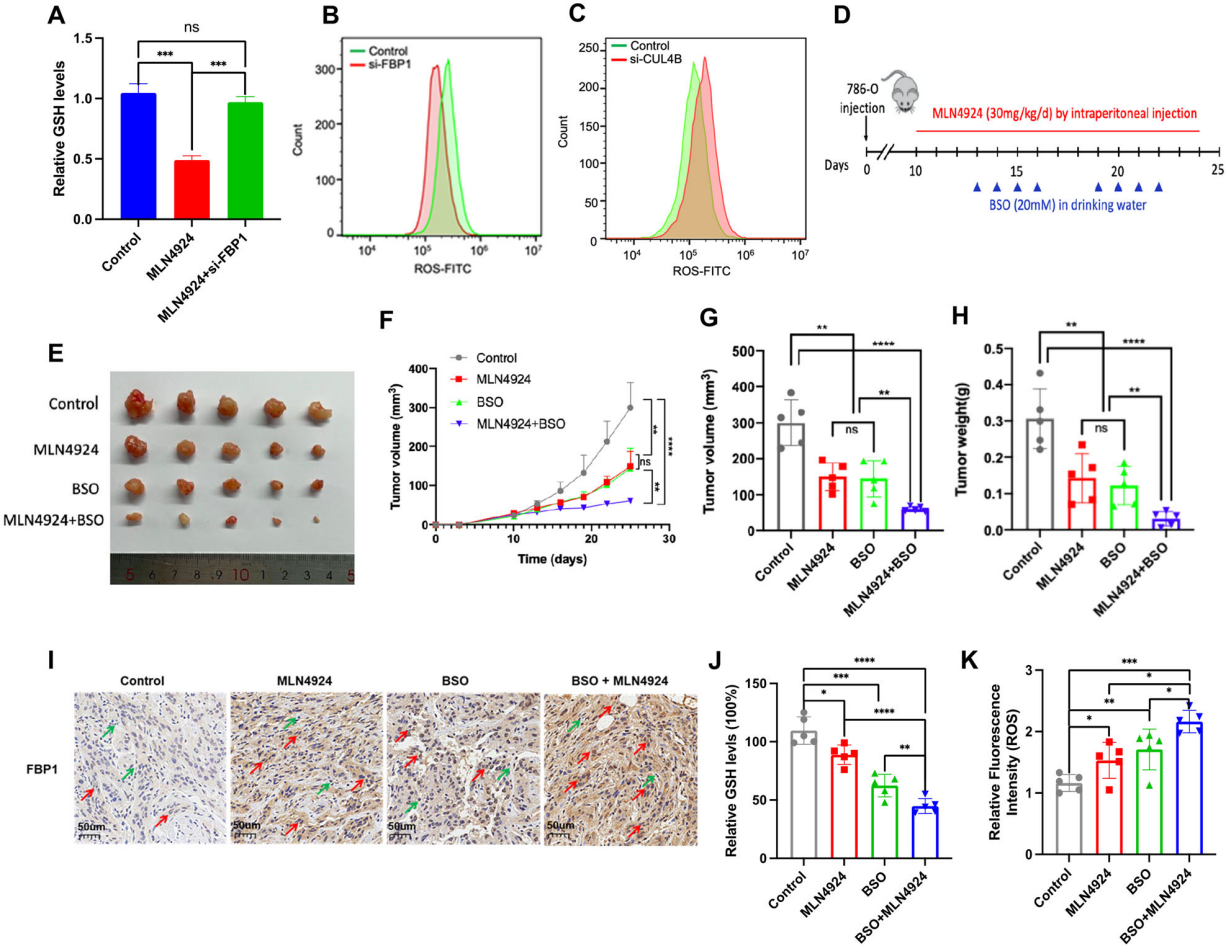
The combination of MLN4924 and BSO effectively suppresses tumor growth in vivo. **A** GSH levels were measured in different groups in 786-O cells. **B** ROS levels were measured in 786-O cells knocked down with FBP1 and in the control group. **C** ROS levels were measured in 786-O cells knocked down with CUL4B and in the control group. **D** Schematic of the xenograft experiments. 786-O cells were subcutaneously injected into athymic BALB/c nude mice and treated as indicated (n = 5). **E** Tumors were harvested at the end of experiment and photographed. **F** The tumor growth was measured and plotted. The tumor volume (**G**) and weight (**H**) were calculated. **I** Representative images of FBP1 IHC staining in tumor specimens from different mouse groups. **J** GSH levels were measured in tumor specimens from different mouse groups. **K** ROS levels were measured in tumor specimens from different mouse groups. *P < 0.05, **P < 0.01, ***P < 0.001, ****P < 0.001.

L-Buthionine-sulfoximine (BSO) has been shown to effectively inhibit γ-glutamylcysteine synthetase (γ-GCS), which blocks the synthesis of glutathione (GSH) [25]. As mentioned above, we observed a decrease in NADPH generation in 786-O cells following treatment with MLN4924. Previous studies have shown that GSH reductase (GR) can reduce oxidized GSH (GSSG) back to GSH in the presence of NADPH [26]. We observed that the treatment of MLN4924 led to a reduction in the GSH levels in 786-O cells, and this downregulation effect could be reversed by the knockdown of FBP1 (Fig. 5A). Additionally, we noted that the knockdown of FBP1 resulted in decreased levels of ROS in the cells, whereas the knockdown of CULB4 led to an increase in cellular ROS levels (Fig. 5B, C). Therefore, we hypothesize that treatment with MLN4924 may inhibit GSSG reduction to GSH in vivo by suppressing NADPH. To this end, we established mouse tumor models using 786-O cells to assess the efficacy of MLN4924 and BSO treatment (Fig. 5D). Results suggested that treatment with MLN4924 or BSO alone led to a moderate inhibition of tumor growth in mice (Fig. 5E–H). Notably, the combination of MLN4924 and BSO markedly suppressed tumor growth and represented the most effective treatment (Fig. 5E–H). Immunohistochemistry of tumor specimens revealed a significant increase in FBP1 expression in both the MLN4924 and MLN4924 + BSO treatment groups (Fig. 5I). Additionally, GSH level assessments revealed that MLN4924 treatment significantly reduced GSH levels, with the BSO alone and MLN4924 + BSO combination groups showing even lower GSH levels (Fig. 5J). In contrast, MLN4924 treatment significantly increased ROS levels, with the highest levels observed in the MLN4924 + BSO combination groups (Fig. 5K). In conclusion, these results indicated that the combination of MLN4924 and BSO could effectively suppress ccRCC tumor growth in vivo.

## Discussion

**Fig. 6:**
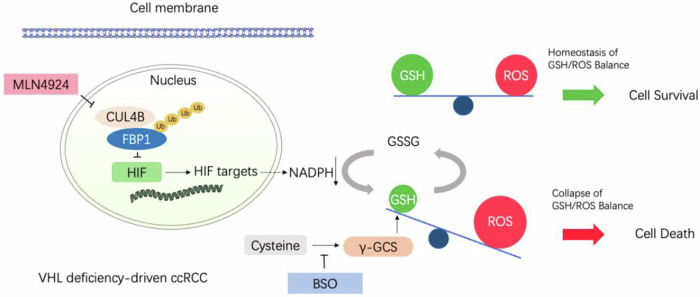
A schematic model showing the nuclear regulation mechanism of FBP1 in ccRCC.

In this work, we revealed the posttranslational regulation of nuclear FBP1 in ccRCC, as depicted in the schematic diagram (Fig. 6). Evidence shows that the overexpression of FBP1 inhibits cell proliferation and significantly reduces tumor growth in various cancers [10, 14, 27]. FBP1 translocates into the nucleus and non-enzymatically suppresses the HIF function, thereby suppressing HIF target genes in VHL-deficient ccRCC. Our research has discovered that CUL4B, acting as an E3 ubiquitin ligase, promotes the degradation of nuclear FBP1 via the ubiquitin-proteasome pathway. MLN4924 inhibits CRLs by blocking NEDD8, inhibiting CUL4B-mediated degradation of FBP1 and increasing the protein levels of nuclear FBP1. Non-enzymatically, FBP1 inhibits HIF, leading to the suppression of downstream target genes including GLUT1, LDHA, PDK1, and VEGF. Furthermore, MLN4924 has been shown to inhibit lactate production, glucose uptake, and NADPH generation in ccRCC. Our findings further shed light on the mechanisms by which FBP1 reprograms tumor metabolism through the HIF pathway, thereby inhibiting the occurrence and development of ccRCC.

In addition, our study also highlights that the combination of MLN4924 and BSO exerts enhanced cytotoxicity against tumors in vivo. The hypothesized mechanism is as follows: GSH, the major endogenous non-protein antioxidant, plays a vital role in efficient scavenging of excess ROS and endogenous/exogenous electrophiles to protect cells. The balance between ROS and GSH is critical for maintaining normal cell functions, and an imbalance could cause oxidative stress and cell death. NADPH is a necessary substrate for the reduction of oxidized GSSG to GSH, and our study revealed that MLN4924 can lead to a reduction in NADPH production, subsequently resulting in reduced levels of reductive GSH. Additionally, BSO can inhibit the synthesis of GSH by blocking γ-GCS, which is essential for GSH production. When GSH is suppressed by MLN4924 and BSO through separate pathways, the levels of GSH are significantly reduced, leading to increase ROS generation and inhibition of ccRCC tumor growth. This provides new insights into the clinical precision treatment of ccRCC.

VHL mutations occur at the early stage of ccRCC oncogenesis [28], but the loss of VHL function alone is not sufficient for the development of ccRCC in humans and mice [29]. This indicates the involvement of further regulatory mechanisms in the formation of ccRCC. Studies have demonstrated that FBP1 expression is positively correlated with favorable prognosis in ccRCC patients [14], and FBP1 was also regulated by ubiquitination in liver and pancreatic cancer [30]. Actually, FBP1 is commonly downregulated in VHL-deficient ccRCC [31]. In this context, the role of FBP1 in tumor metabolism and proliferation is independent of its enzymatic activity, instead, it enters the nucleus and inhibits the function of HIF, thereby suppressing cell proliferation, glycolysis, and the pentose phosphate pathway through non-catalytic activity [32]. Understanding the regulatory mechanisms of nuclear FBP1 could provide a theoretical basis for combination targeted therapies and aid in the clinical identification of potential drugs and prognostic markers in ccRCC. In terms of clinical applications, MLN4924 has demonstrated promising anticancer activity in numerous preclinical studies, evaluating its efficacy as a monotherapy or in combination with chemotherapy agents [33]. BSO has been shown to effectively inhibit γ-GCS and block the synthesis of GSH [34]. The combination therapy of MLN4924 and BSO represents an efficient approach to the regulation of oxidative stress, showcasing tremendous therapeutic potential.

In summary, we revealed a novel regulation of nuclear FBP1 in ccRCC cells and demonstrated that the combination of MLN4924 and BSO exhibits superior tumor-killing effects. This study contributes to the understanding of the relationship between metabolic characteristics and anticancer therapeutic efficacy in ccRCC, providing new insights into cancer biology and clinical treatment of ccRCC.

## Materials and methods

### Cell culture, transfection and reagents

786-O and HEK293T were cultured in DMEM medium (Meilun Biotechnology) supplemented with 10% FBS and 1% penicillin–streptomycin solution. All cell lines were grown at 37 °C with 5% CO_2_ humidified atmosphere, tested negative for mycoplasma contamination. Transfection of the plasmid was performed using the EZ-Trans (Life-iLab). MLN4924 (Topscience), MG132 (Topscience), NH_4_CL (Sangon Biotech), and CHX (Topscience) were used for in vitro studies following the manufacturer’s instruction.

### Plasmids

An expression plasmid coding for human Flag-tagged FBP1 was cloned into corresponding vector. CUL1, CUL2, CUL3, CUL4A, CUL4B, CUL5, CUL7 were cloned into Myc-tagged vector. Clone ubiquitin (Ub) into HA-tagged destination vector. The establishment of gene knockout (KO) cell lines by using CRISPR-Cas9 technology, sgRNAs sequences for human CUL4B (target sequences: 5’-CACCGCATATGTTTCAGGGATTCAT-3’) were subcloned into the pLenti-CRISPR v2 vector. All constructed plasmids were confirmed by sanger sequencing.

### Co-immunoprecipitation, western blot and CHX-chase analysis

Cell lysates were prepared for co-immunoprecipitation and western blot analysis using standard protocols. The antibodies we used were as follows: FBP1 (SAB1405798, 1:1000, sigma), Flag tag (HOA012FL01, 1:5000, AbHO), Lamin A/C (P02545, 1:2000, Biogot technology), Tubulin (66031-1-Ig, 1:5000, Proteintech), CUL4B (12916-1-AP, 1:1000, Proteintech), HA tag (HOA012HA01, 1:5000, AbHO). To confirm the half-life of FBP1, cells were intervened with 100 ug/mL CHX for the indicated time followed.

### Ubiquitination assay

To detect FBP1 ubiquitination, co-transfection with control or expressing Flag-FBP1 and HA-Ub vector plasmid, 48 h after transfection, cells were treated with 10 μM MG132 for 10 h. Then Cells were harvested and lysed in NP-40 lysis buffer containing protease inhibitors and 1% SDS on ice, boiled for 10 min, after that, cell lysates were diluted 10-fold by adding NP-40 lysate without SDS component. the supernatant was subjected to immunoprecipitation. The ubiquitination assay was performed by immunoblotting.

### RT‑qPCR

Total RNA was extracted from 786-O cells using EZ-press RNA Purification Kit (EZ Bioscience), reverse-transcribed to cDNA by using 4×Reverse Transcription Master Mix (EZ Bioscience), then cDNA was used to the qPCR through the 2× SYBR qPCR Mix (KTSM, AlpaLife). Actin was set as the internal control; all qPCR primers were synthesized in BioSune biotechnology (Shanghai). The sequences were as follows: GLUT1-F: GGCCAAGAGTGTGCTAAAGAA, GLUT1-R: ACAGCGTTGATGCCAGACAG; LDHA-F: TTGACCTACGTGGCTTGGAAG, LDHA-R: GGTAACGGAATCGGGCTGAAT; PDK1-F: CTGTGATACGGATCAGAAACCG, PDK1-R: TCCACCAAACAATAAAGAGTGCT, VEGF-F: AGGGCAGAATCATCACGAAGT, VEGF-R: AGGGTCTCGATTGGATGGCA, Actin-F: GGCATAGAGGTCTTTACGGATGTC, Actin-R: TATTGGCAACGAGCGGTTCC.

### Detection of tumor metabolism

Lactic acid production was assayed using a lactic acid kit (Nanjing jiancheng). According to the manufacturer’s protocols, culture medium containing the cell samples to be tested was mixed with lactate assay buffer, incubated at 37 °C for 10 min, and then aliquoted into a 96-well plate (150 µL per well). The lactate levels were measured at an absorbance of 530 nm using a microplate reader.

Glucose uptake level was assayed using a Glucose Uptake-GloTM Assay kit (Promega). According to the manufacturer’s protocols, cells were seeded onto 6-well plate, the culture medium was carefully aspirated, and 1 mL 1 mM 2-deoxyglucose was added. The solution was mixed thoroughly by gentle shaking and incubated at room temperature for 10 min. Subsequently, 500 µL of Stop Buffer was added, and 75 µL of the sample was transferred to a white opaque 96-well plate. To each well, 25 µL of Neutralization Buffer was added, followed by thorough mixing. Then, 100 µL of 2DG6P Detection Reagent was added, and the mixture was gently shaken until uniform. After incubation at room temperature for 2–3 h, the fluorescence intensity was measured using a microplate reader with an integration time of 0.3–1 s.

NADP+/NADPH level was assessed using NADP+/NADPH test kit (Beyotime Biotechnology). According to the manufacturer’s protocols, cells were seeded onto 6-well plate, the culture medium was carefully aspirated, and 1 mL PBS was added. Cells were harvested using a cell scraper and transferred into a 1.5 mL microcentrifuge tube. After centrifugation, the supernatant was discarded, and 200 µL of NADP+/NADPH extraction buffer was added. The sample was centrifuged for 10 min, and the supernatant was collected. The supernatant was incubated in a 60 °C metal bath for 30 min, followed by centrifugation for 5 min. Subsequently, 50 µL of the supernatant was transferred to a 96-well plate and incubated at 37 °C in the dark for 10 min. To each well, 10 µL of the color development reagent was added, mixed thoroughly, and further incubated at 37 °C in the dark for 10–20 minutes. The absorbance at 450 nm was measured using a microplate reader.

### ROS assay

ROS levels were measured according to the manufacturer’s instructions. Briefly, cells were incubated in a 6-well plates containing fluorescent dye (Invitrogen, 88-5930-74) at 37 °C for 30 min. After incubation, cells were washed twice with PBS and harvested by trypsinization. Cells were then analyzed using flow cytometry, and the FITC channel signals were plotted as shown in the figure.

### GSH assay

The relative GSH concentration was measured using a Glutathione Assay Kit (Beyotime, S0053). According to the manufacturer’s protocols, the measurement of GSH was performed using a kinetic analysis. The catalytic activity of GSH (nmol/L) continuously reduces 5,5’-dithiobis-(2-nitrobenzoic acid) (DTNB) to 5-thio-2-nitrobenzoic acid (TNB). The generated oxidized GSSG is recycled by glutathione reductase (GSR) and NADPH. Within a range of up to 2 mmol/L, the reaction rate is proportional to the glutathione concentration. The yellow product (TNB) was quantified spectrophotometrically at 412 nm.

### Immunofluorescence and Immunohistochemistry

Cells were washed once with PBS and fixed with 4% paraformaldehyde at room temperature for 10 min. Fixed cells were permeabilized with 0.1% Triton X-100, followed by two washes with PBS. The coverslips were then blocked for 1 hour in 0.1% BSA and incubated overnight at 4 °C with the primary antibody. Subsequently, the coverslips were washed twice and incubated with the secondary antibody at room temperature for 1 h. Imaging was performed using the Zeiss LSM 510 Meta confocal microscopy system.

Mouse tumor tissue slides were deparaffinized, rehydrated through an alcohol gradient, and subjected to antigen retrieval using sodium citrate buffer. The sections were blocked with 5% normal goat serum, 0.1% Triton X-100, and 3% H_2_O_2_ in PBS at room temperature for 60 min, followed by overnight incubation with the appropriate primary antibody at 4 °C. Immunohistochemical staining was performed using DAB to detect HRP-conjugated antibodies. Nuclei were counterstained with Hoechst.

### Animal experiments

6–8 weeks-old Male C57BL/6 nude mice were ordered from JieSiJie Laboratory Animals. Briefly, a total of 1 × 10^6^ 786-O cells were subcutaneously injected into the flanks of C57BL/6 nude mice. The mice were randomly divided four groups when most of the mice had developed solid tumors (about 10 days). For MLN4924 treatment, mice were treated s.c. with 30 mg/kg/day for 14 days. For BSO treatment, mice were treated p.o. with 20 mM for 8 days via drinking water. Mice were sacrificed when tumors exceeded 1500 mm^3^ or became ulcerated. Tumor size was monitored by electronic caliper every 3 days and calculated by the formula: 1/2 × width^2^ × length. All these studies were evaluated and approved by the Ethics Committee of the Basic Medical School at Fudan University.

### Statistical analysis

GraphPad Prism v.8.0 software was employed in carrying out statistical analyses. All of quantitative data were presented as mean ± standard deviation (SD), Comparisons between two groups for difference analysis were performed with two-tailed Student’s *t* test. All experiments were performed with at least three biological replicates. Significance level was indicated as *, *p* < 0.05; **, *p* < 0.01; ***, *p* < 0.001; ****, *p* < 0.0001 and *p* < 0.05 was considered statistically significant.

## Supplementary information

Original western blots

## Data Availability

The authors confirm that the data supporting the findings of this study are available within the article.
