# Supplementary figures and images for: MLN4924 suppresses tumor metabolism and growth of clear cell renal cell carcinoma by stabilizing nuclear FBP1
Source: Cell Death Discov. 2025 May 26;11:253. doi: 10.1038/s41420-025-02426-8 (PMC12106737; doi:10.1038/s41420-025-02426-8)

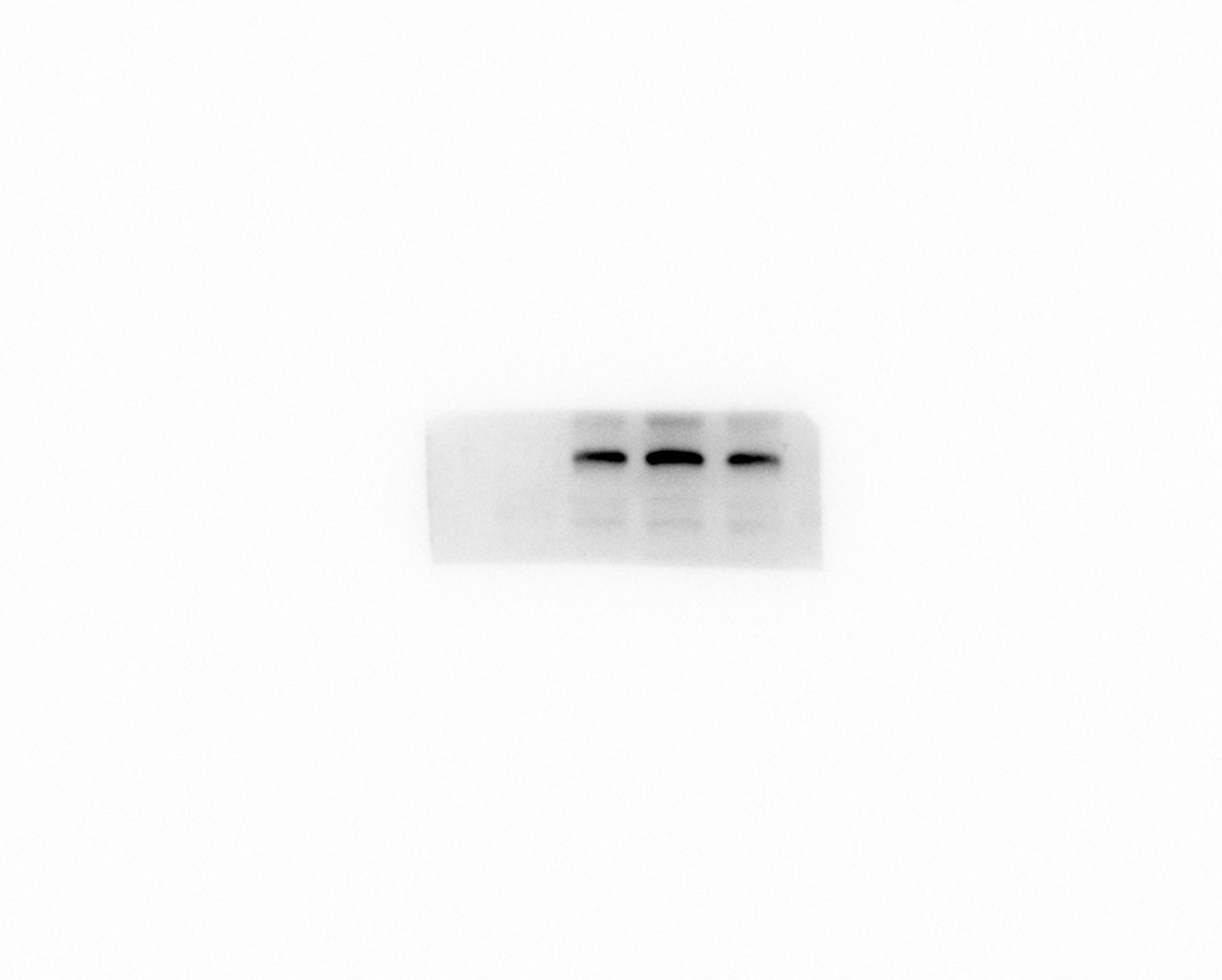

Supplement: Supplementary file 1 — Original western blots [file 41420_2025_2426_MOESM1_ESM.zip › fig1/a/fbp1.Tif]

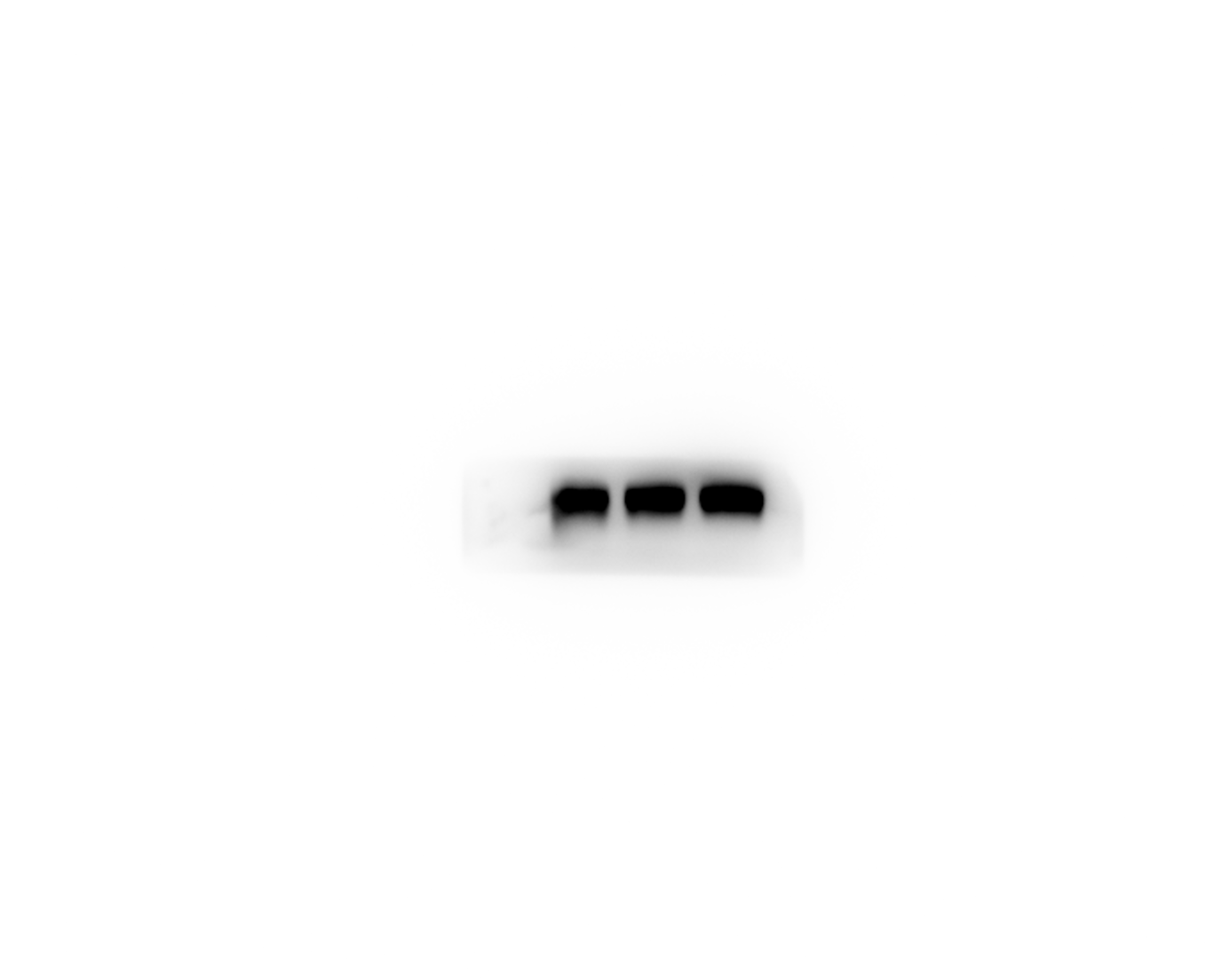

Supplement: Supplementary file 1 — Original western blots [file 41420_2025_2426_MOESM1_ESM.zip › fig1/a/tubulin.Tif]

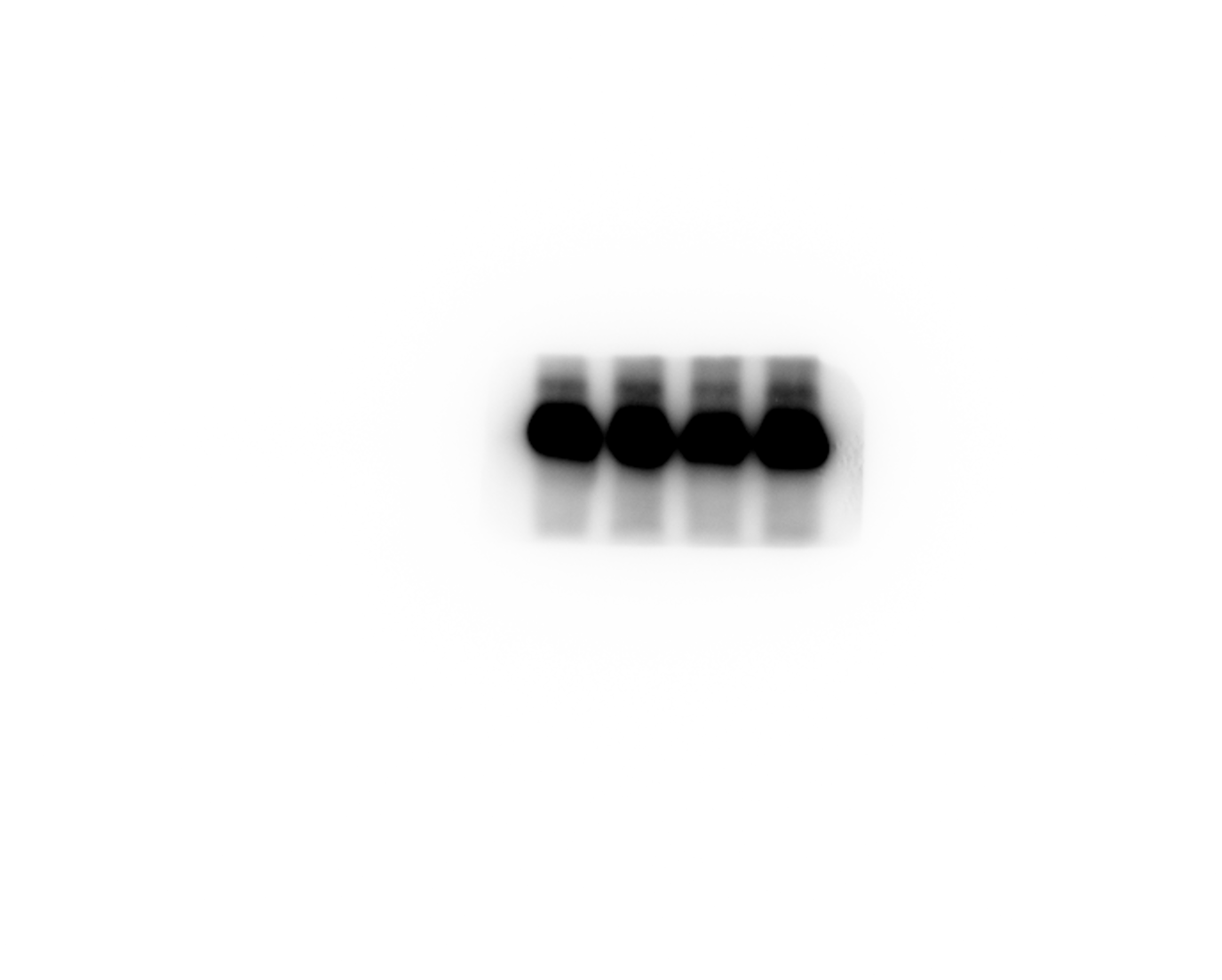

Supplement: Supplementary file 1 — Original western blots [file 41420_2025_2426_MOESM1_ESM.zip › fig1/b/flag.Tif]

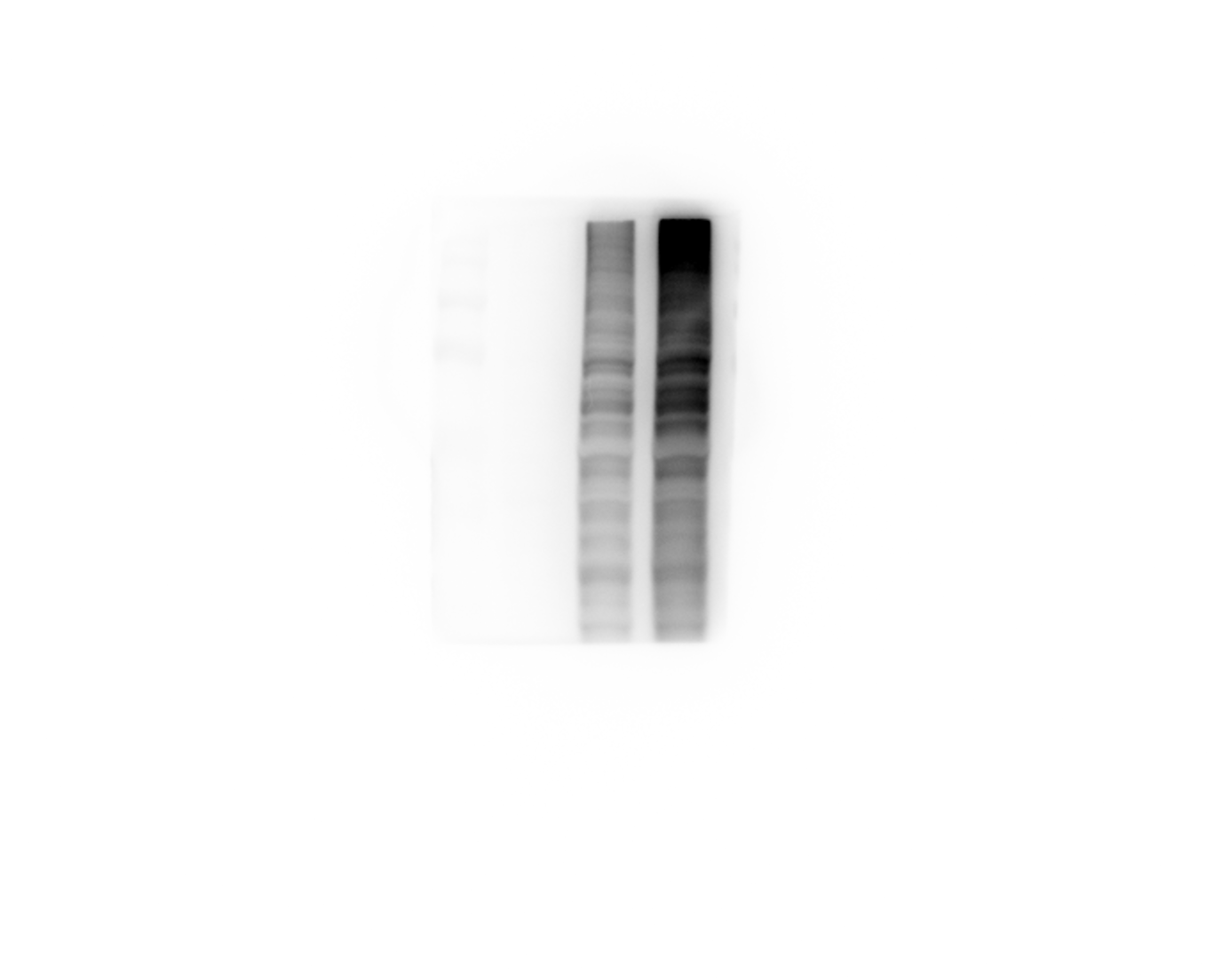

Supplement: Supplementary file 1 — Original western blots [file 41420_2025_2426_MOESM1_ESM.zip › fig1/b/HA-UB-3.Tif]

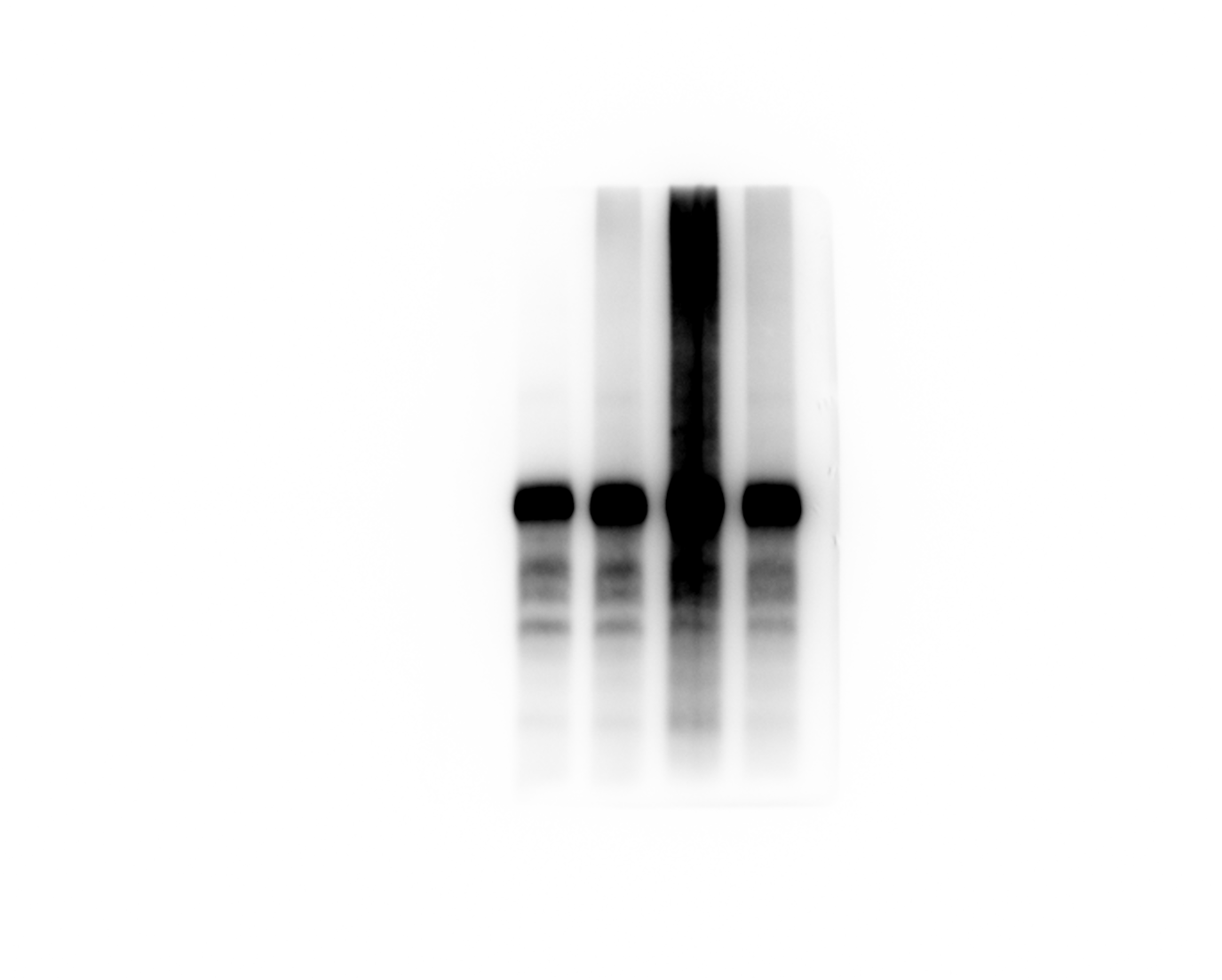

Supplement: Supplementary file 1 — Original western blots [file 41420_2025_2426_MOESM1_ESM.zip › fig1/b/ha.Tif]

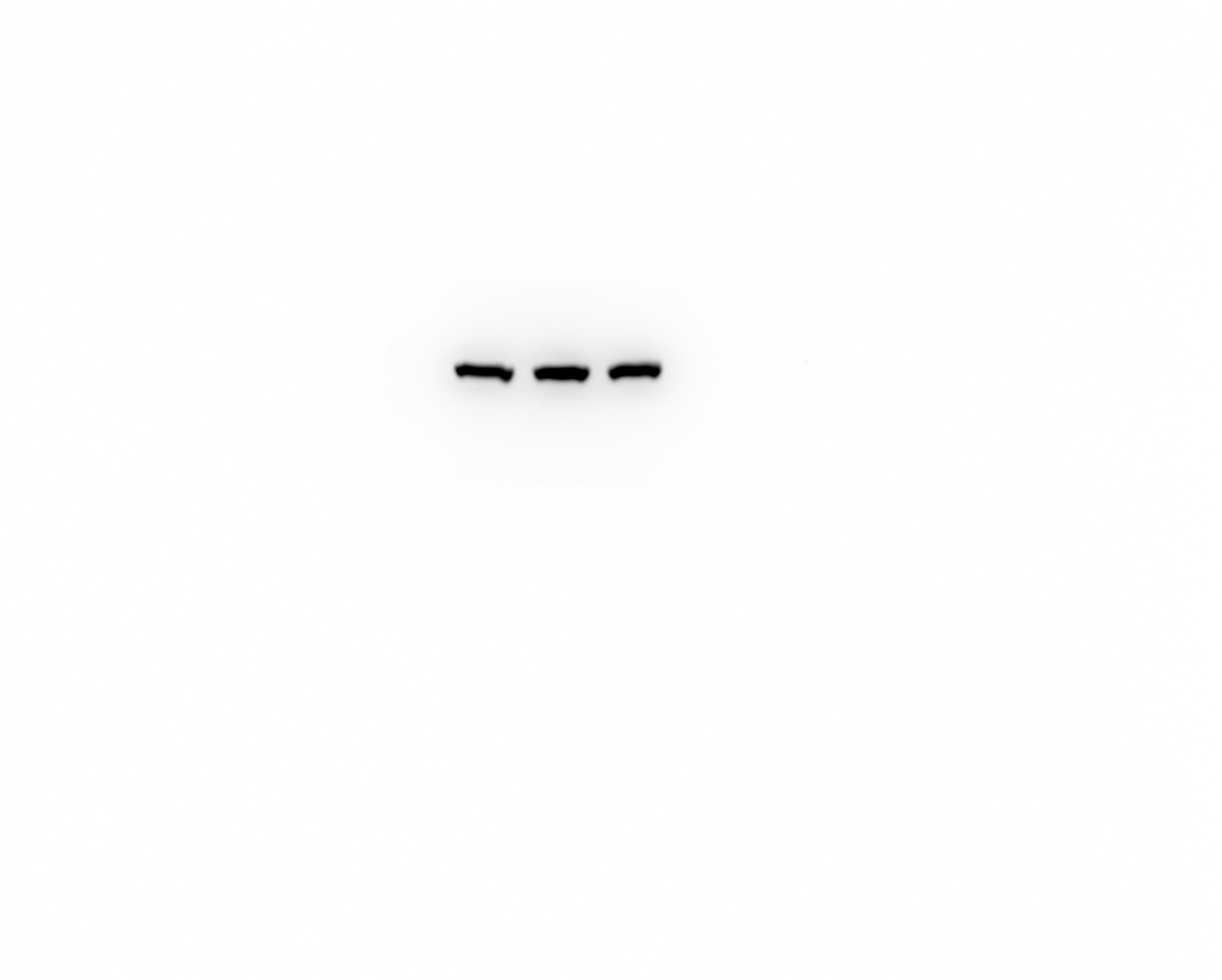

Supplement: Supplementary file 1 — Original western blots [file 41420_2025_2426_MOESM1_ESM.zip › fig1/b/Tublin.png]

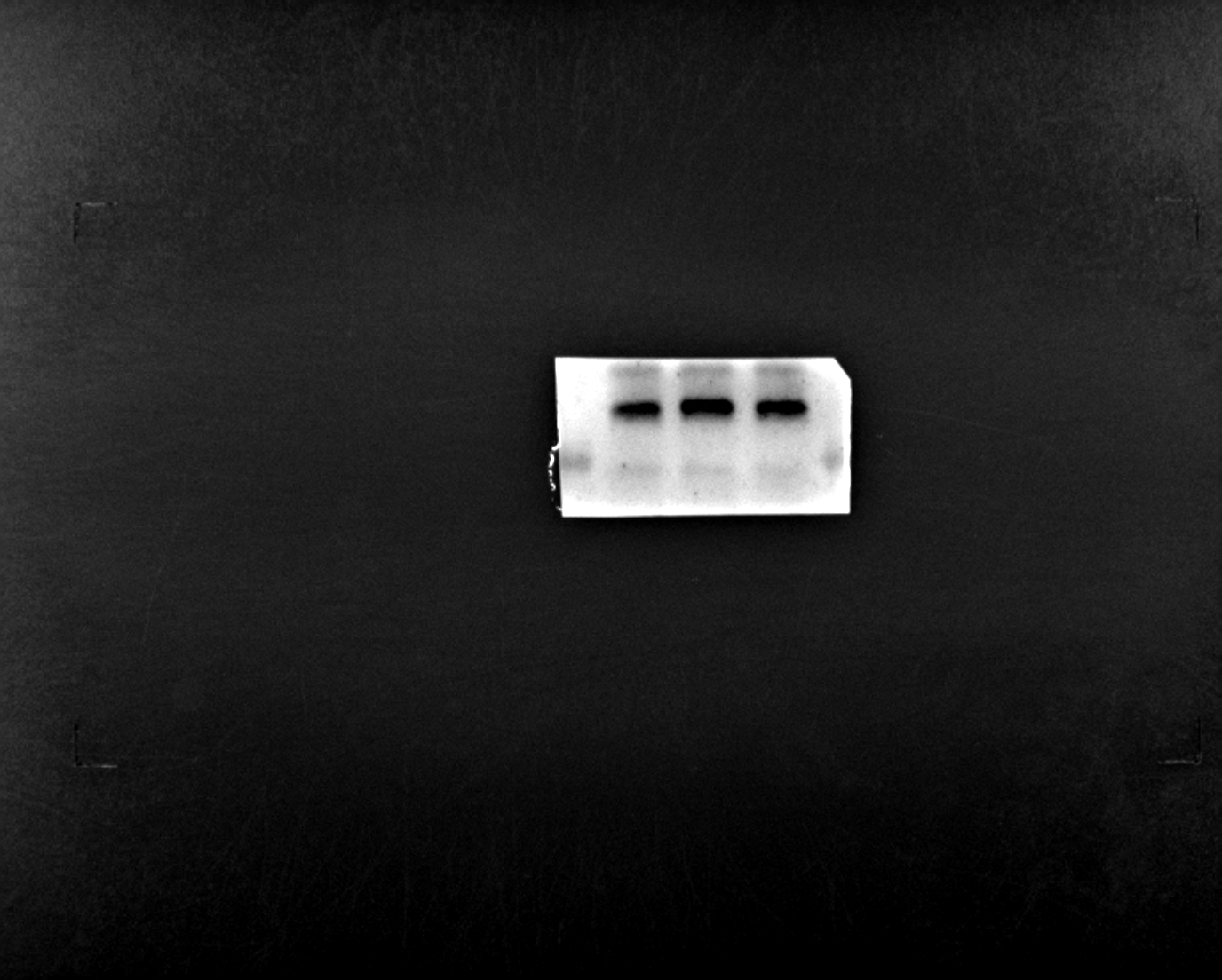

Supplement: Supplementary file 1 — Original western blots [file 41420_2025_2426_MOESM1_ESM.zip › fig1/c/fbp1-c.Tif]

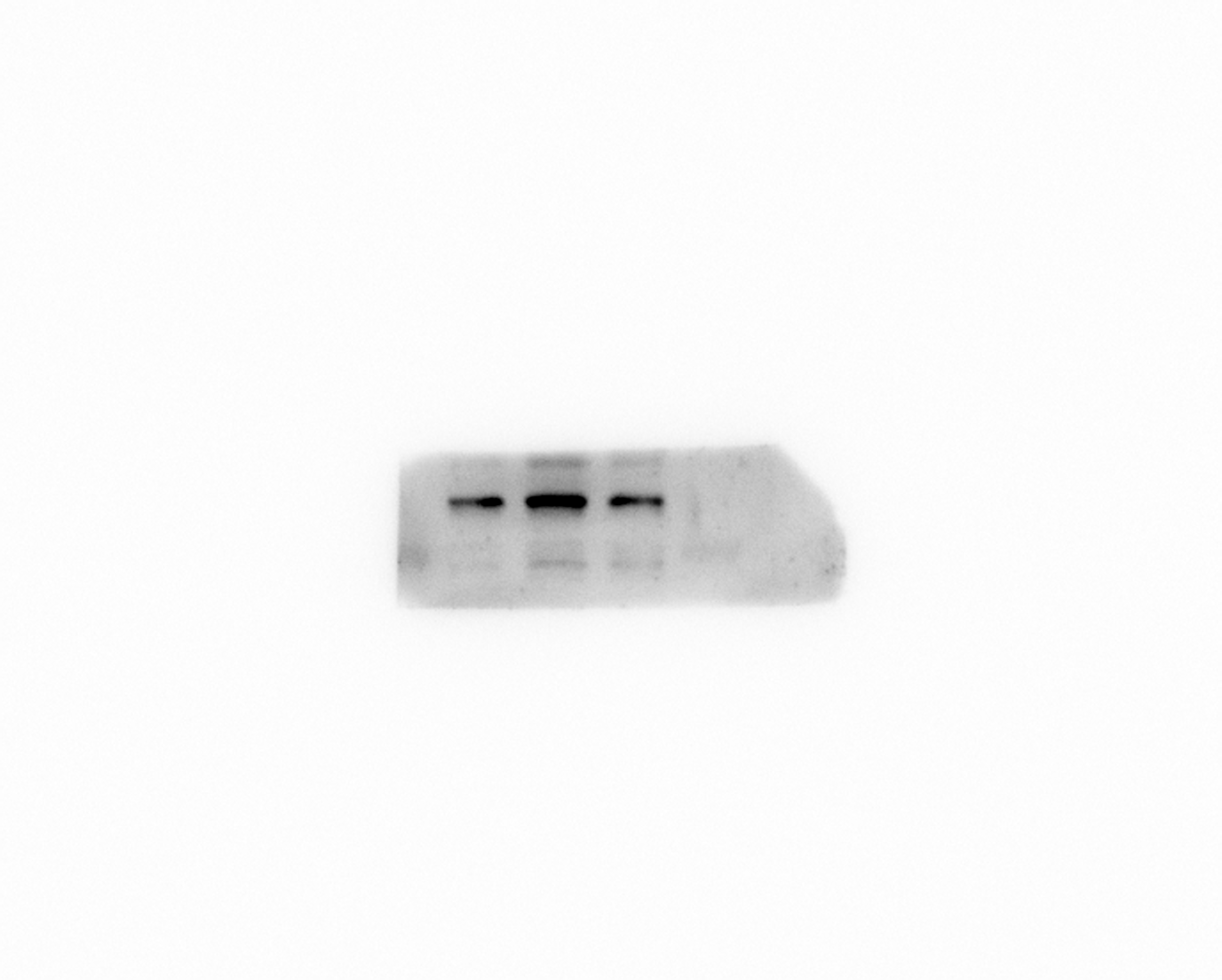

Supplement: Supplementary file 1 — Original western blots [file 41420_2025_2426_MOESM1_ESM.zip › fig1/c/fbp1-n.Tif]

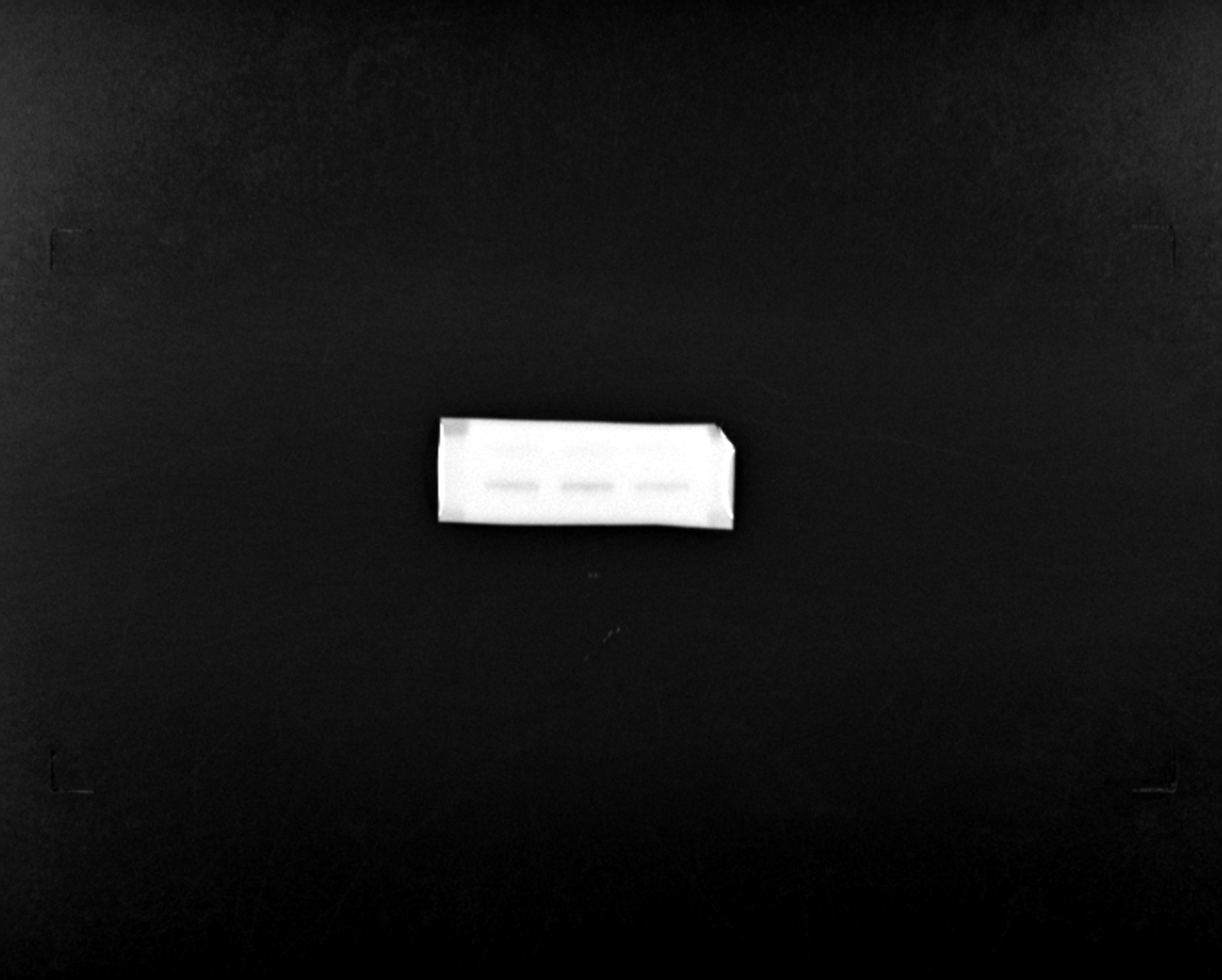

Supplement: Supplementary file 1 — Original western blots [file 41420_2025_2426_MOESM1_ESM.zip › fig1/c/lamin-c.Tif]

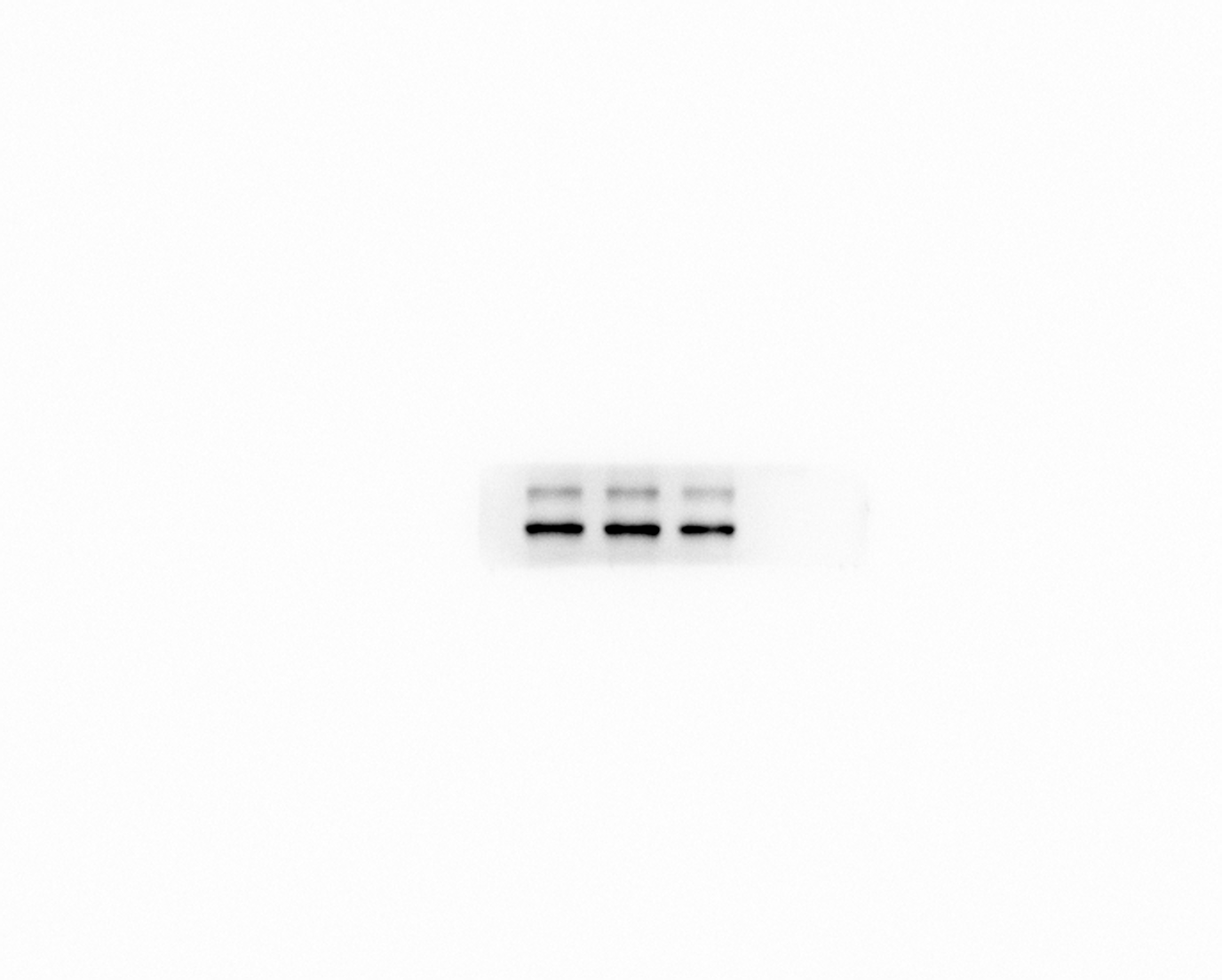

Supplement: Supplementary file 1 — Original western blots [file 41420_2025_2426_MOESM1_ESM.zip › fig1/c/lamin-n.Tif]

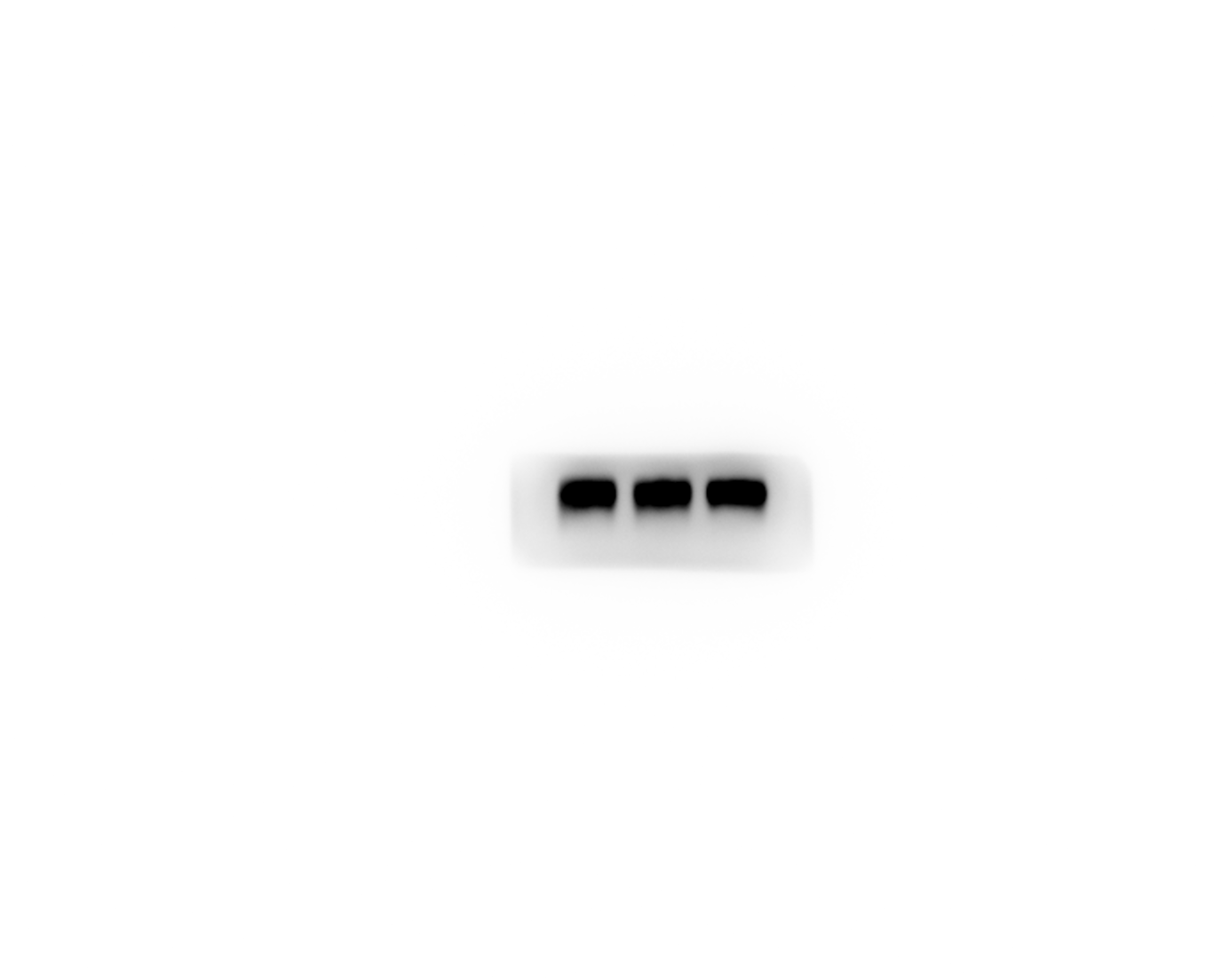

Supplement: Supplementary file 1 — Original western blots [file 41420_2025_2426_MOESM1_ESM.zip › fig1/c/tubulin-c.Tif]

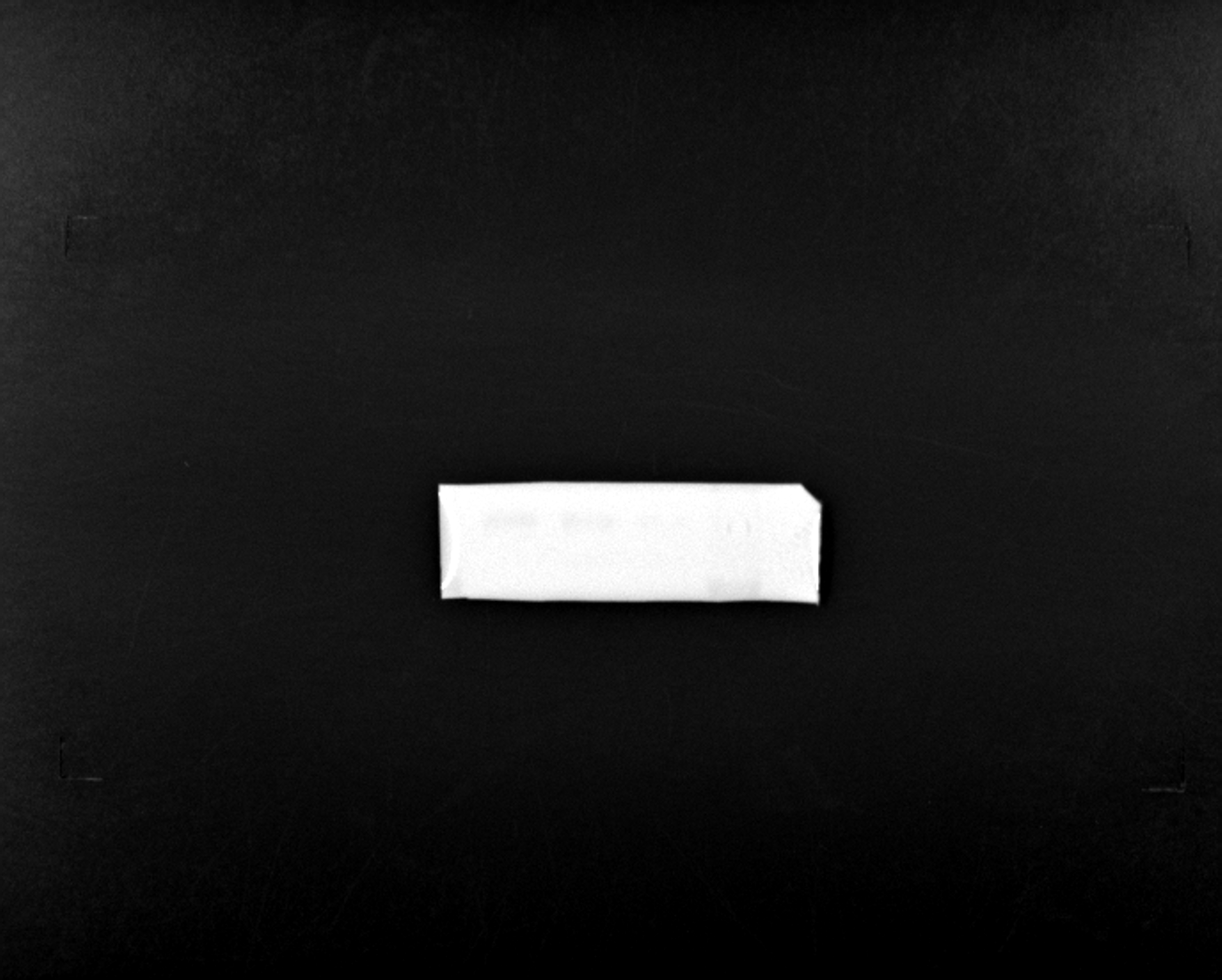

Supplement: Supplementary file 1 — Original western blots [file 41420_2025_2426_MOESM1_ESM.zip › fig1/c/tubulin-n.Tif]

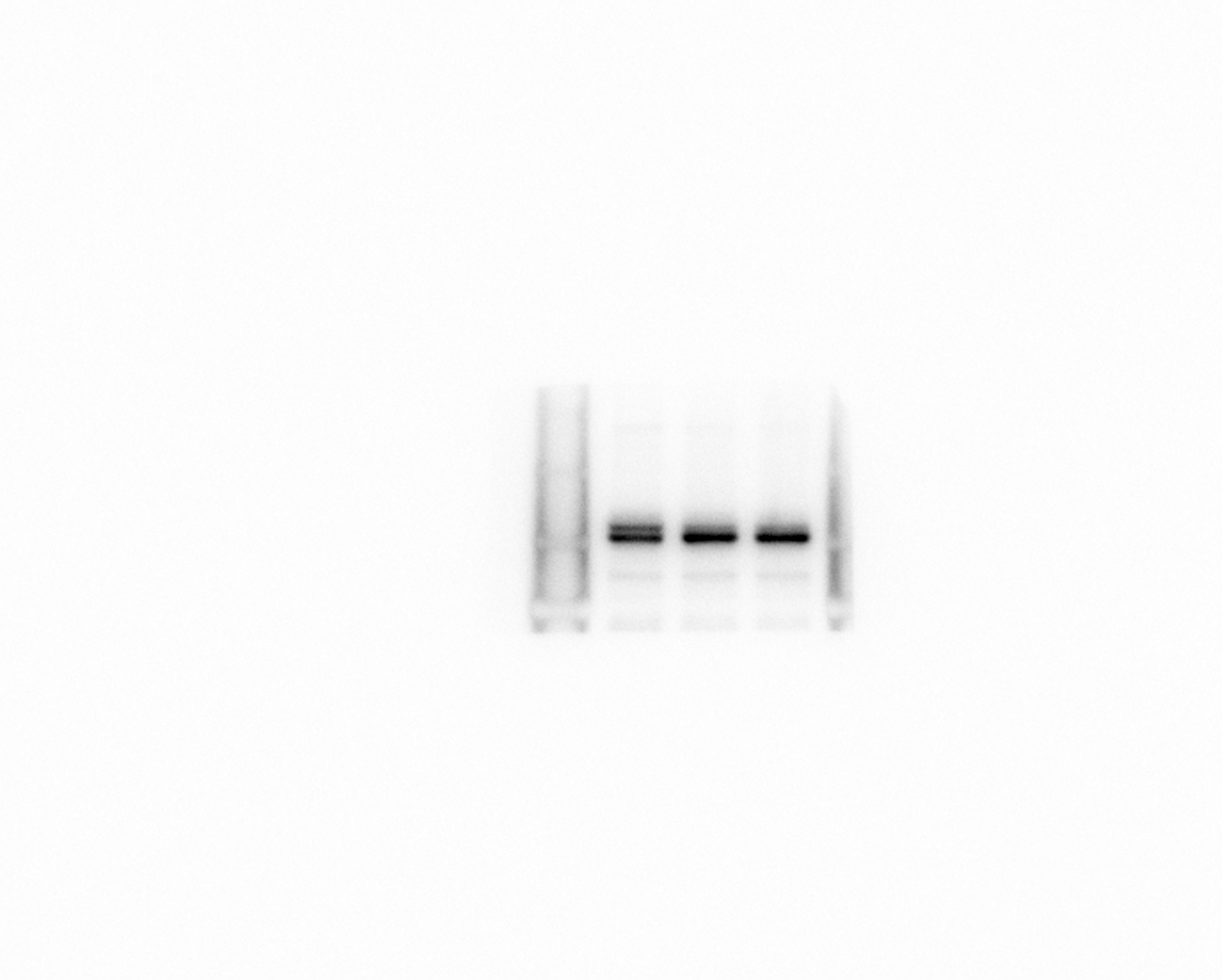

Supplement: Supplementary file 1 — Original western blots [file 41420_2025_2426_MOESM1_ESM.zip › fig2/a/cul4b-c.Tif]

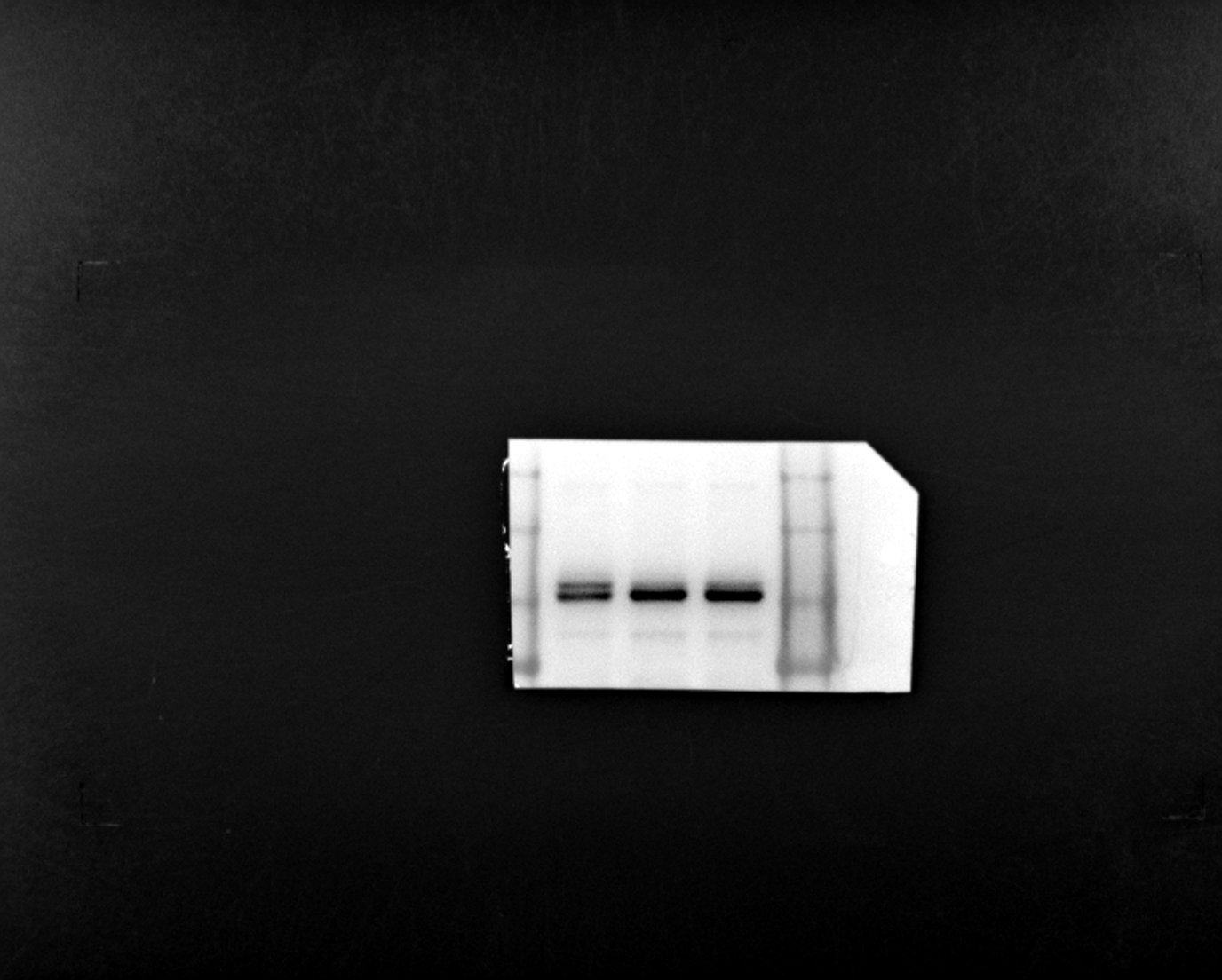

Supplement: Supplementary file 1 — Original western blots [file 41420_2025_2426_MOESM1_ESM.zip › fig2/a/cul4b.Tif]

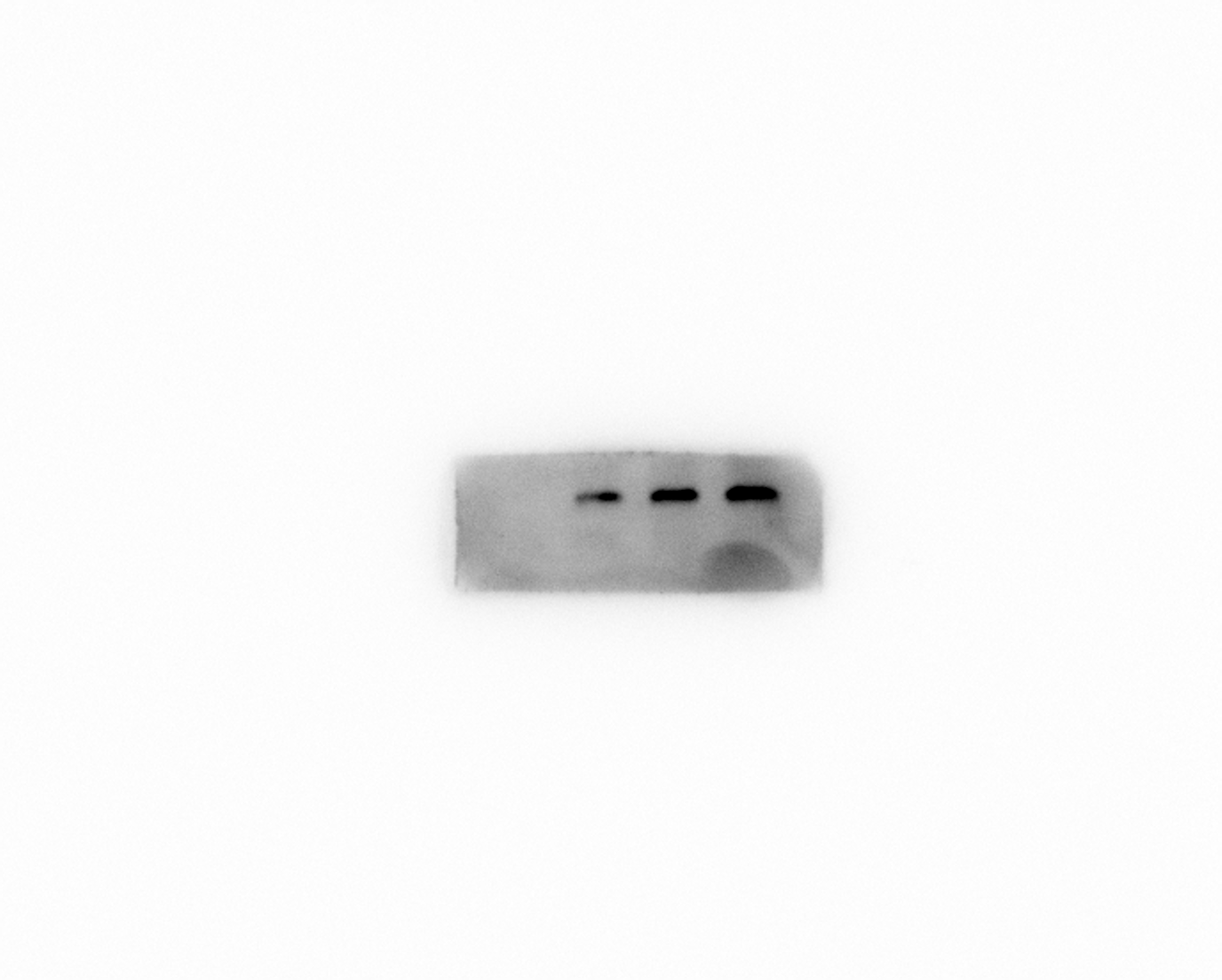

Supplement: Supplementary file 1 — Original western blots [file 41420_2025_2426_MOESM1_ESM.zip › fig2/a/fbp1-c.Tif]

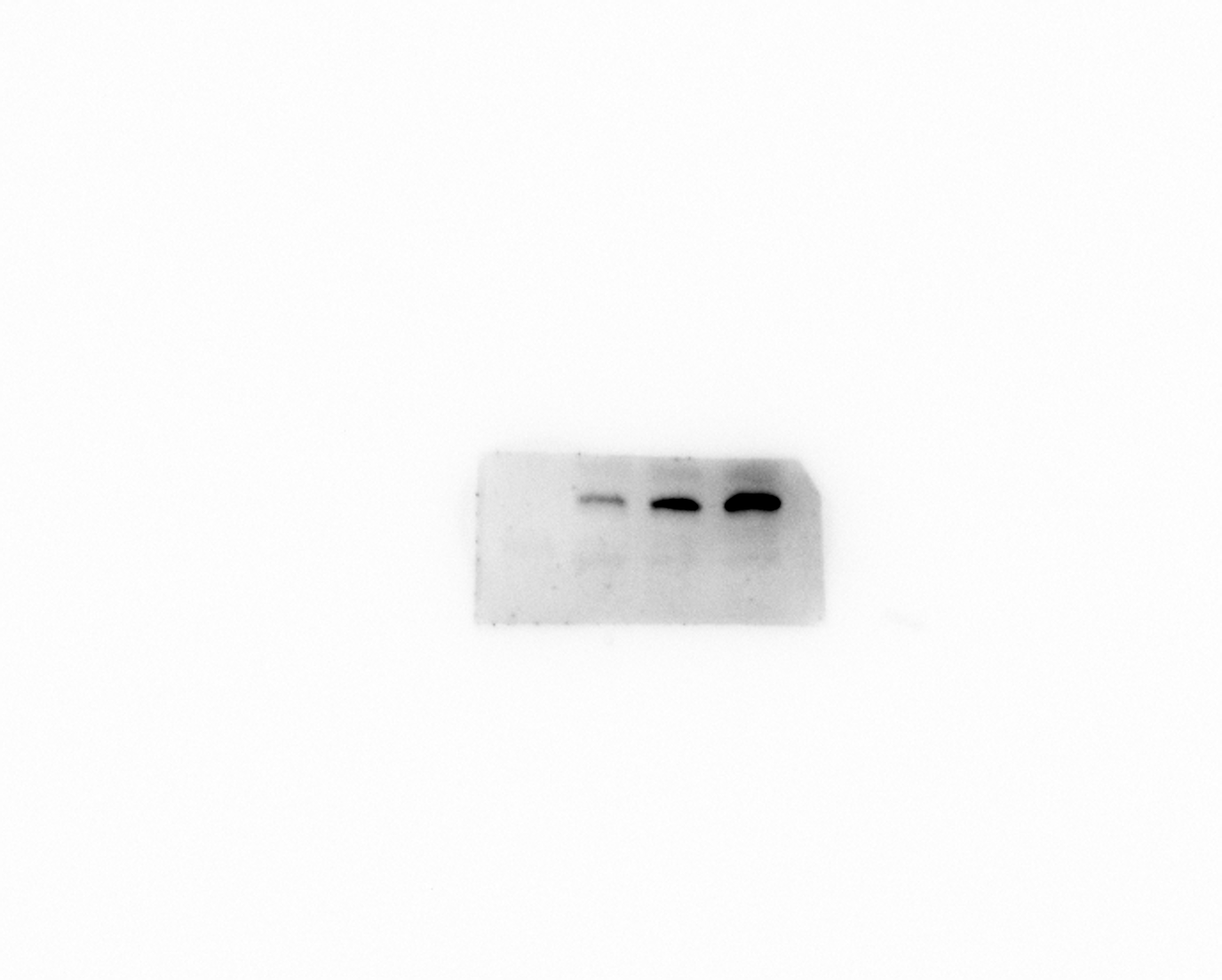

Supplement: Supplementary file 1 — Original western blots [file 41420_2025_2426_MOESM1_ESM.zip › fig2/a/fbp1.Tif]

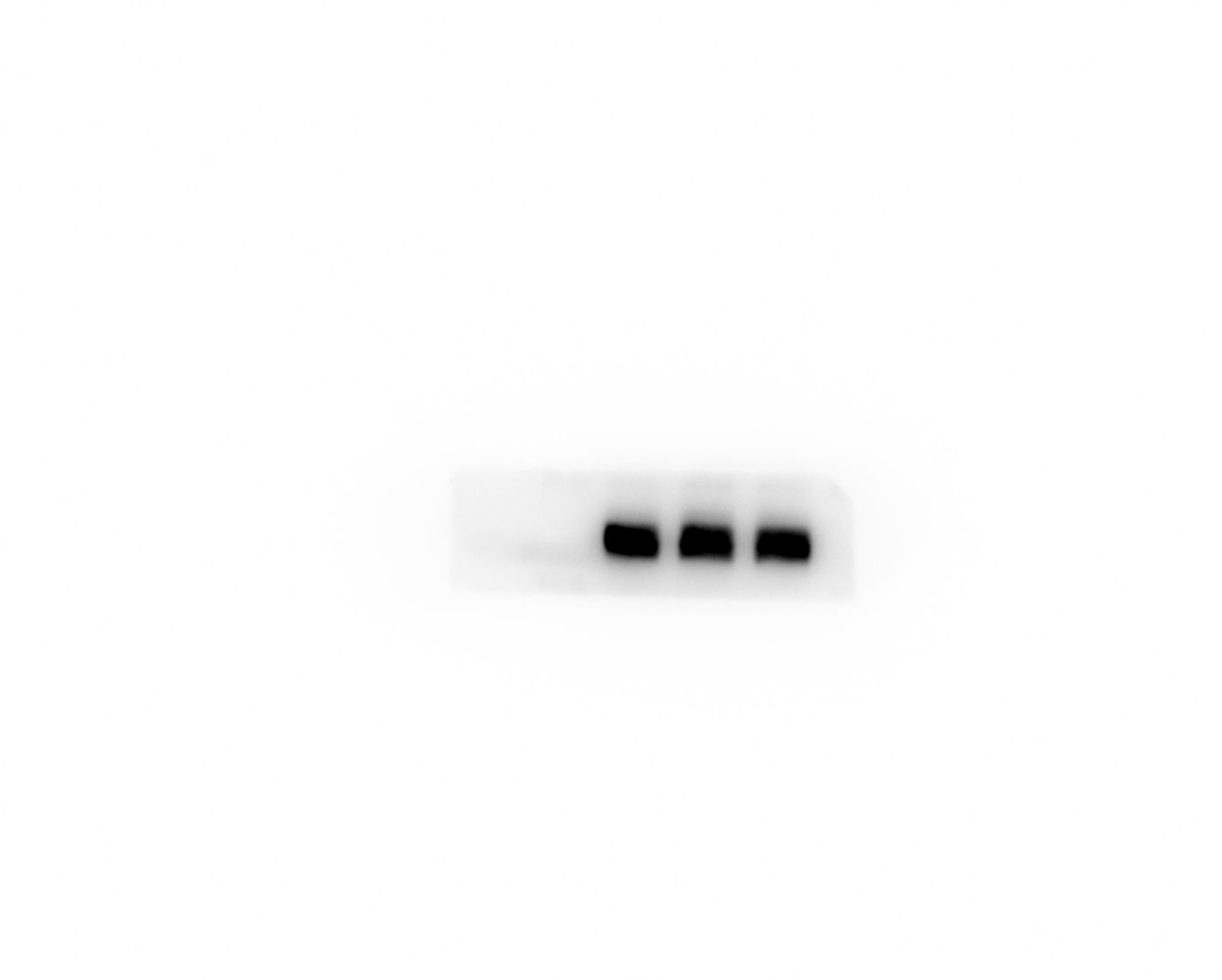

Supplement: Supplementary file 1 — Original western blots [file 41420_2025_2426_MOESM1_ESM.zip › fig2/a/tubulin-c.Tif]

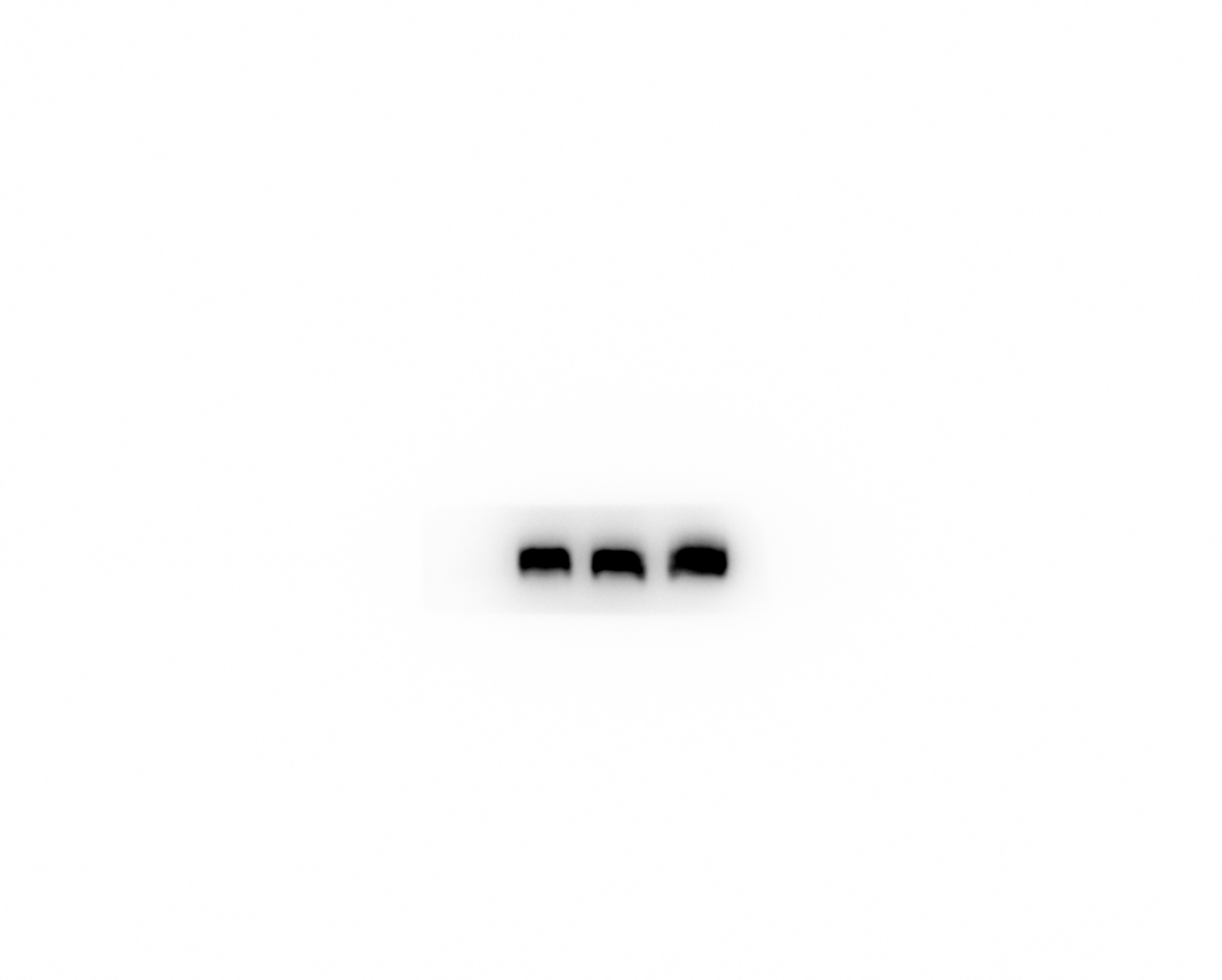

Supplement: Supplementary file 1 — Original western blots [file 41420_2025_2426_MOESM1_ESM.zip › fig2/a/tubulin.Tif]

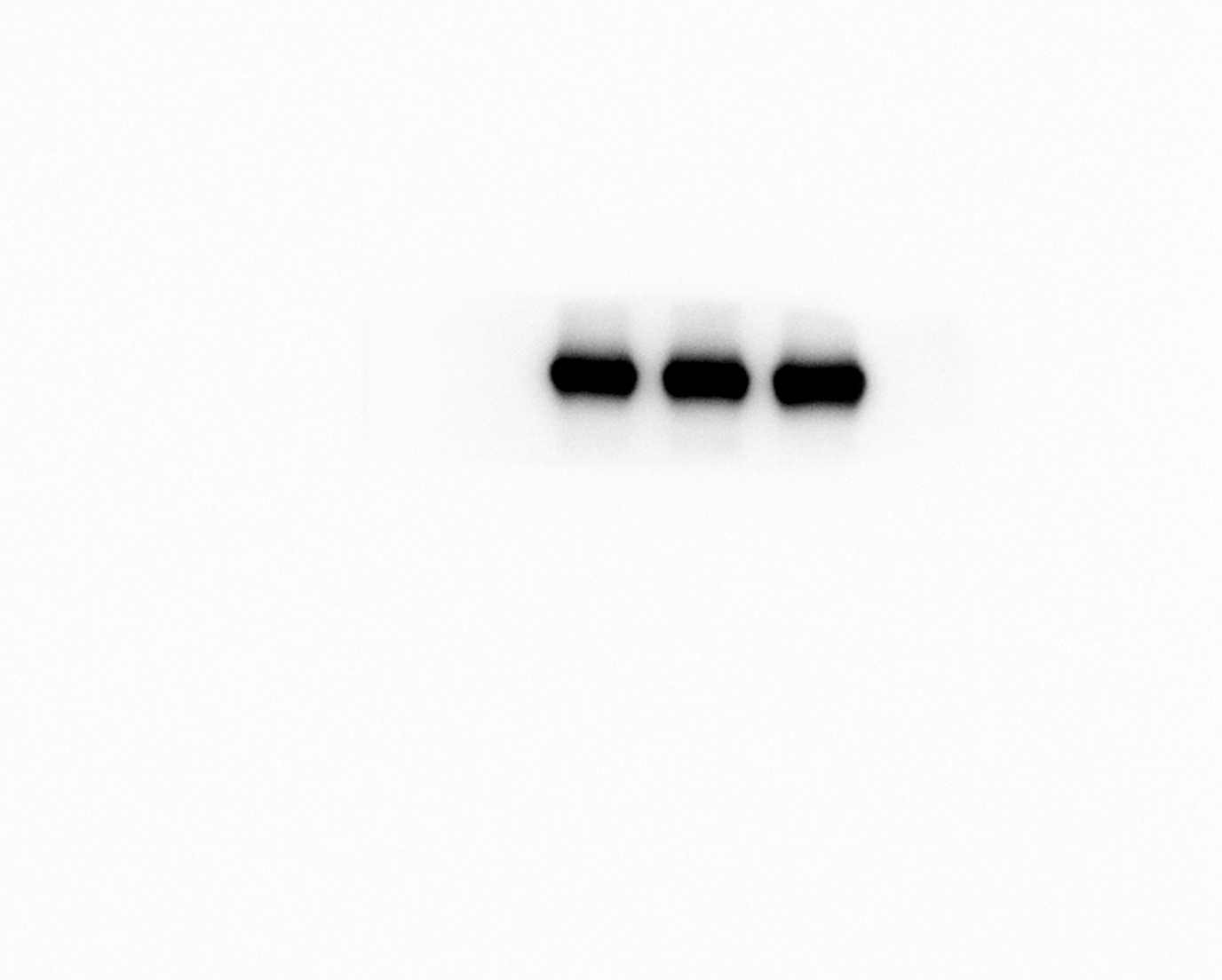

Supplement: Supplementary file 1 — Original western blots [file 41420_2025_2426_MOESM1_ESM.zip › fig2/b/flag.Tif]

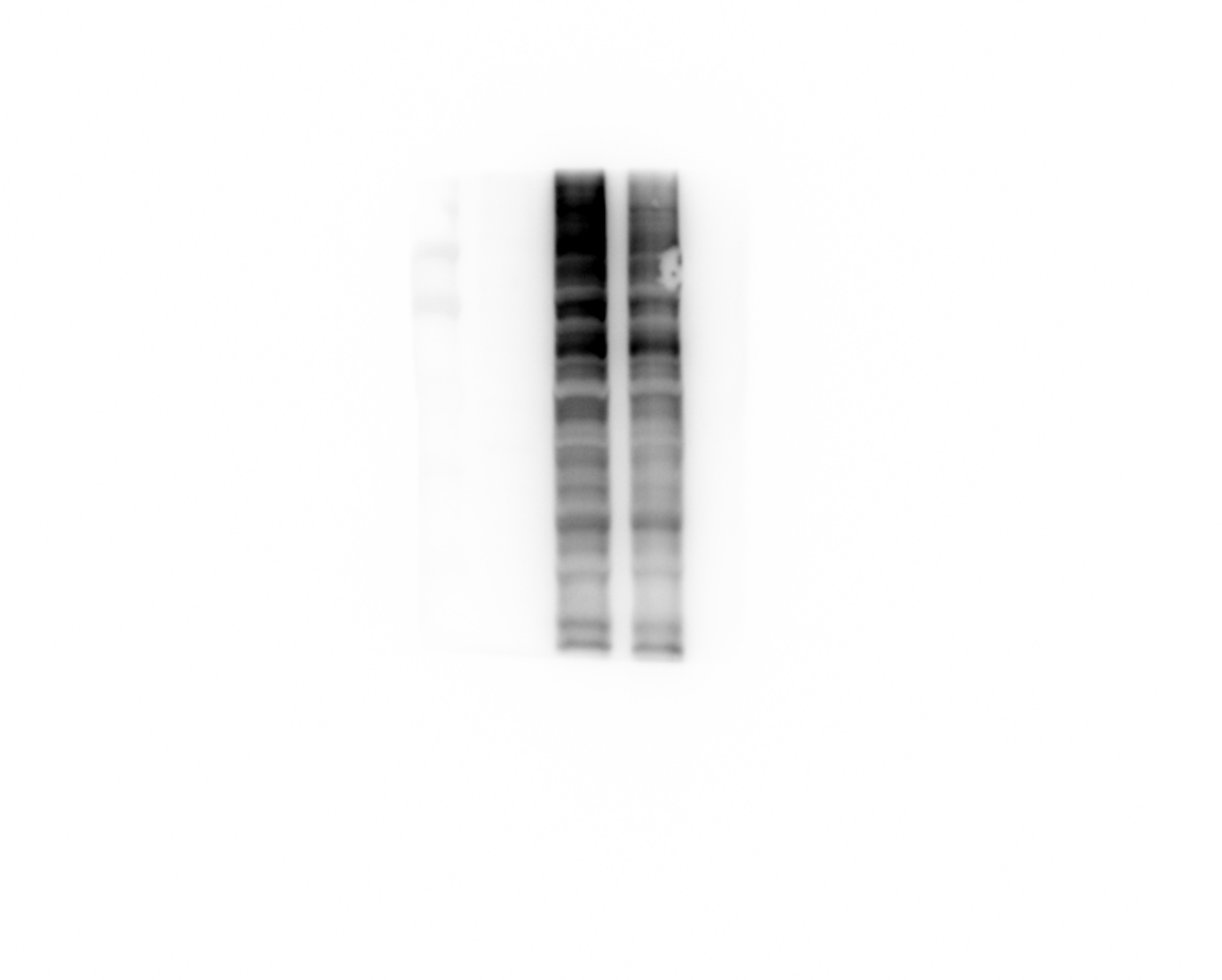

Supplement: Supplementary file 1 — Original western blots [file 41420_2025_2426_MOESM1_ESM.zip › fig2/b/HA-UB-2.Tif]

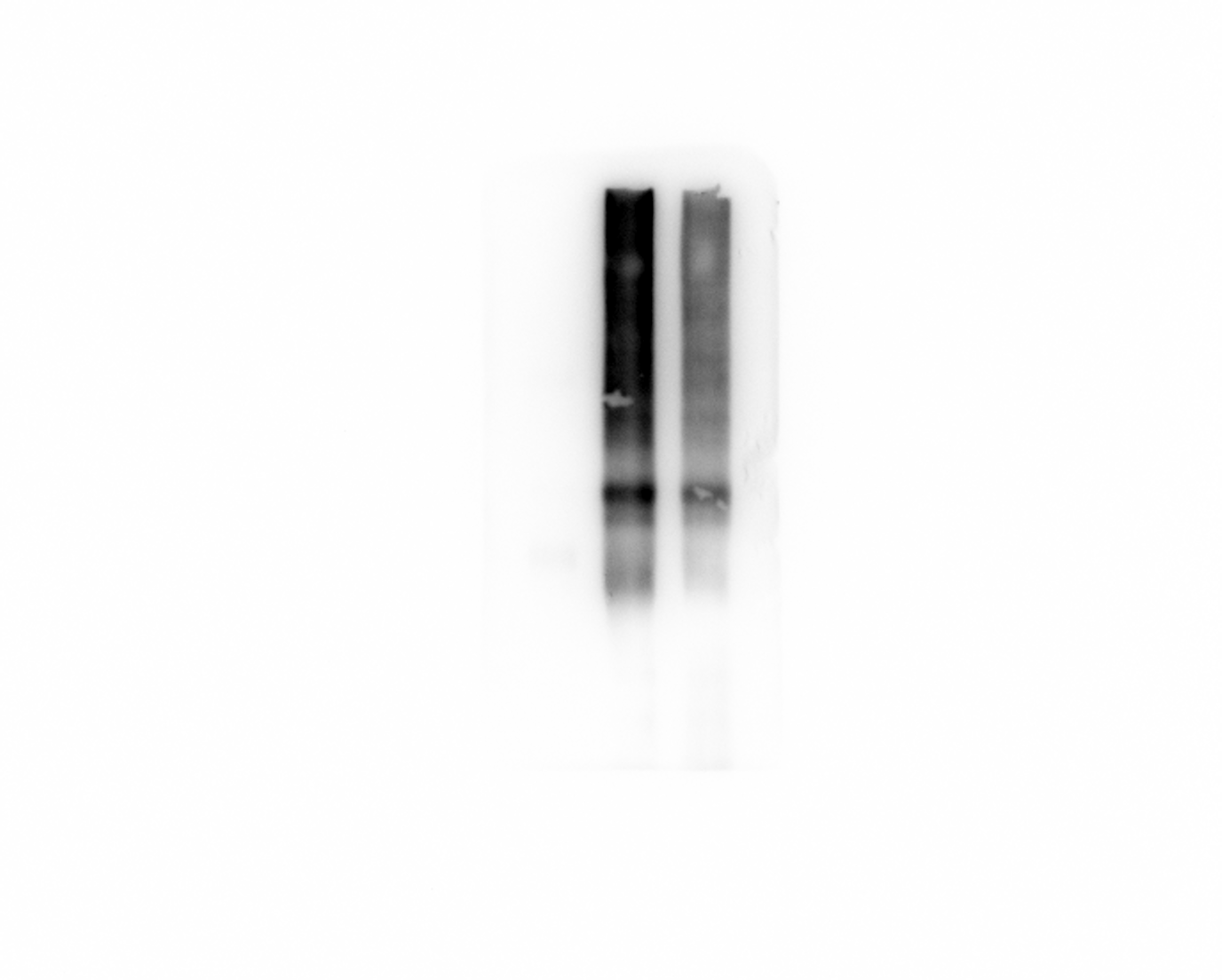

Supplement: Supplementary file 1 — Original western blots [file 41420_2025_2426_MOESM1_ESM.zip › fig2/b/ha.Tif]

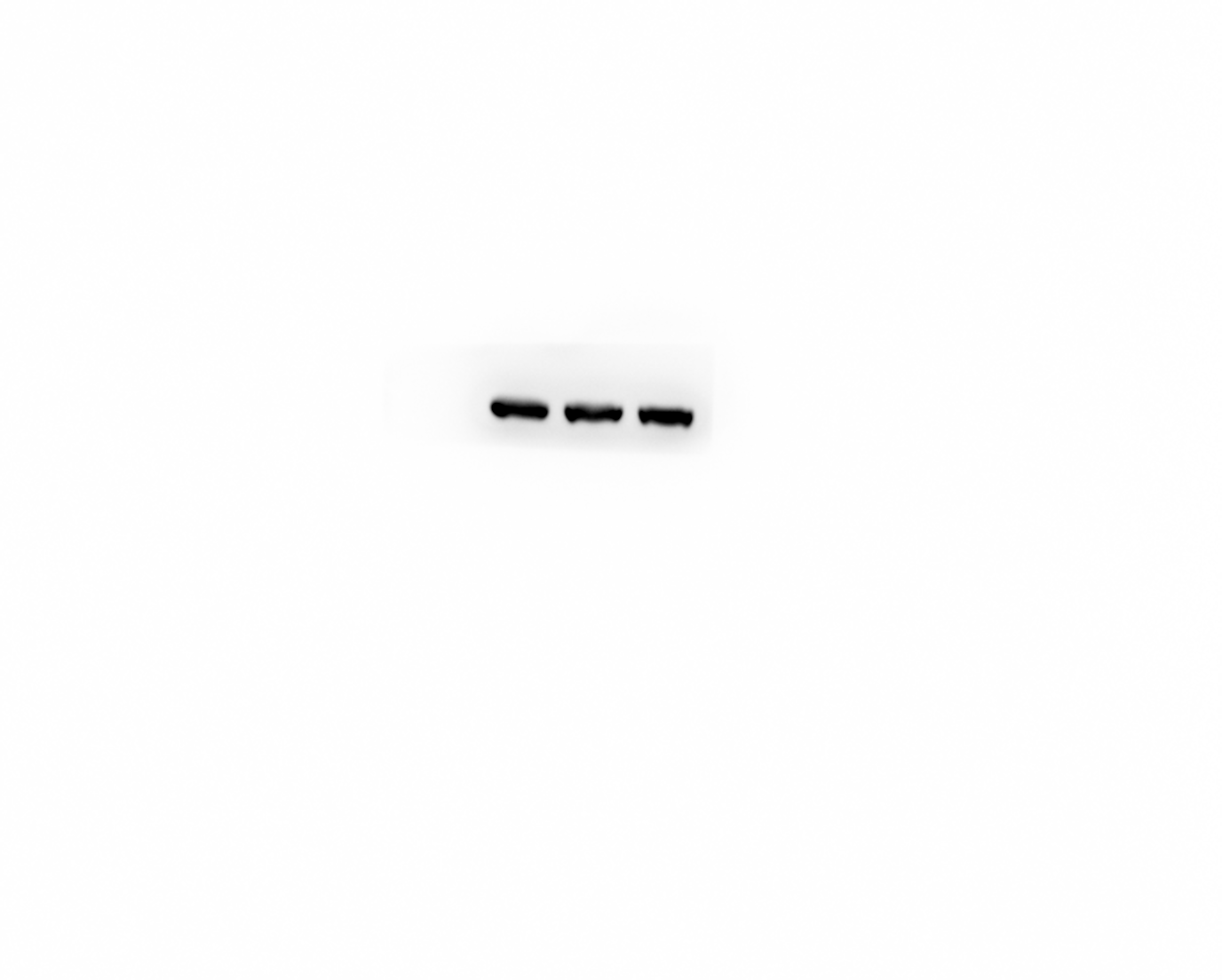

Supplement: Supplementary file 1 — Original western blots [file 41420_2025_2426_MOESM1_ESM.zip › fig2/b/Tubulin.Tif]

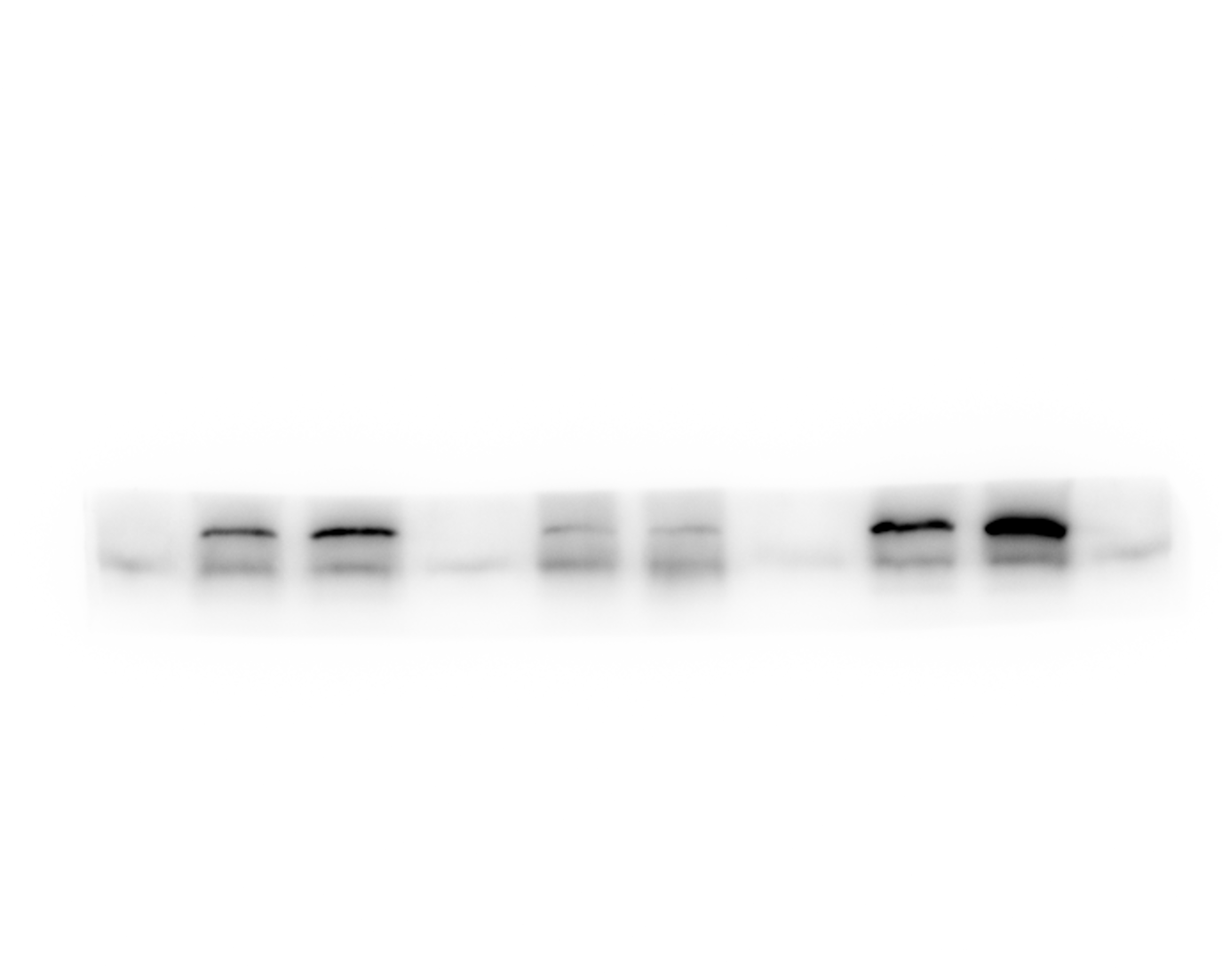

Supplement: Supplementary file 1 — Original western blots [file 41420_2025_2426_MOESM1_ESM.zip › fig2/c/fbp1.Tif]

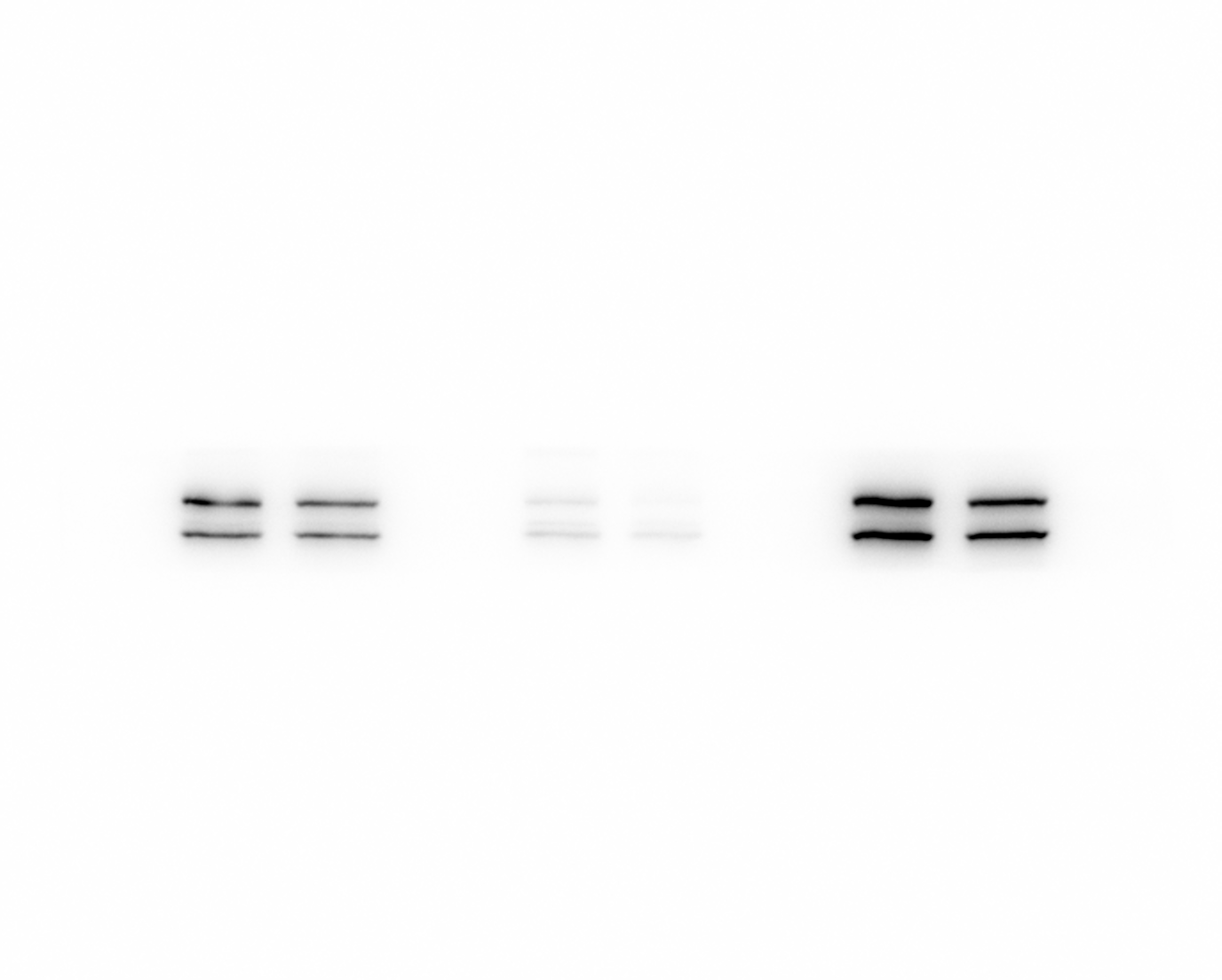

Supplement: Supplementary file 1 — Original western blots [file 41420_2025_2426_MOESM1_ESM.zip › fig2/c/lamin.Tif]

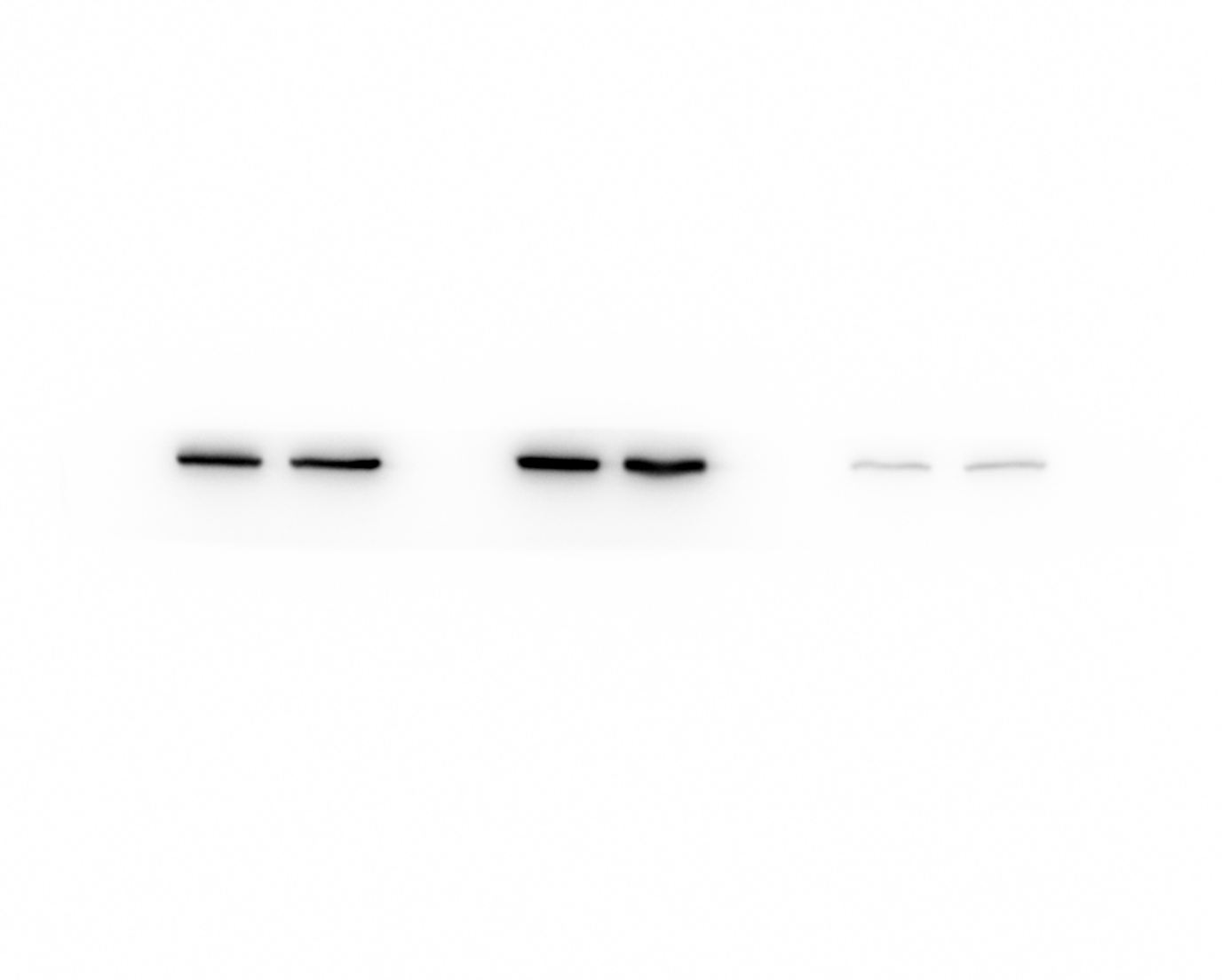

Supplement: Supplementary file 1 — Original western blots [file 41420_2025_2426_MOESM1_ESM.zip › fig2/c/tubulin.Tif]

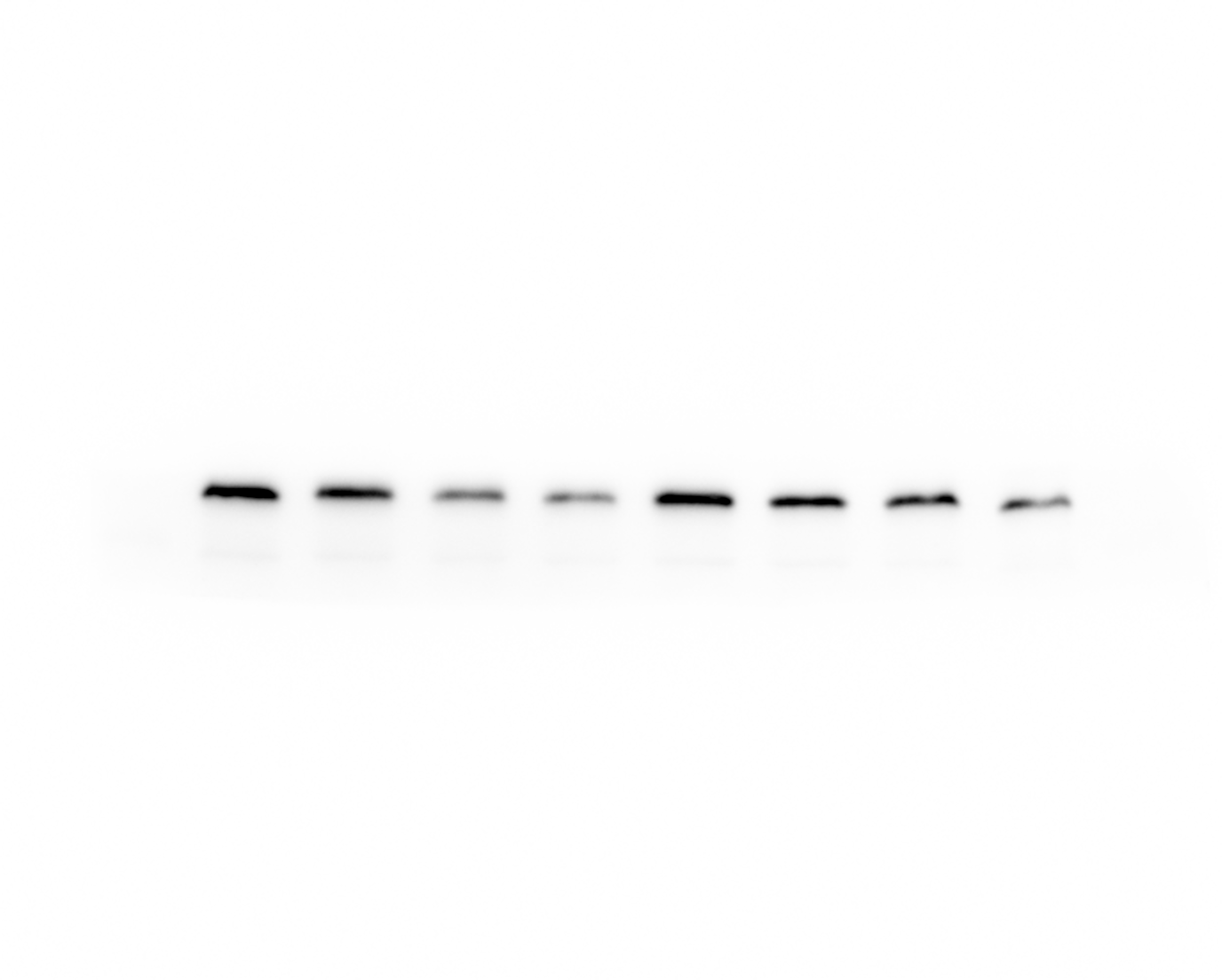

Supplement: Supplementary file 1 — Original western blots [file 41420_2025_2426_MOESM1_ESM.zip › fig2/d/fbp1.Tif]

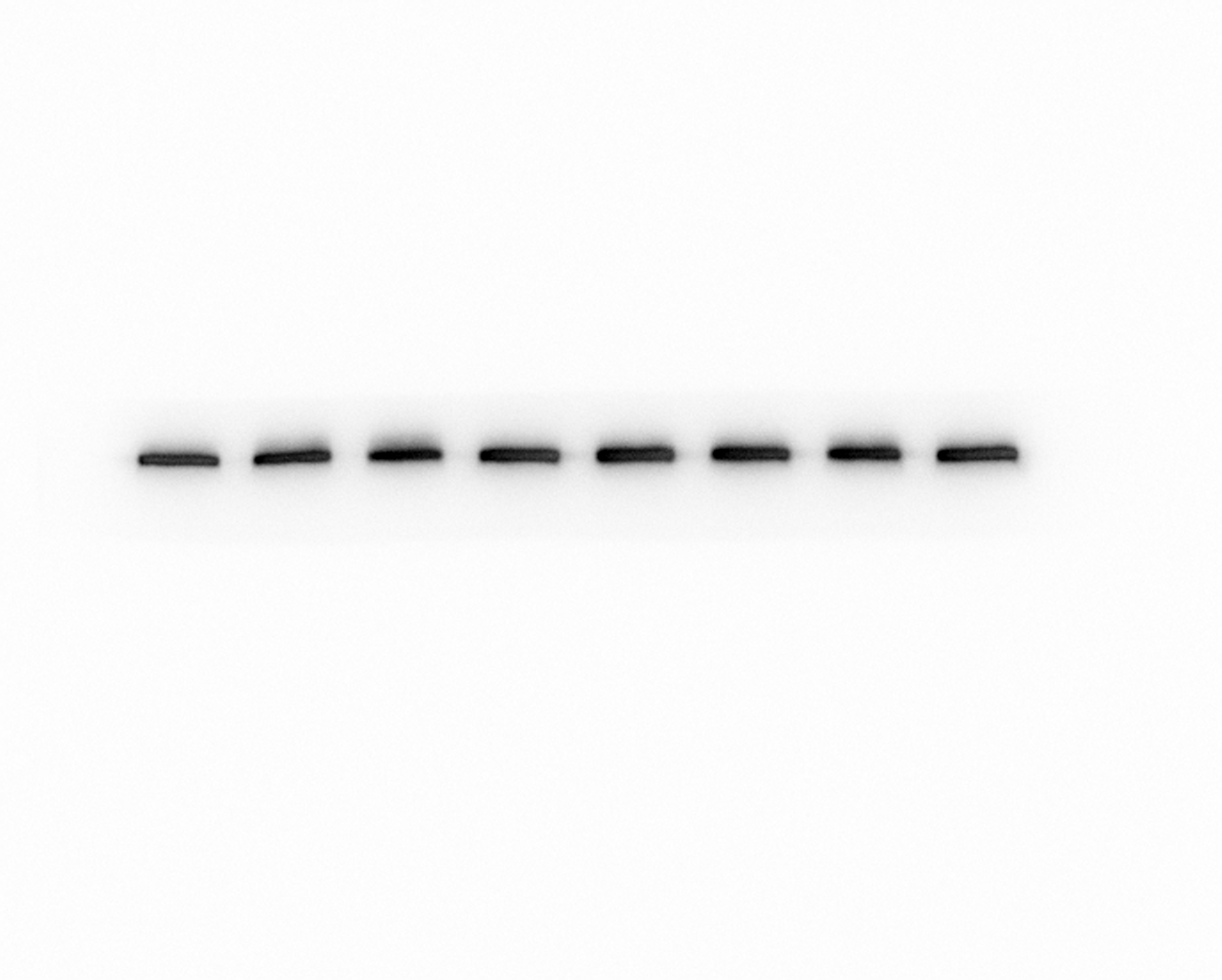

Supplement: Supplementary file 1 — Original western blots [file 41420_2025_2426_MOESM1_ESM.zip › fig2/d/tubulin.Tif]

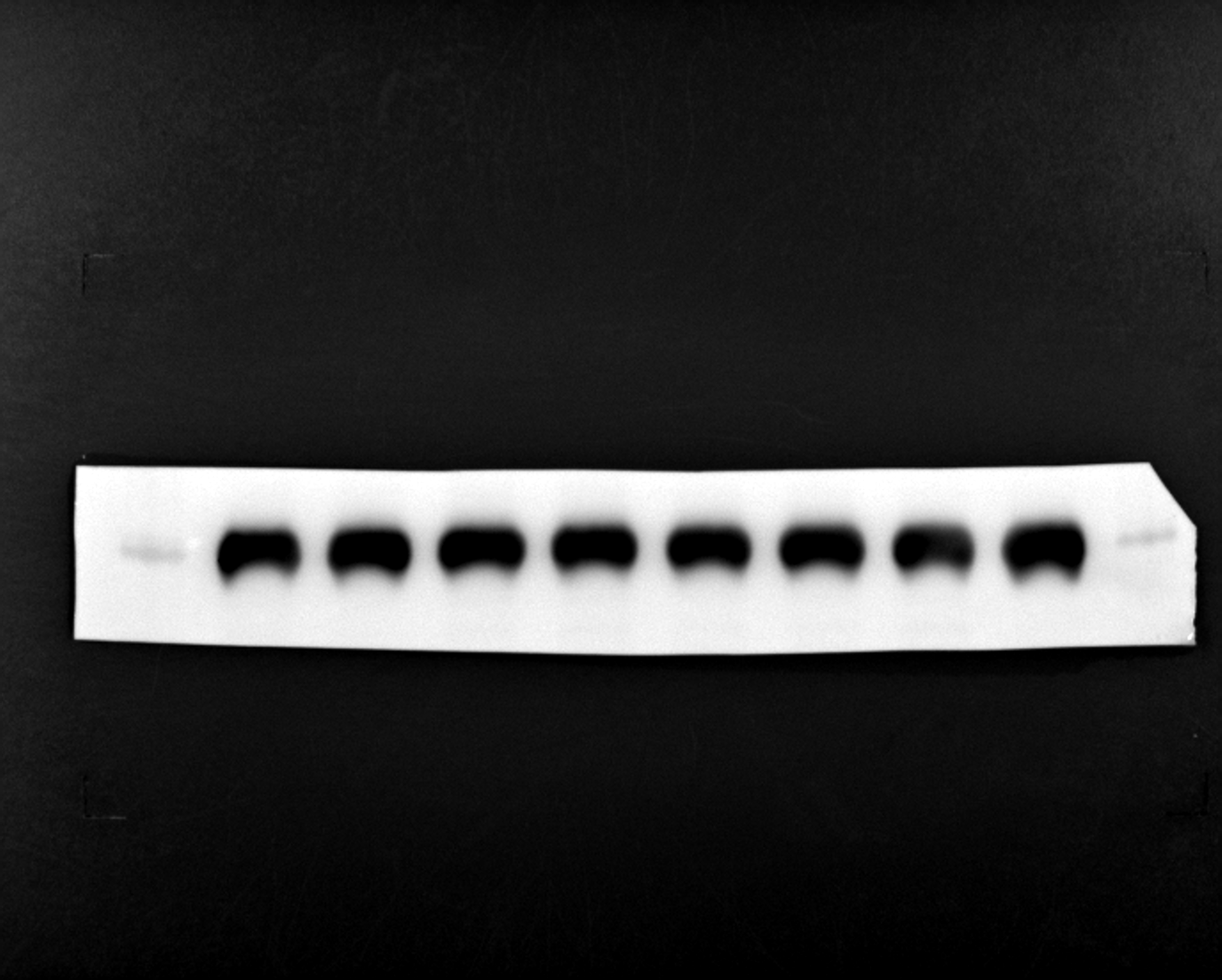

Supplement: Supplementary file 1 — Original western blots [file 41420_2025_2426_MOESM1_ESM.zip › fig3/a/input-flag.Tif]

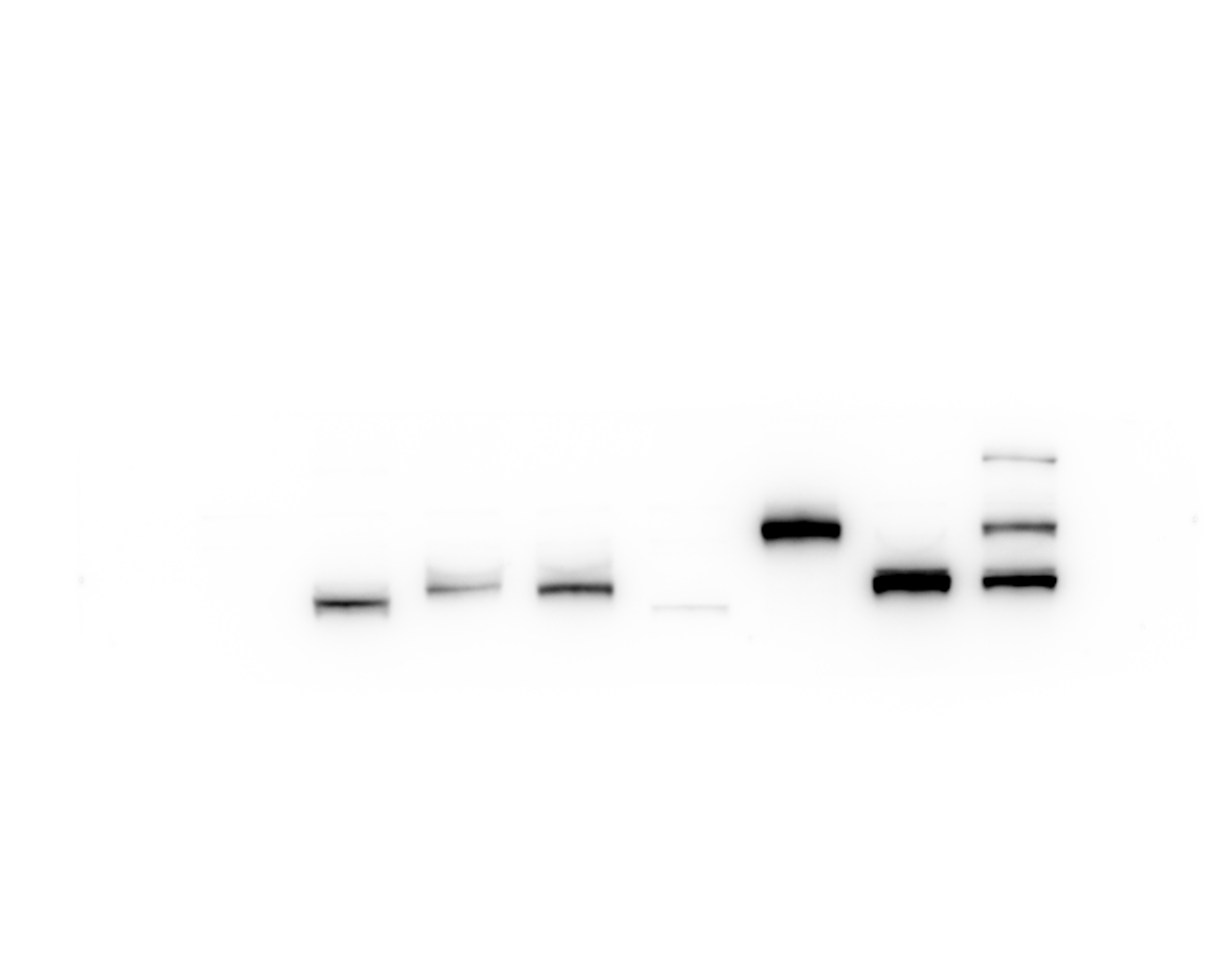

Supplement: Supplementary file 1 — Original western blots [file 41420_2025_2426_MOESM1_ESM.zip › fig3/a/input-myc.Tif]

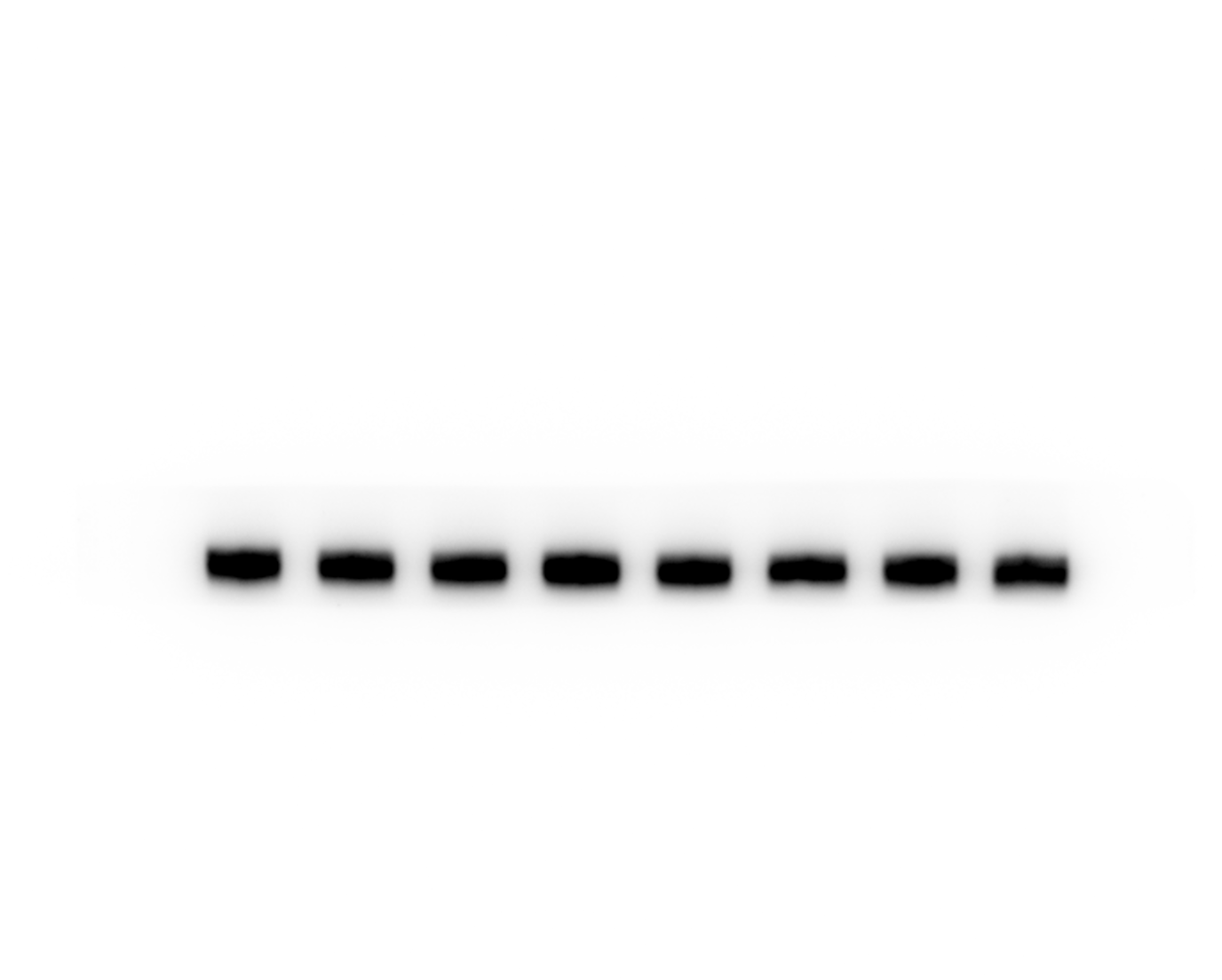

Supplement: Supplementary file 1 — Original western blots [file 41420_2025_2426_MOESM1_ESM.zip › fig3/a/input-tubulin.Tif]

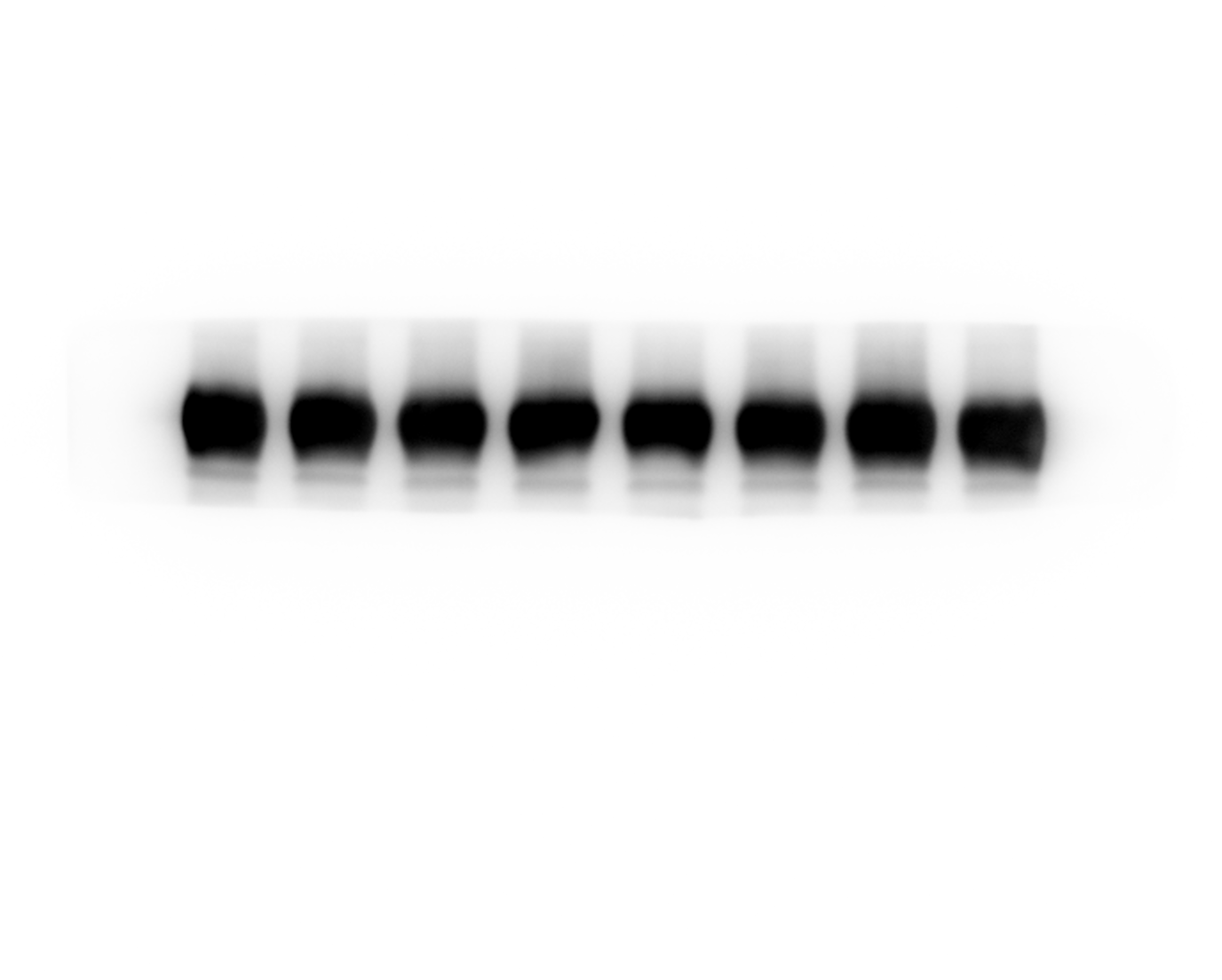

Supplement: Supplementary file 1 — Original western blots [file 41420_2025_2426_MOESM1_ESM.zip › fig3/a/ip-flag.Tif]

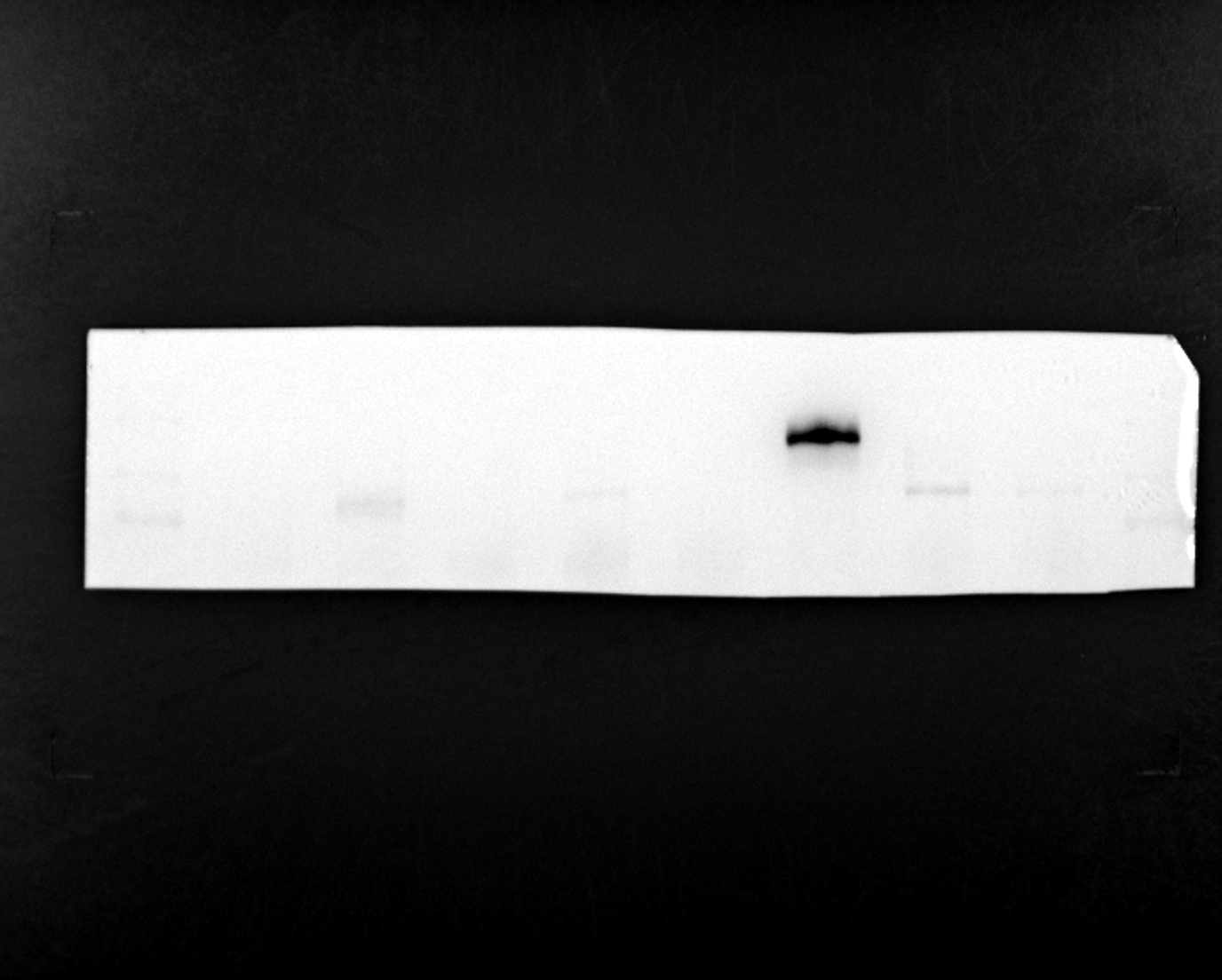

Supplement: Supplementary file 1 — Original western blots [file 41420_2025_2426_MOESM1_ESM.zip › fig3/a/ip-myc.Tif]

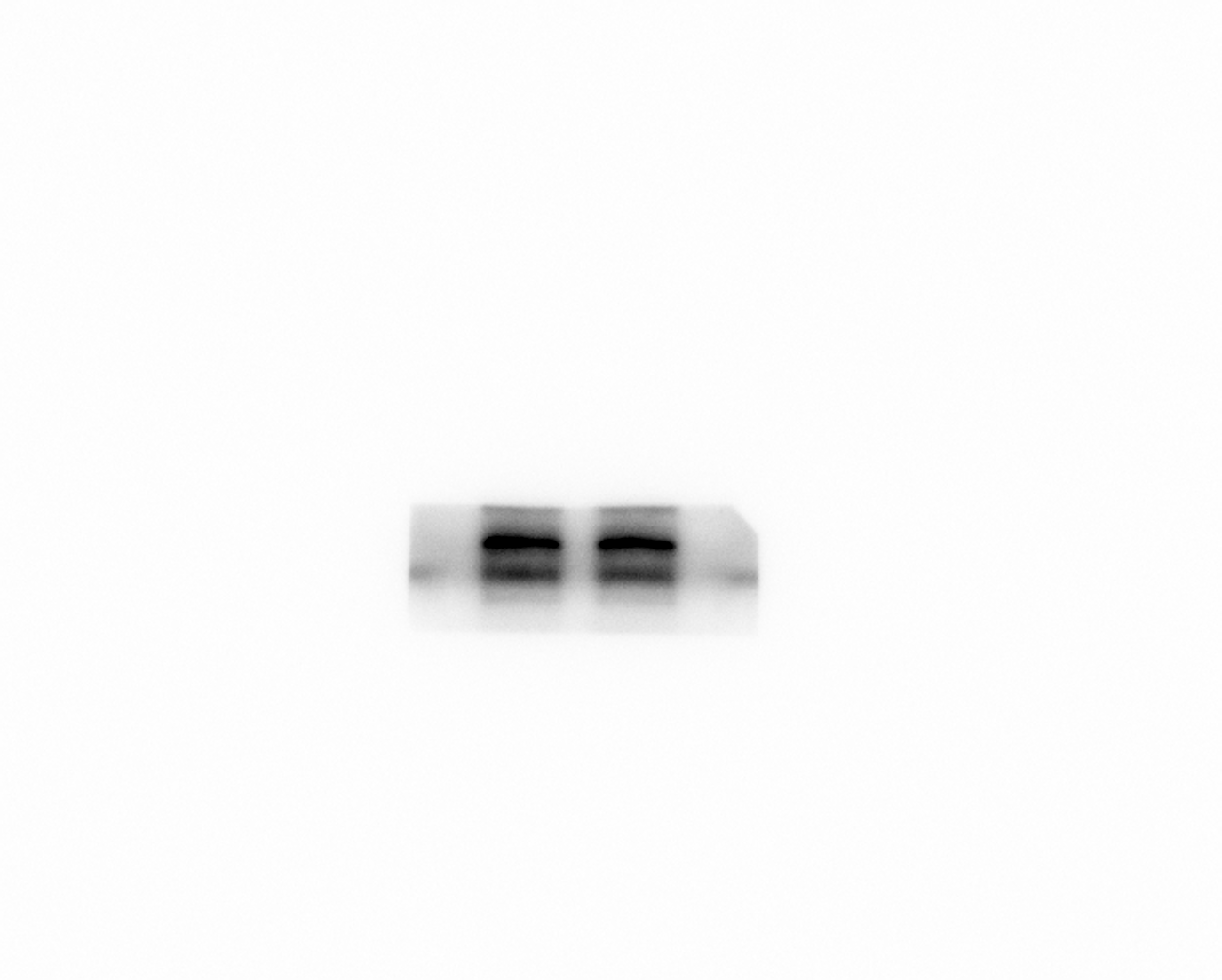

Supplement: Supplementary file 1 — Original western blots [file 41420_2025_2426_MOESM1_ESM.zip › fig3/c/fbp1-c.Tif]

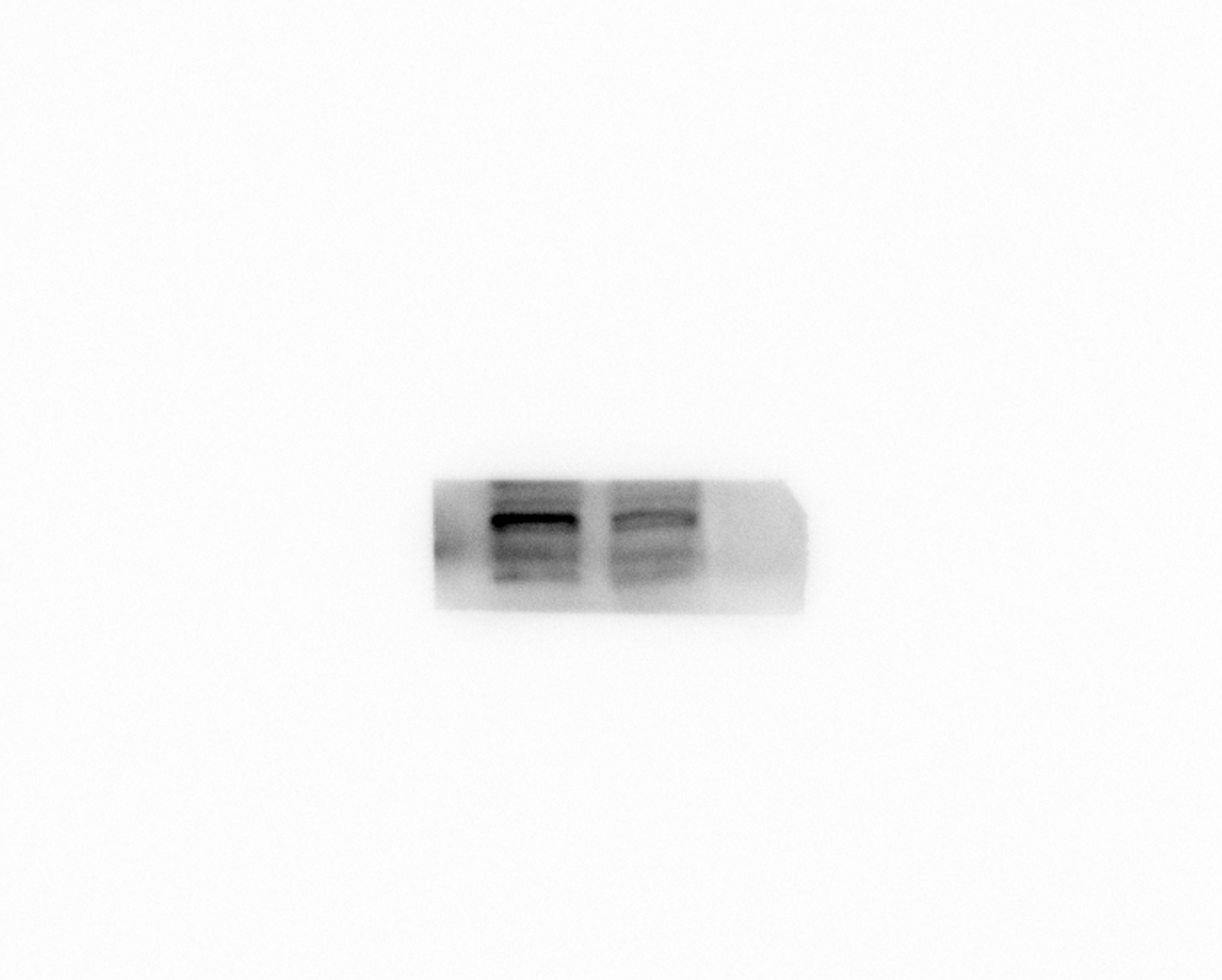

Supplement: Supplementary file 1 — Original western blots [file 41420_2025_2426_MOESM1_ESM.zip › fig3/c/fbp1-n.Tif]

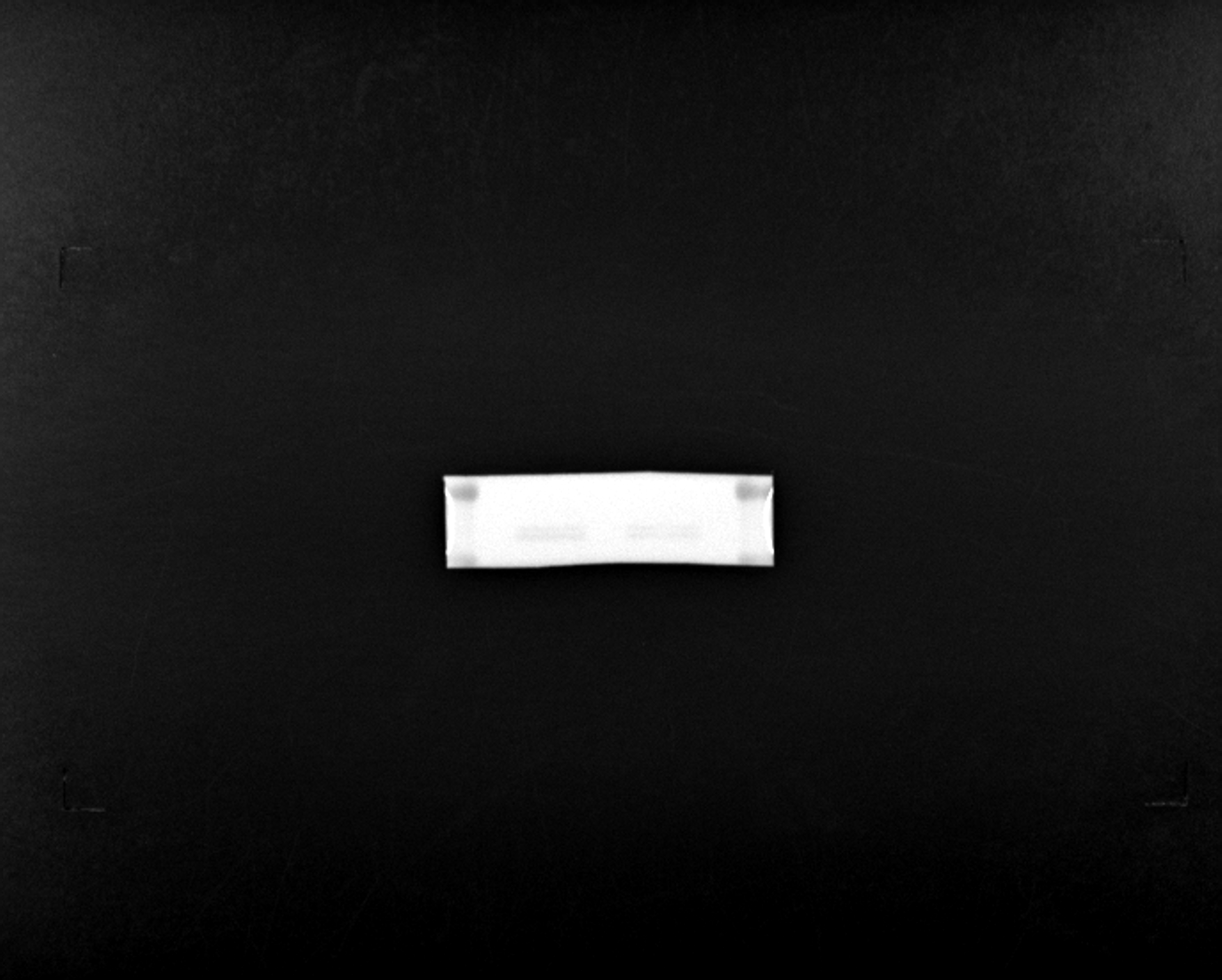

Supplement: Supplementary file 1 — Original western blots [file 41420_2025_2426_MOESM1_ESM.zip › fig3/c/lamin-c.Tif]

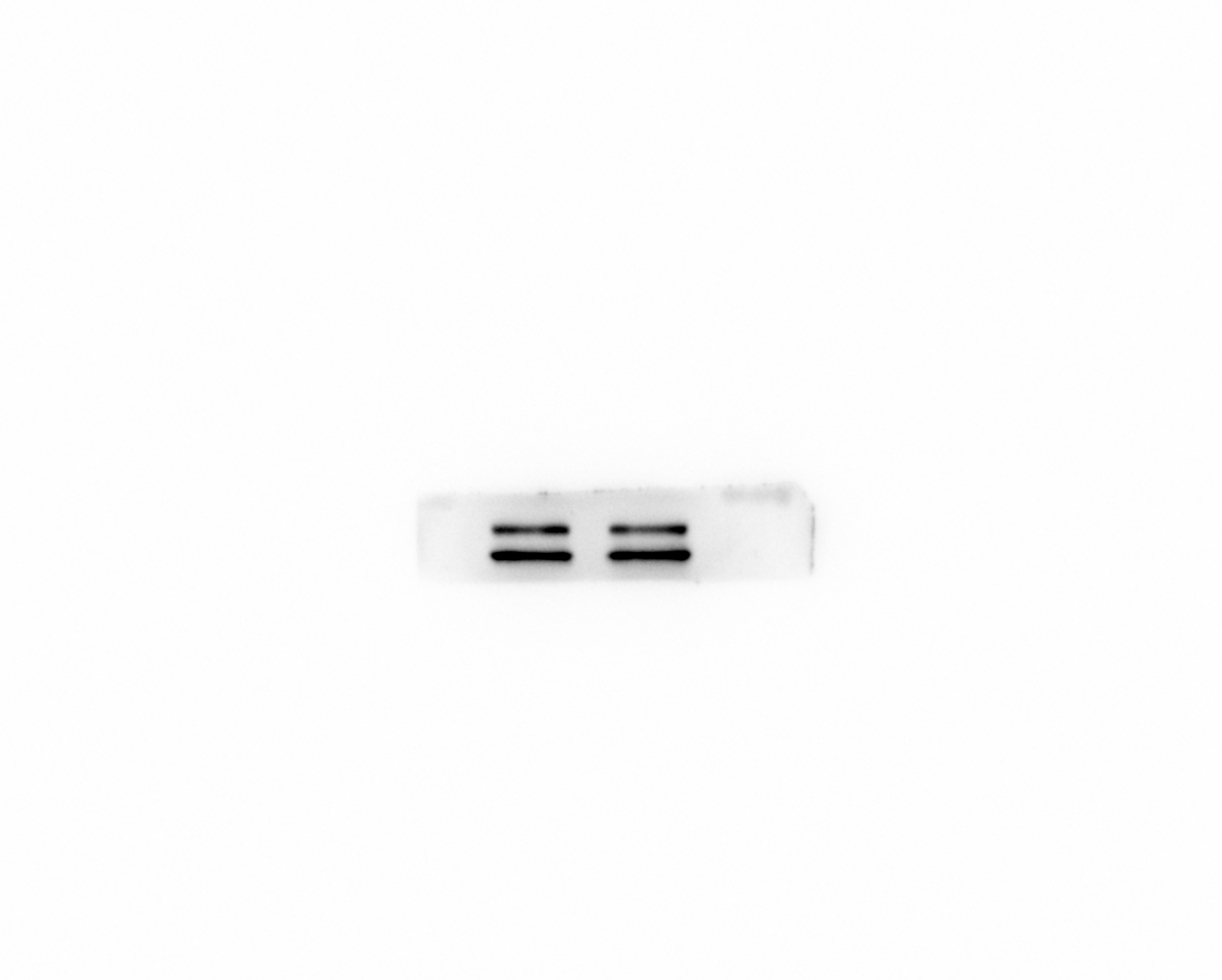

Supplement: Supplementary file 1 — Original western blots [file 41420_2025_2426_MOESM1_ESM.zip › fig3/c/lamin-n.Tif]

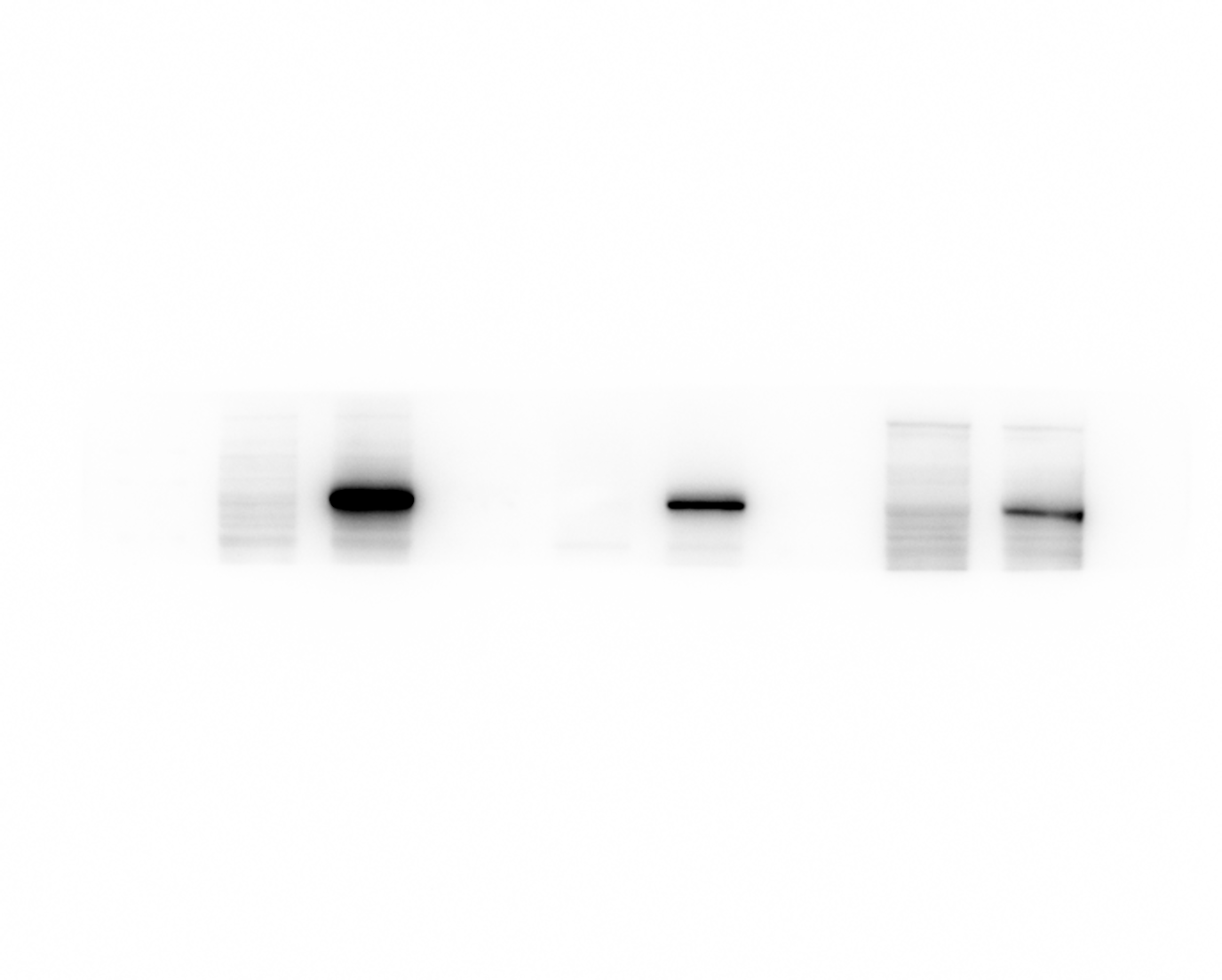

Supplement: Supplementary file 1 — Original western blots [file 41420_2025_2426_MOESM1_ESM.zip › fig3/c/myc.Tif]

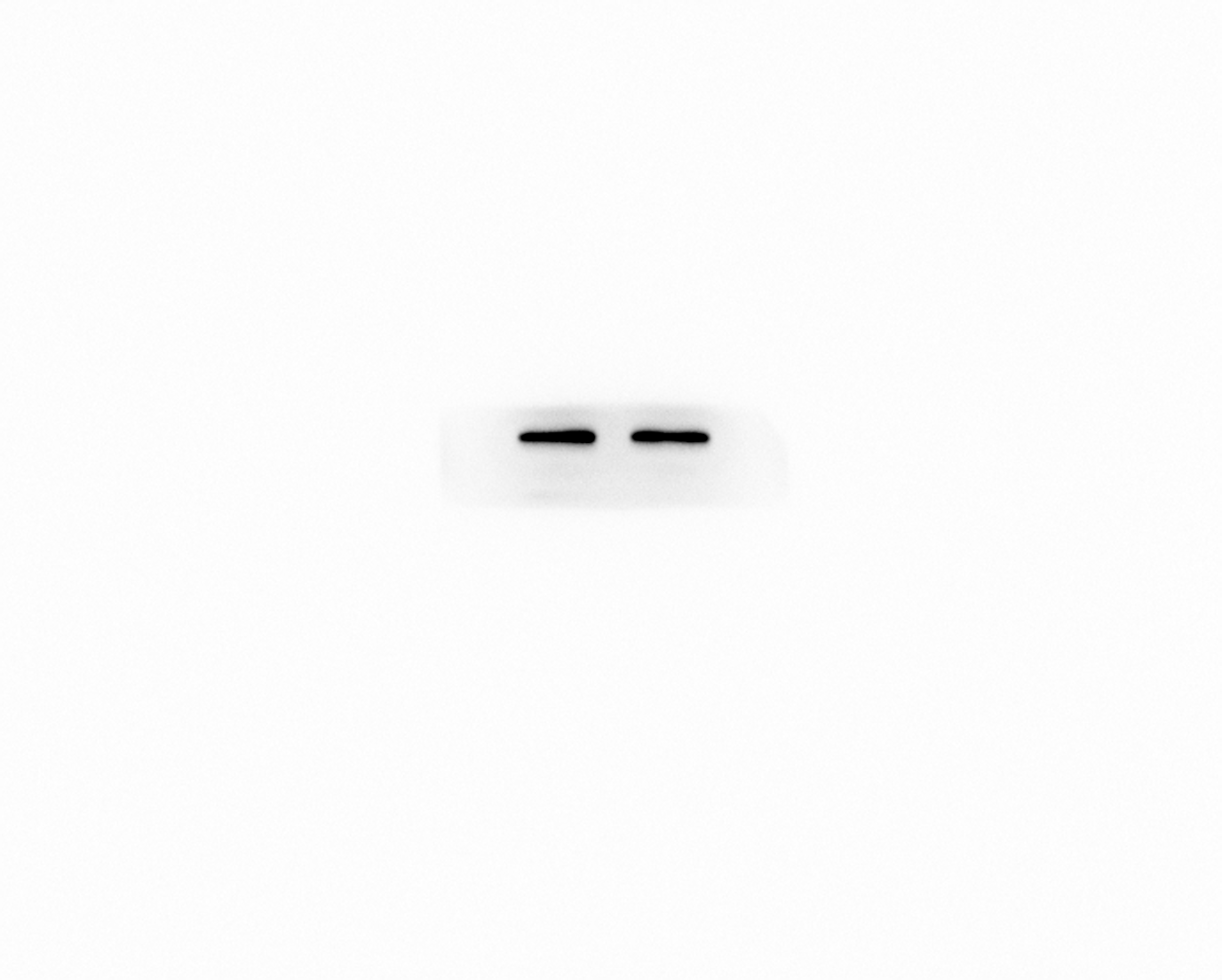

Supplement: Supplementary file 1 — Original western blots [file 41420_2025_2426_MOESM1_ESM.zip › fig3/c/tubulin-c.Tif]

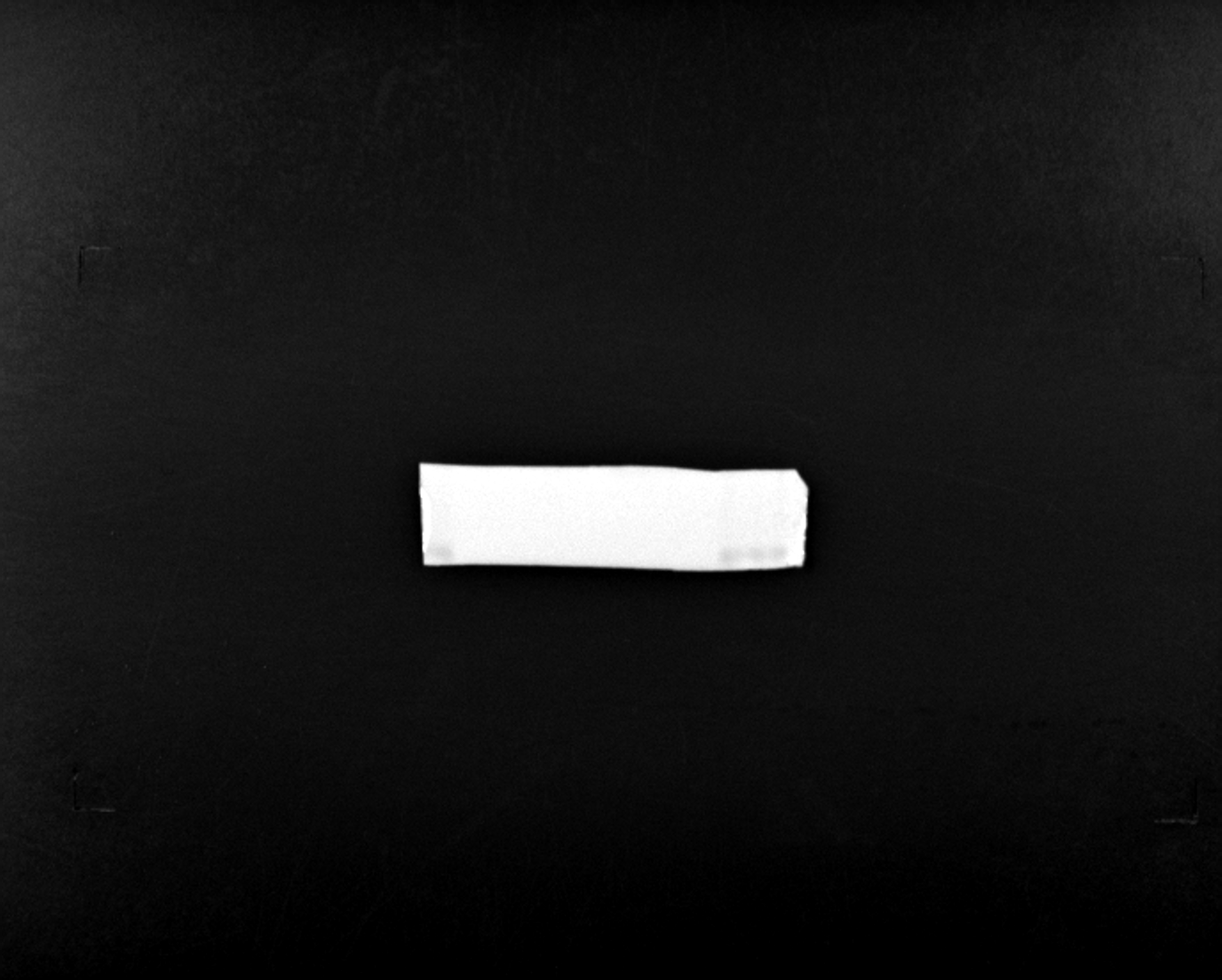

Supplement: Supplementary file 1 — Original western blots [file 41420_2025_2426_MOESM1_ESM.zip › fig3/c/tubulin-n.Tif]

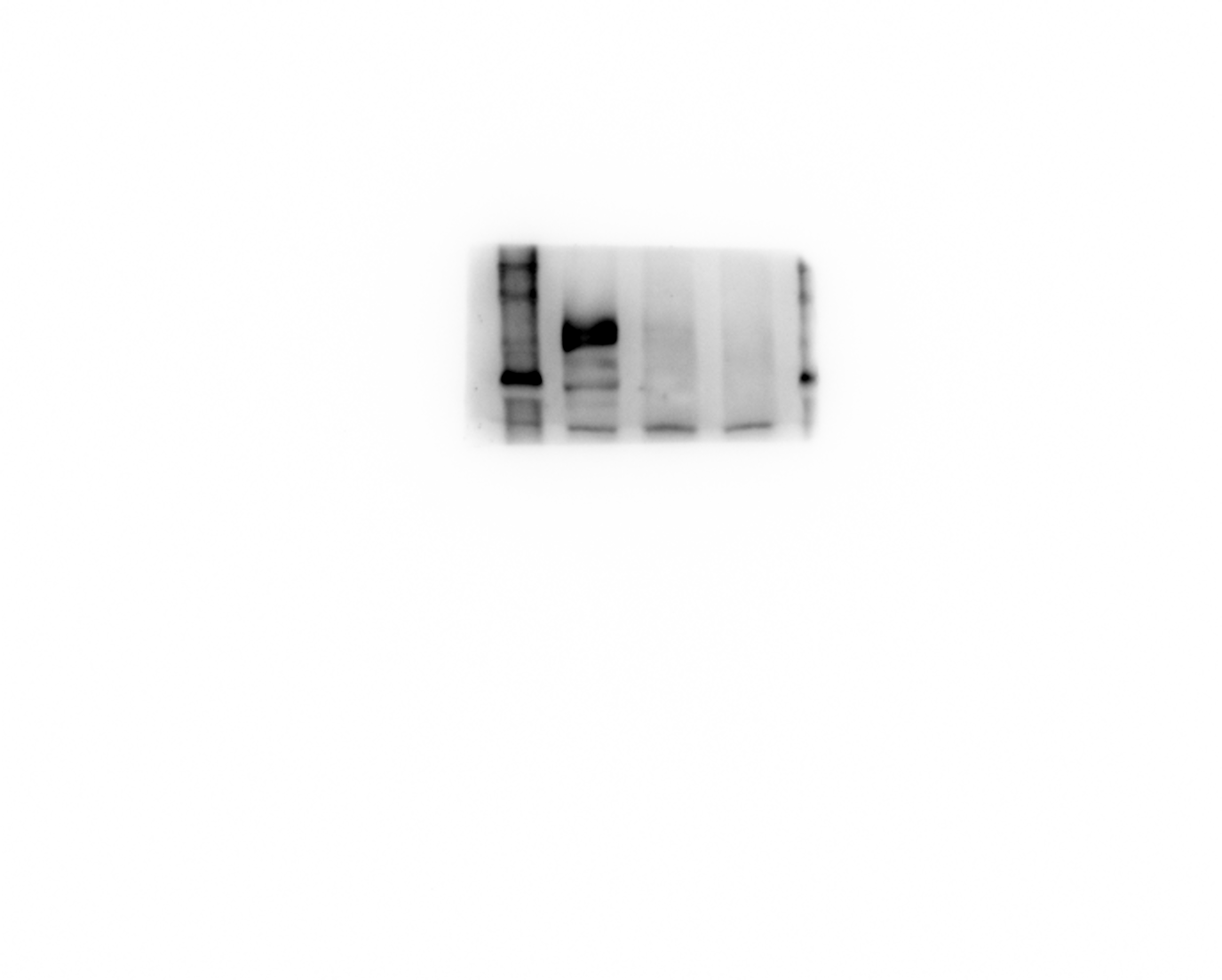

Supplement: Supplementary file 1 — Original western blots [file 41420_2025_2426_MOESM1_ESM.zip › fig3/d/cul4b.Tif]

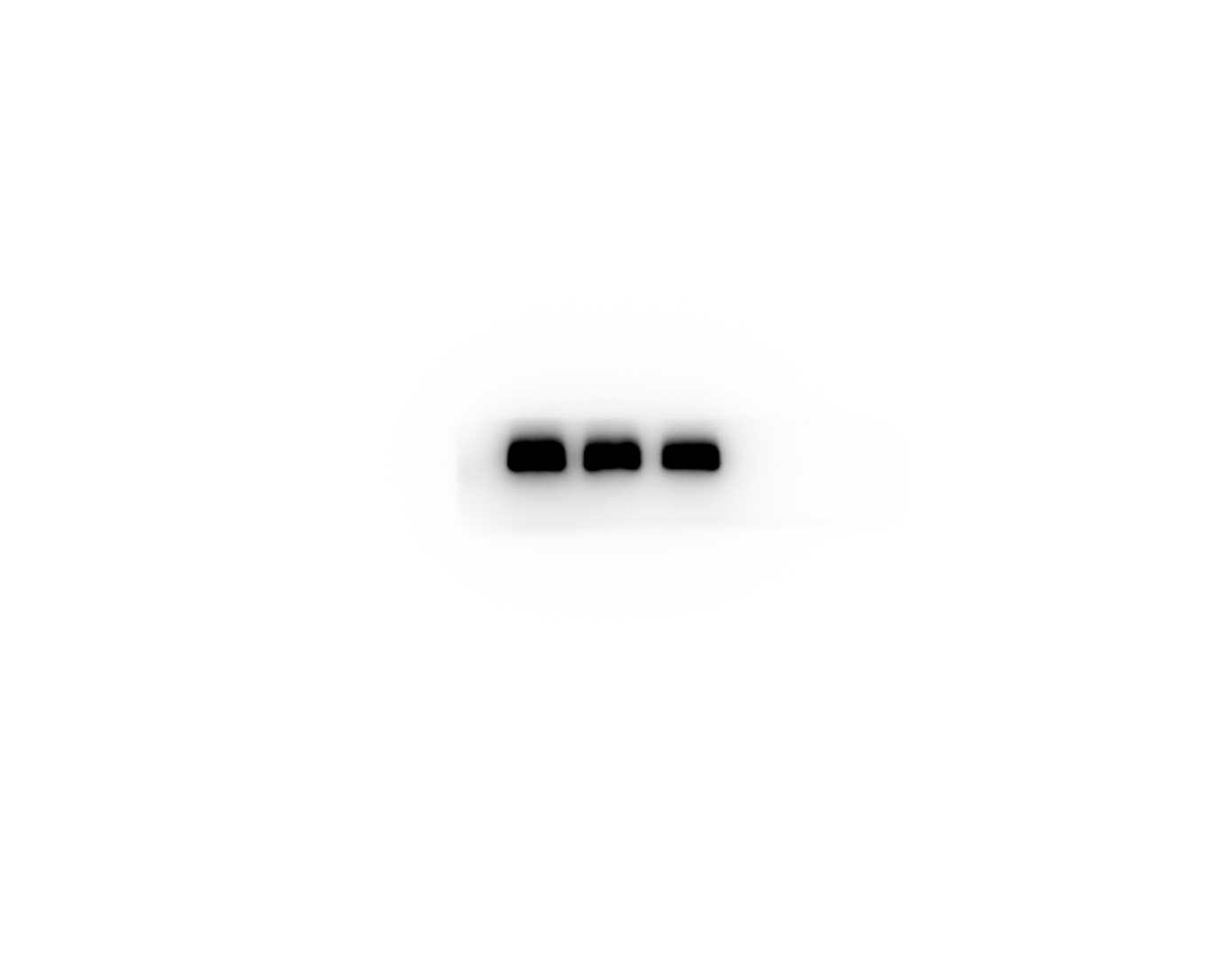

Supplement: Supplementary file 1 — Original western blots [file 41420_2025_2426_MOESM1_ESM.zip › fig3/d/tubulin.Tif]

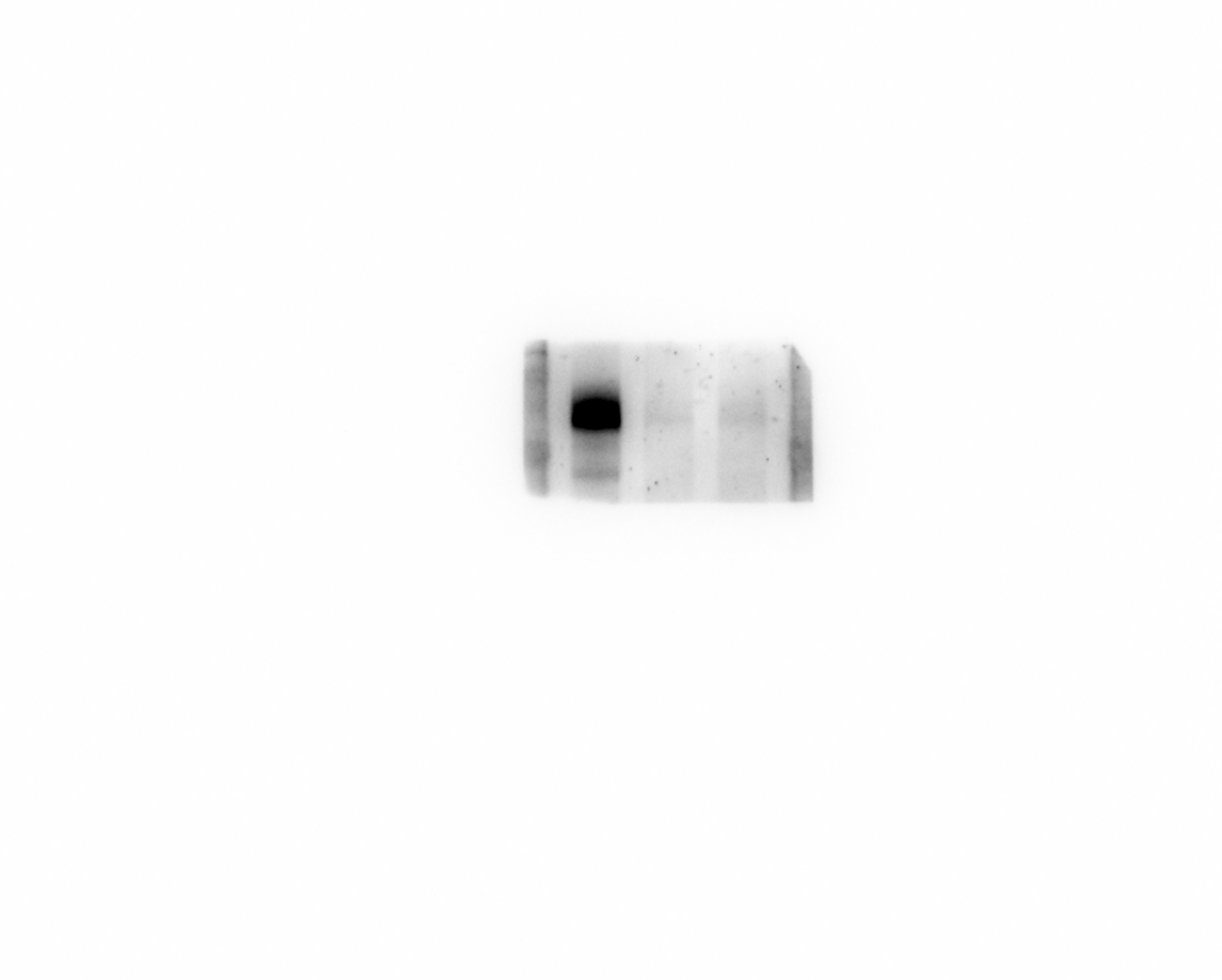

Supplement: Supplementary file 1 — Original western blots [file 41420_2025_2426_MOESM1_ESM.zip › fig3/e/cul4b-c.Tif]

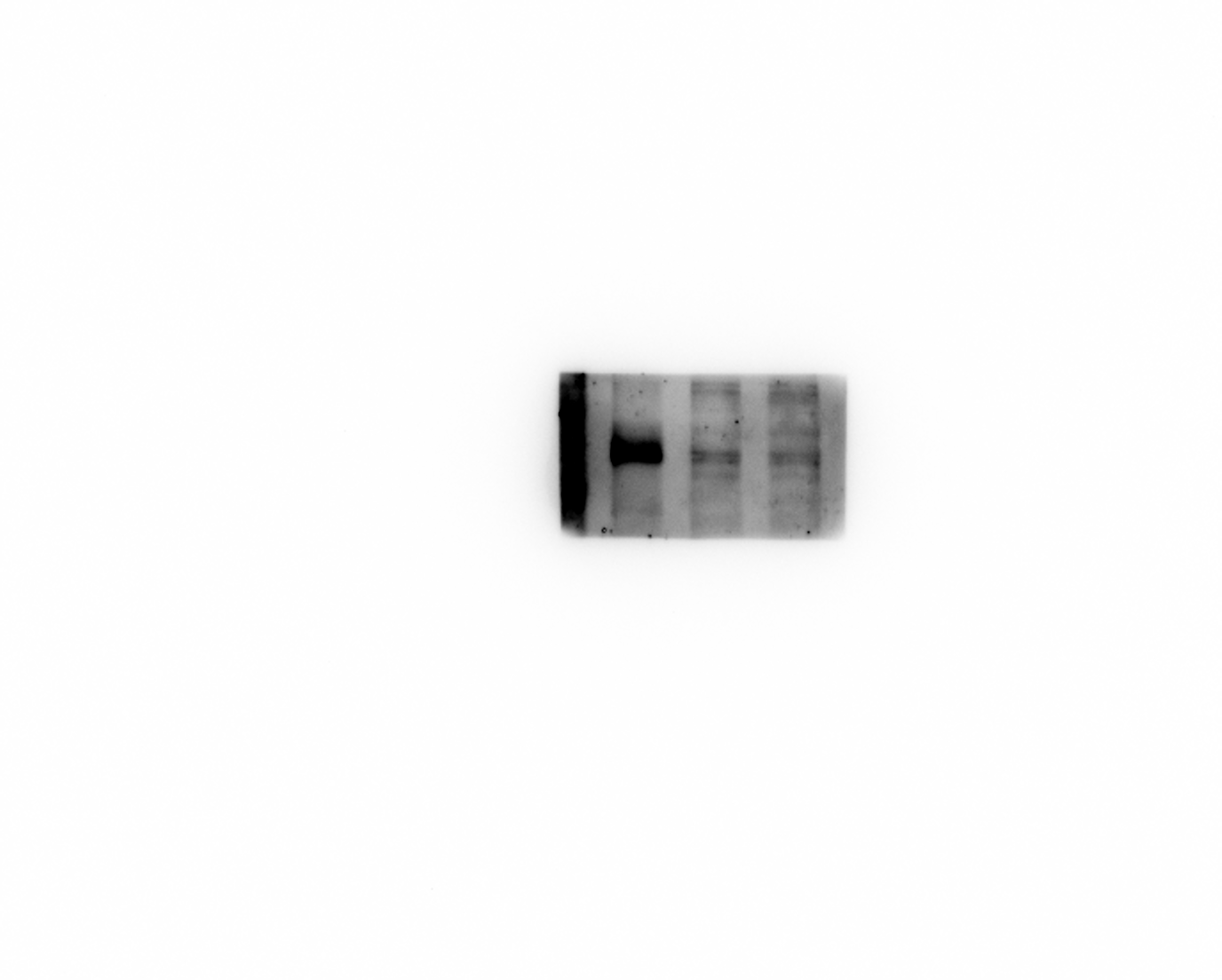

Supplement: Supplementary file 1 — Original western blots [file 41420_2025_2426_MOESM1_ESM.zip › fig3/e/cul4b-n.Tif]

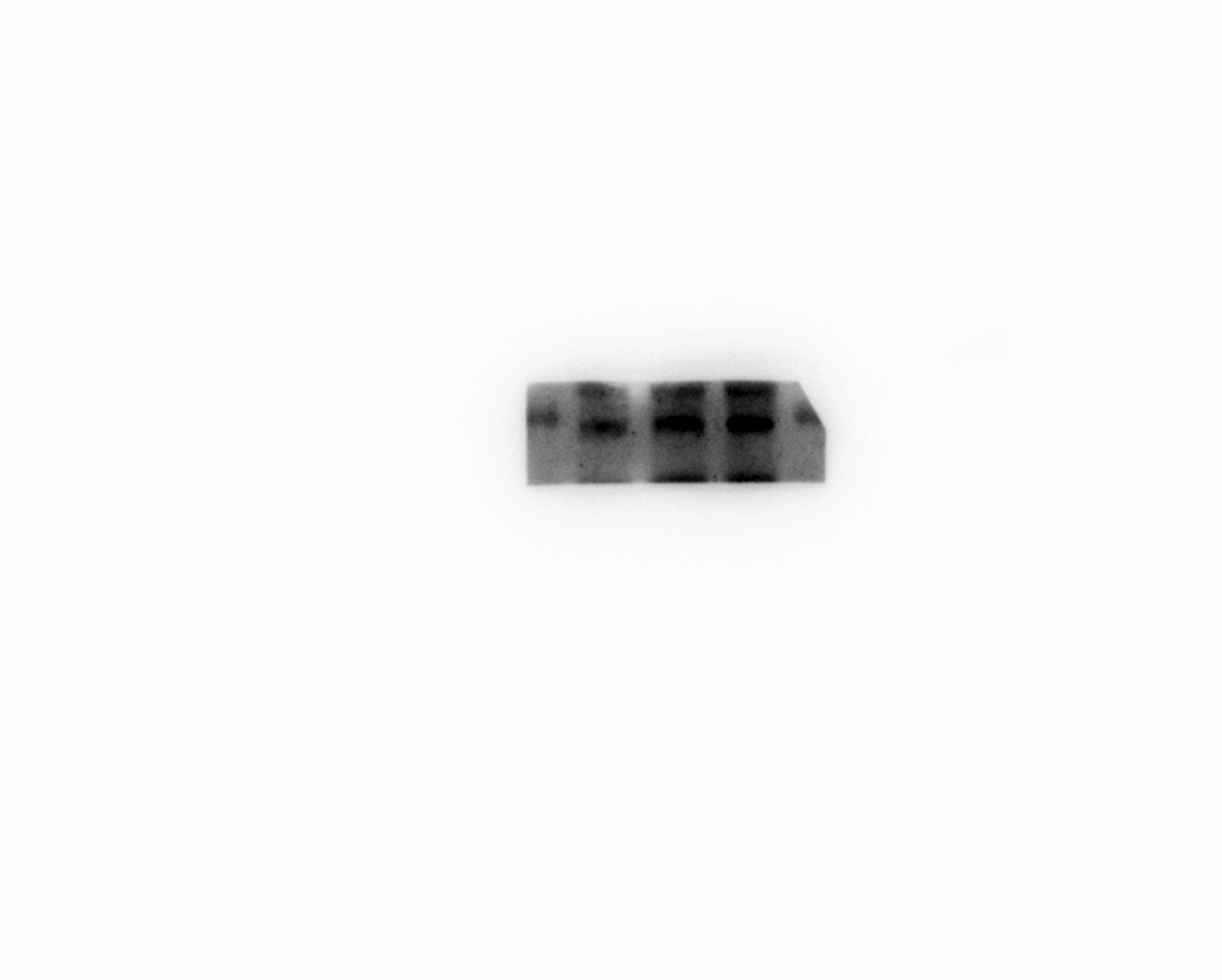

Supplement: Supplementary file 1 — Original western blots [file 41420_2025_2426_MOESM1_ESM.zip › fig3/e/fbp1-c.Tif]

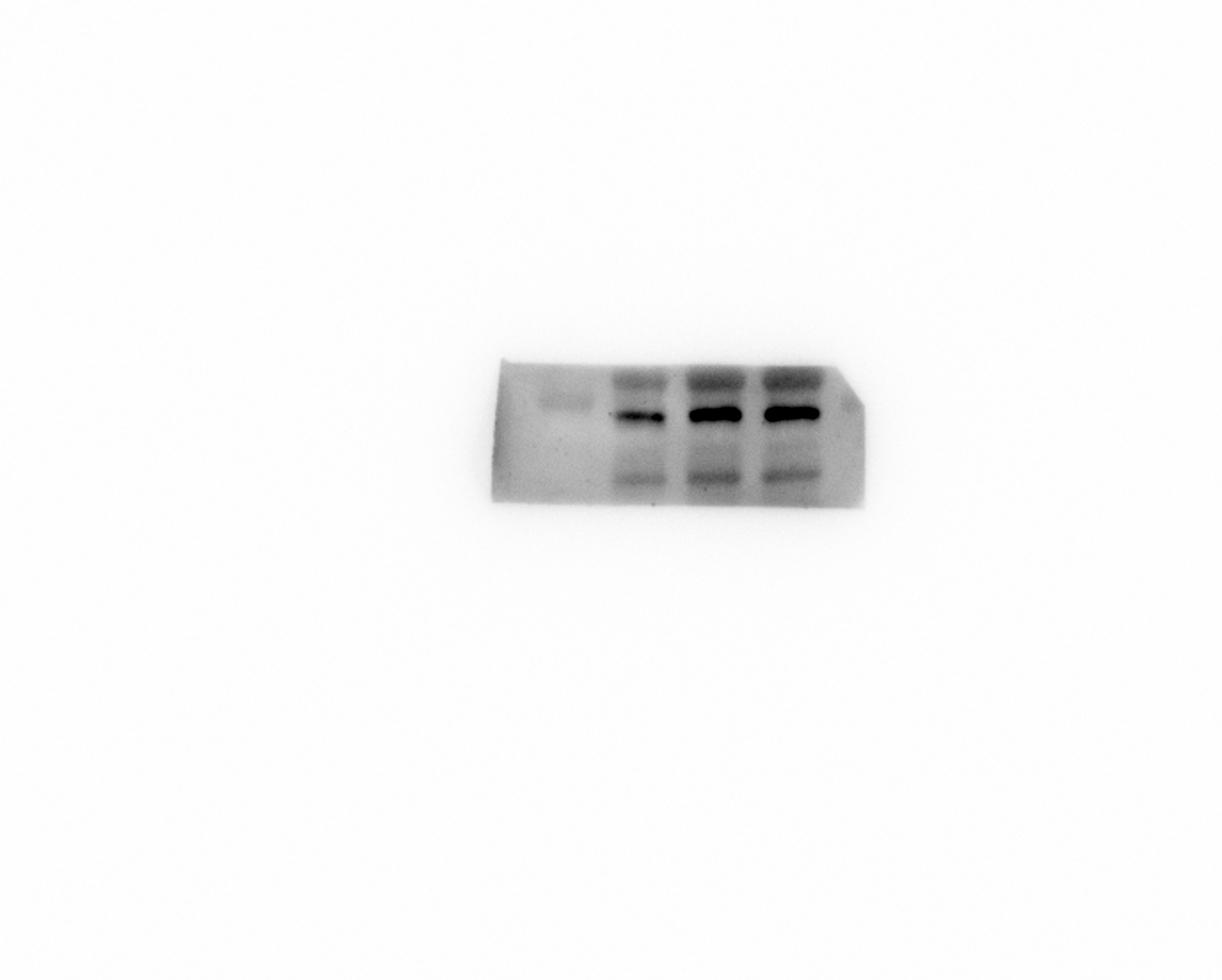

Supplement: Supplementary file 1 — Original western blots [file 41420_2025_2426_MOESM1_ESM.zip › fig3/e/fbp1-n.Tif]

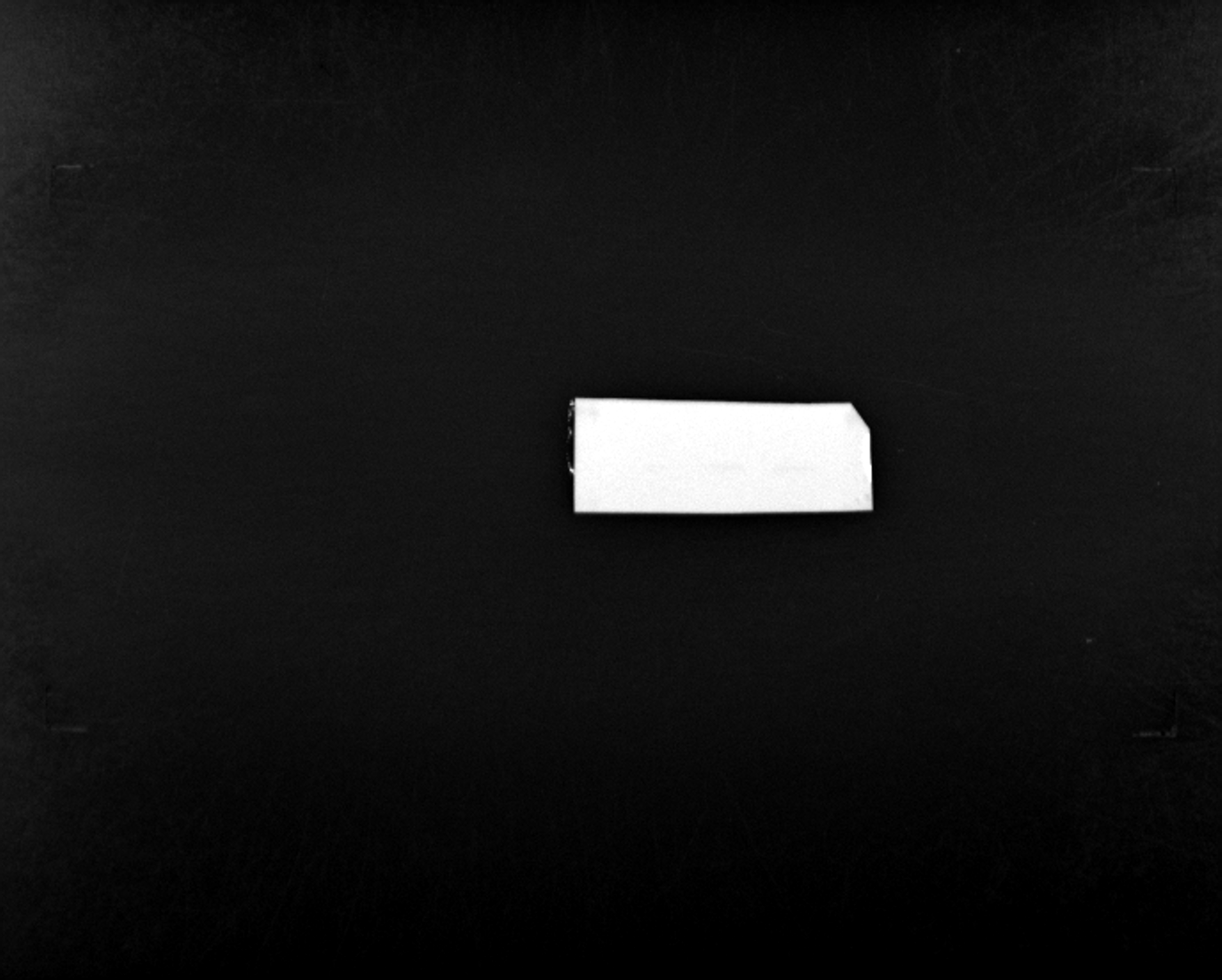

Supplement: Supplementary file 1 — Original western blots [file 41420_2025_2426_MOESM1_ESM.zip › fig3/e/lamin-c.Tif]

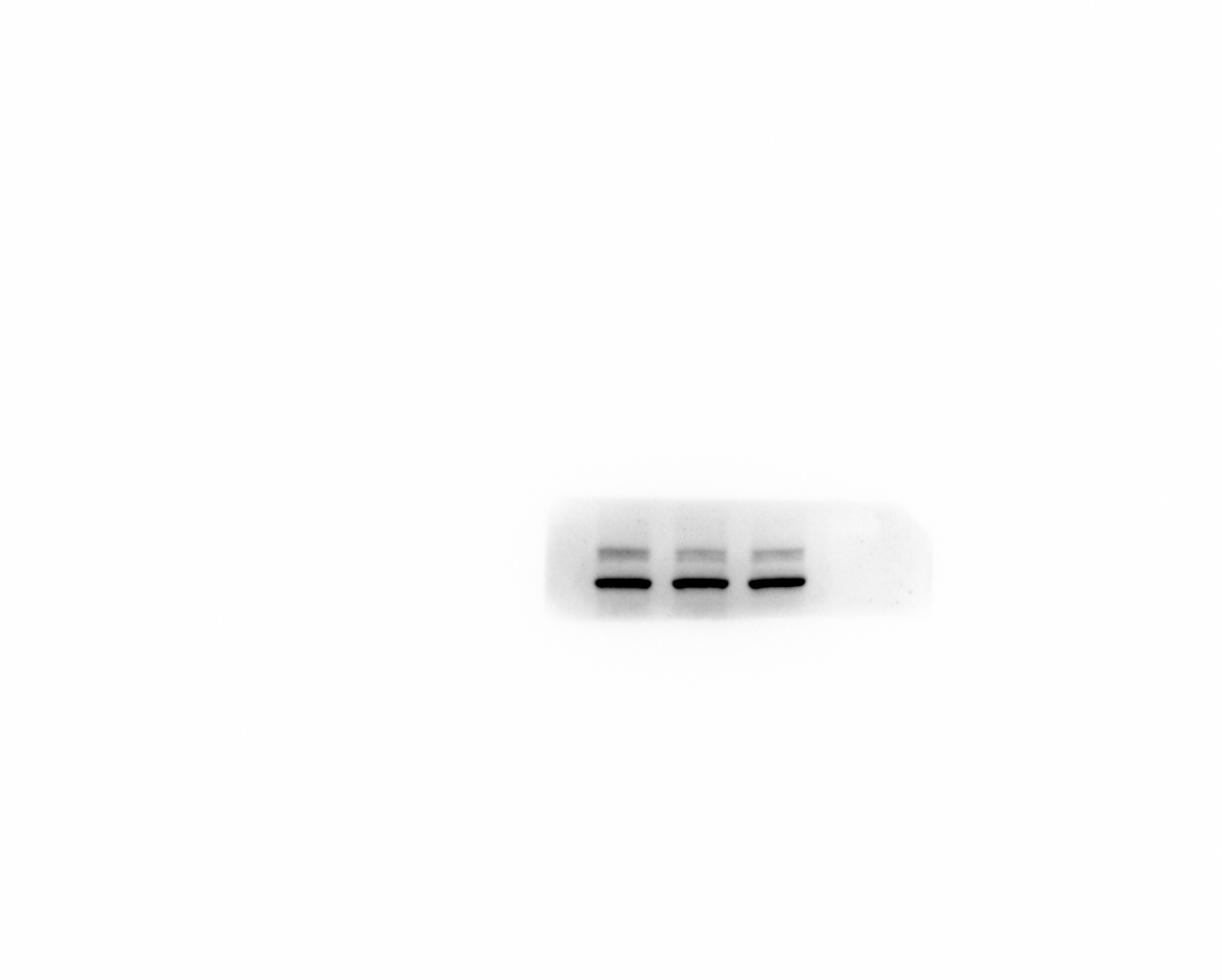

Supplement: Supplementary file 1 — Original western blots [file 41420_2025_2426_MOESM1_ESM.zip › fig3/e/lamin-n.Tif]

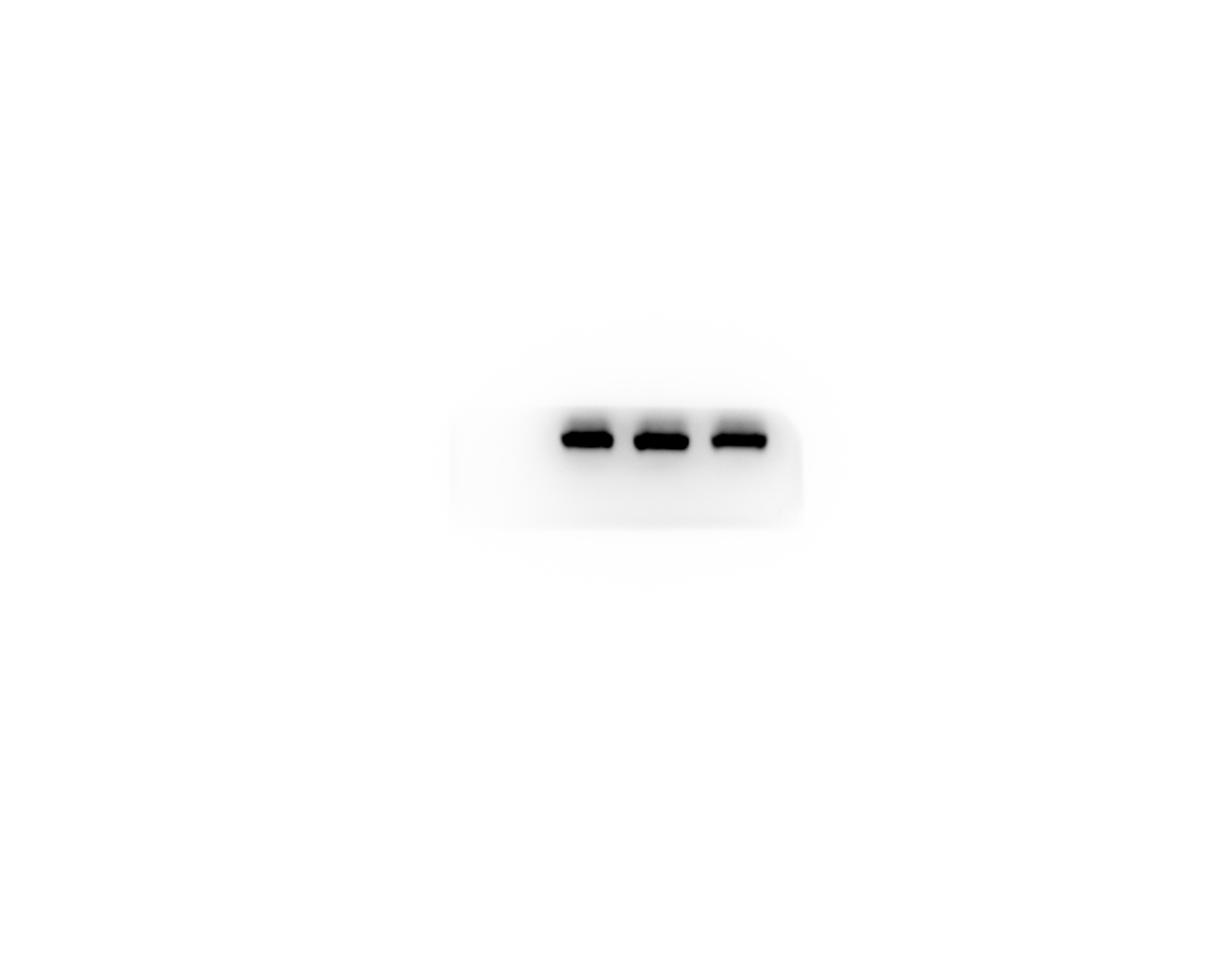

Supplement: Supplementary file 1 — Original western blots [file 41420_2025_2426_MOESM1_ESM.zip › fig3/e/tubulin-c.Tif]

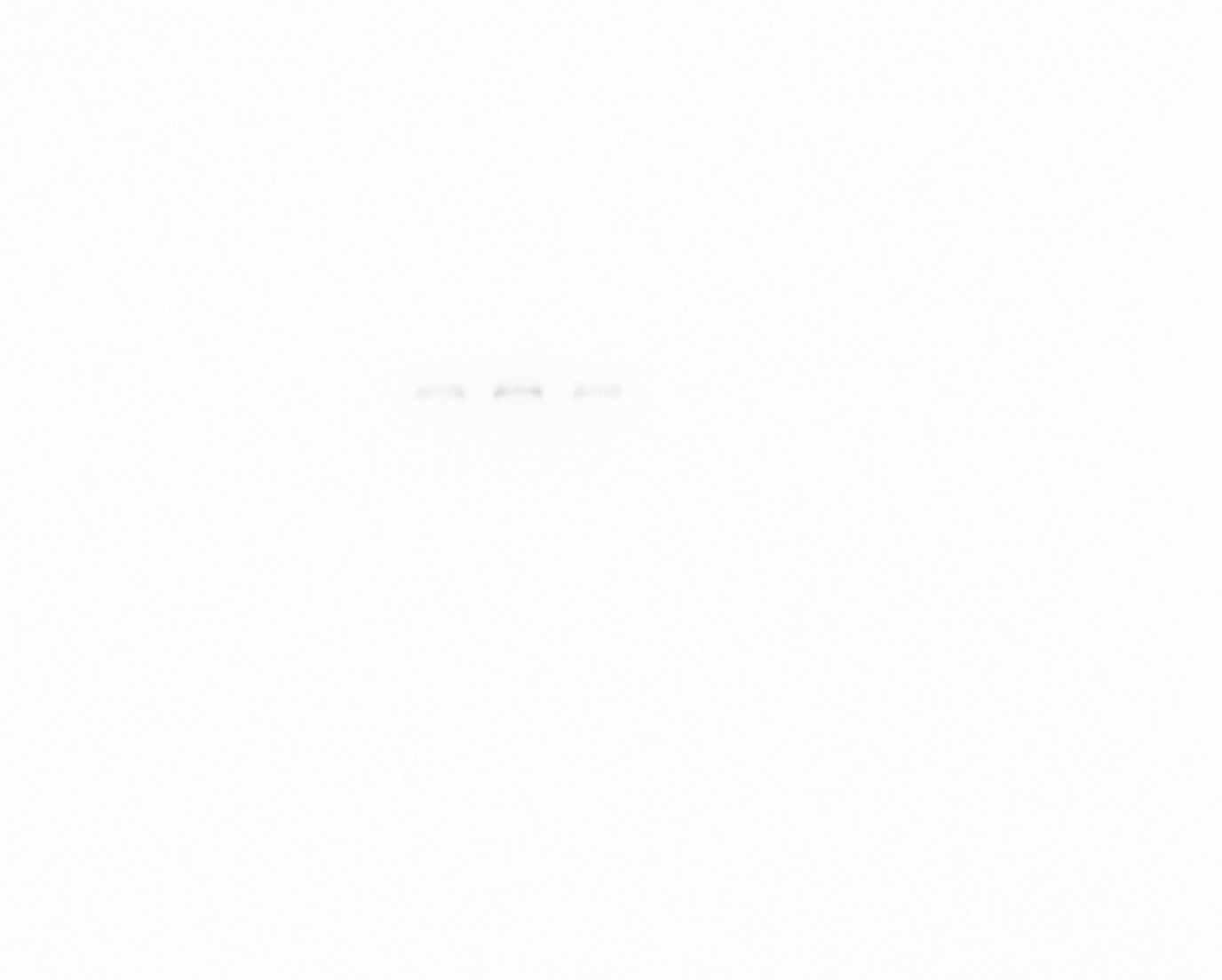

Supplement: Supplementary file 1 — Original western blots [file 41420_2025_2426_MOESM1_ESM.zip › fig3/e/tubulin-n.Tif]

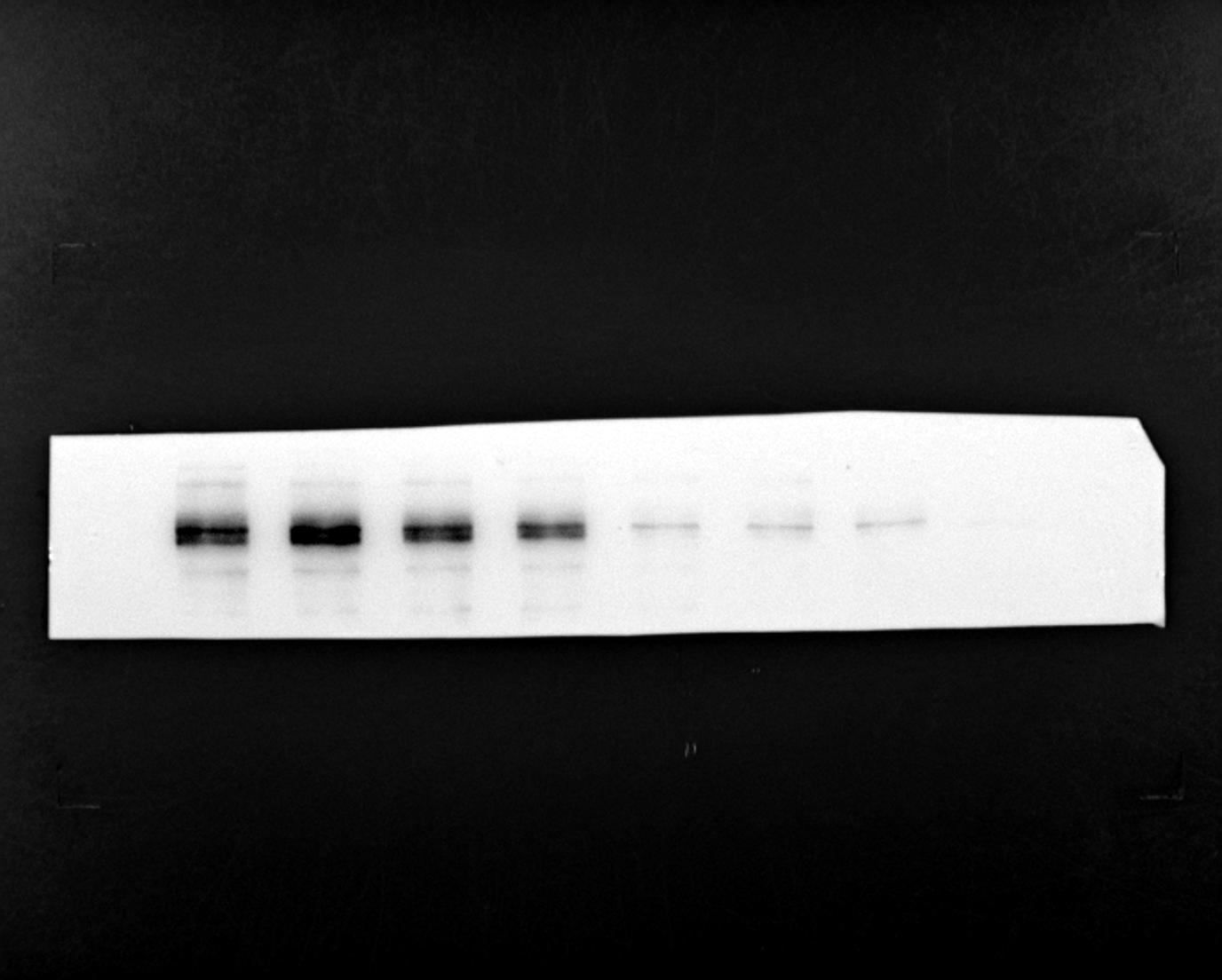

Supplement: Supplementary file 1 — Original western blots [file 41420_2025_2426_MOESM1_ESM.zip › fig3/g/cul4b.Tif]

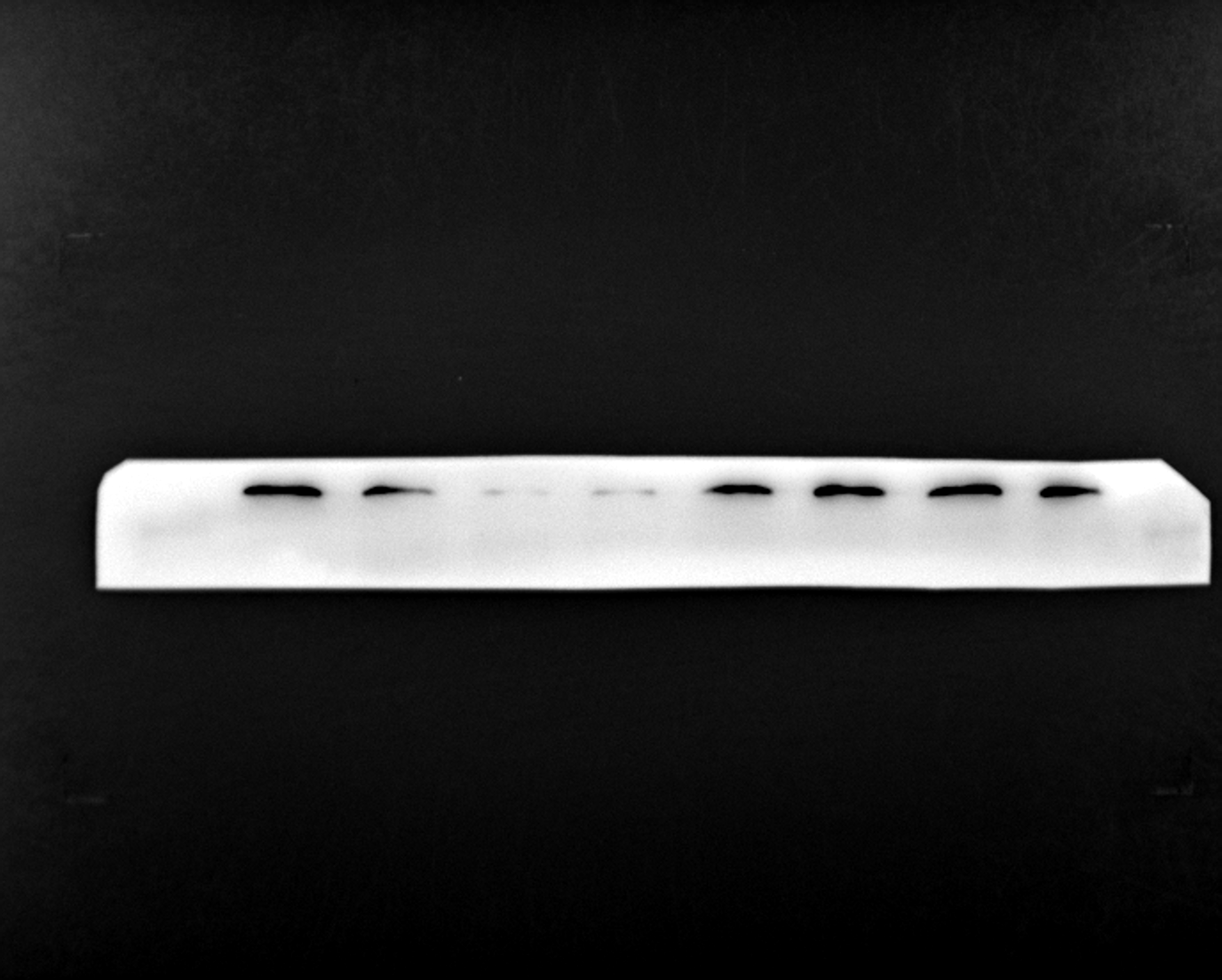

Supplement: Supplementary file 1 — Original western blots [file 41420_2025_2426_MOESM1_ESM.zip › fig3/g/fbp1.Tif]

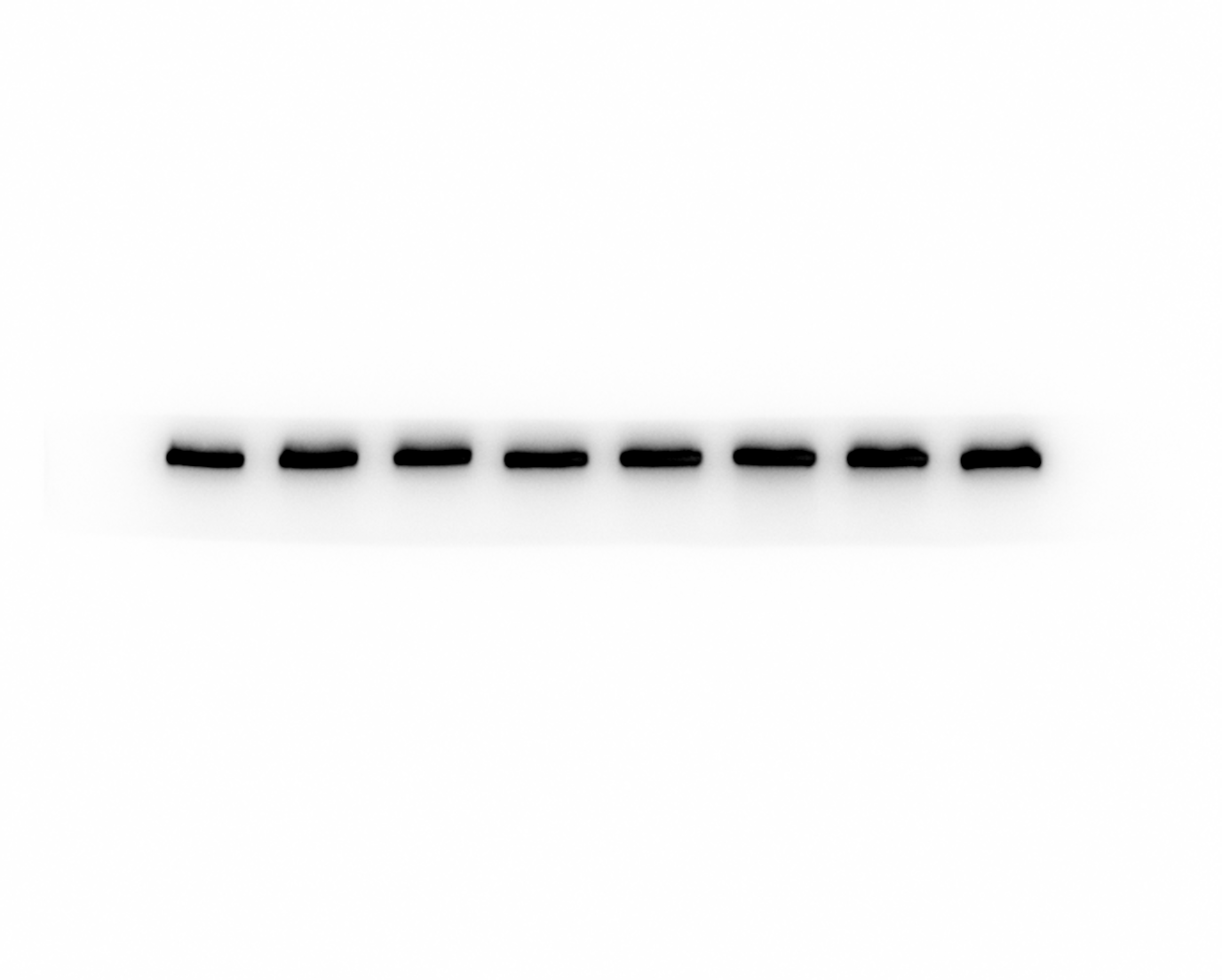

Supplement: Supplementary file 1 — Original western blots [file 41420_2025_2426_MOESM1_ESM.zip › fig3/g/tubulin.Tif]

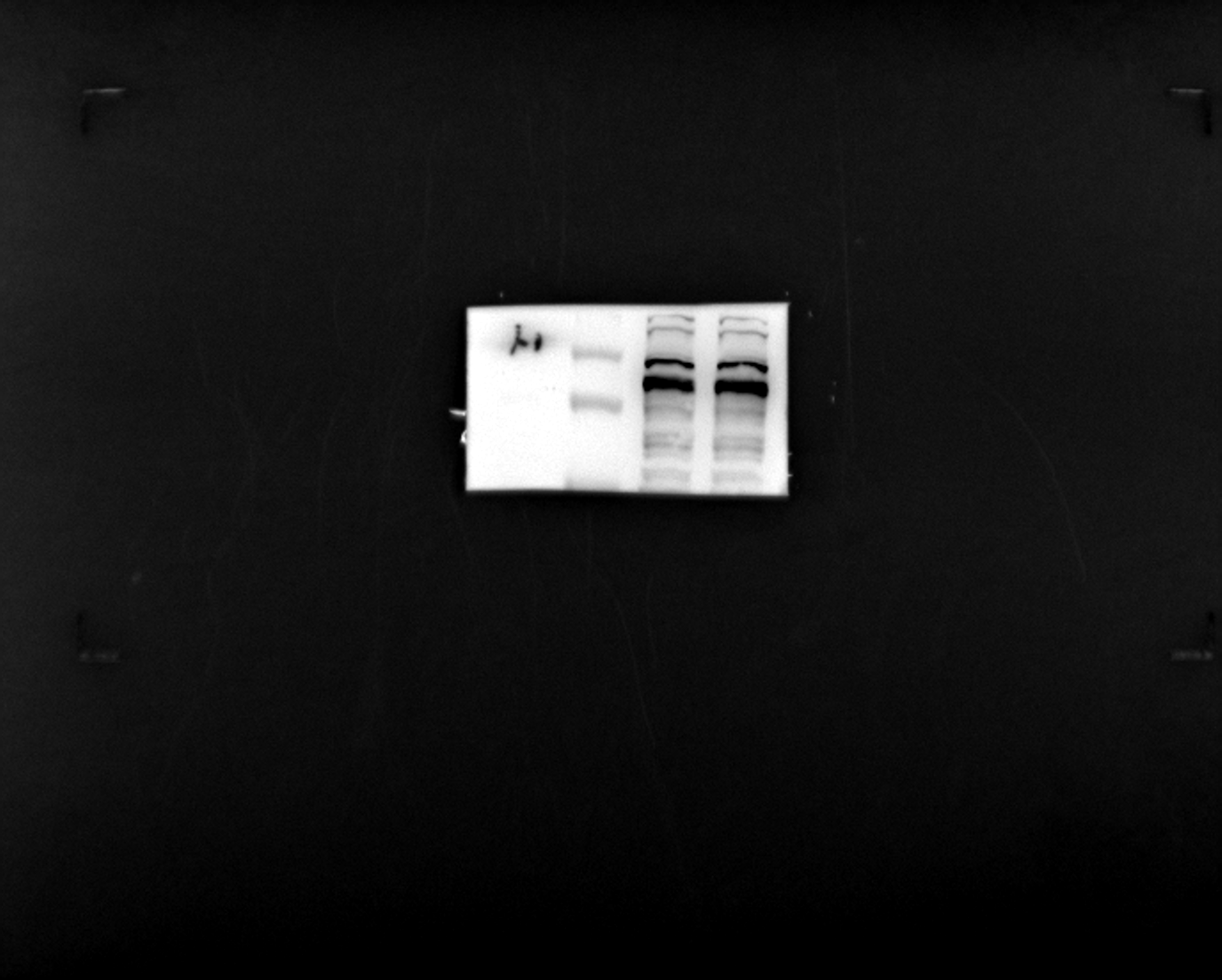

Supplement: Supplementary file 1 — Original western blots [file 41420_2025_2426_MOESM1_ESM.zip › fig4/a/HIF-2A'.Tif]

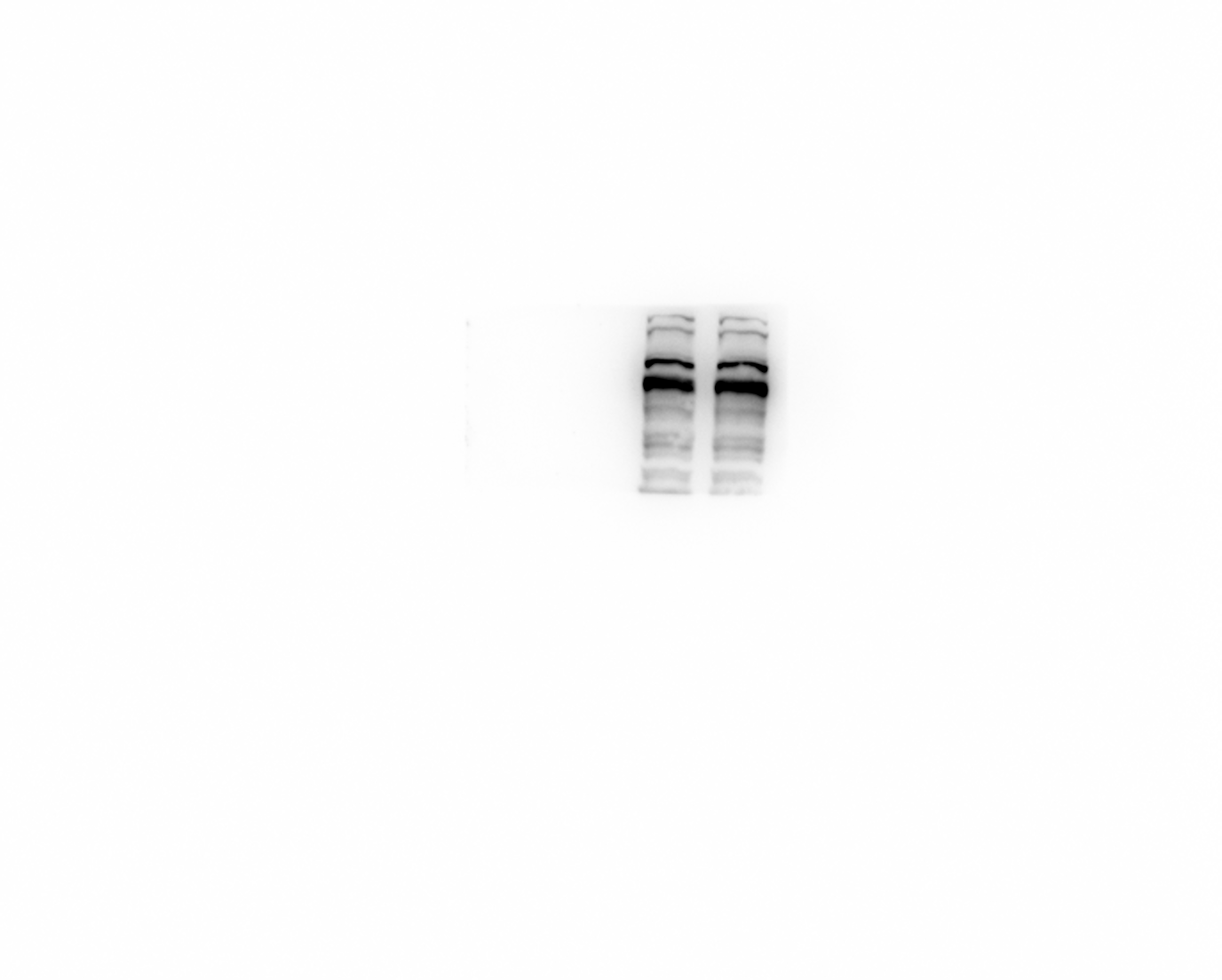

Supplement: Supplementary file 1 — Original western blots [file 41420_2025_2426_MOESM1_ESM.zip › fig4/a/HIF-2A.Tif]

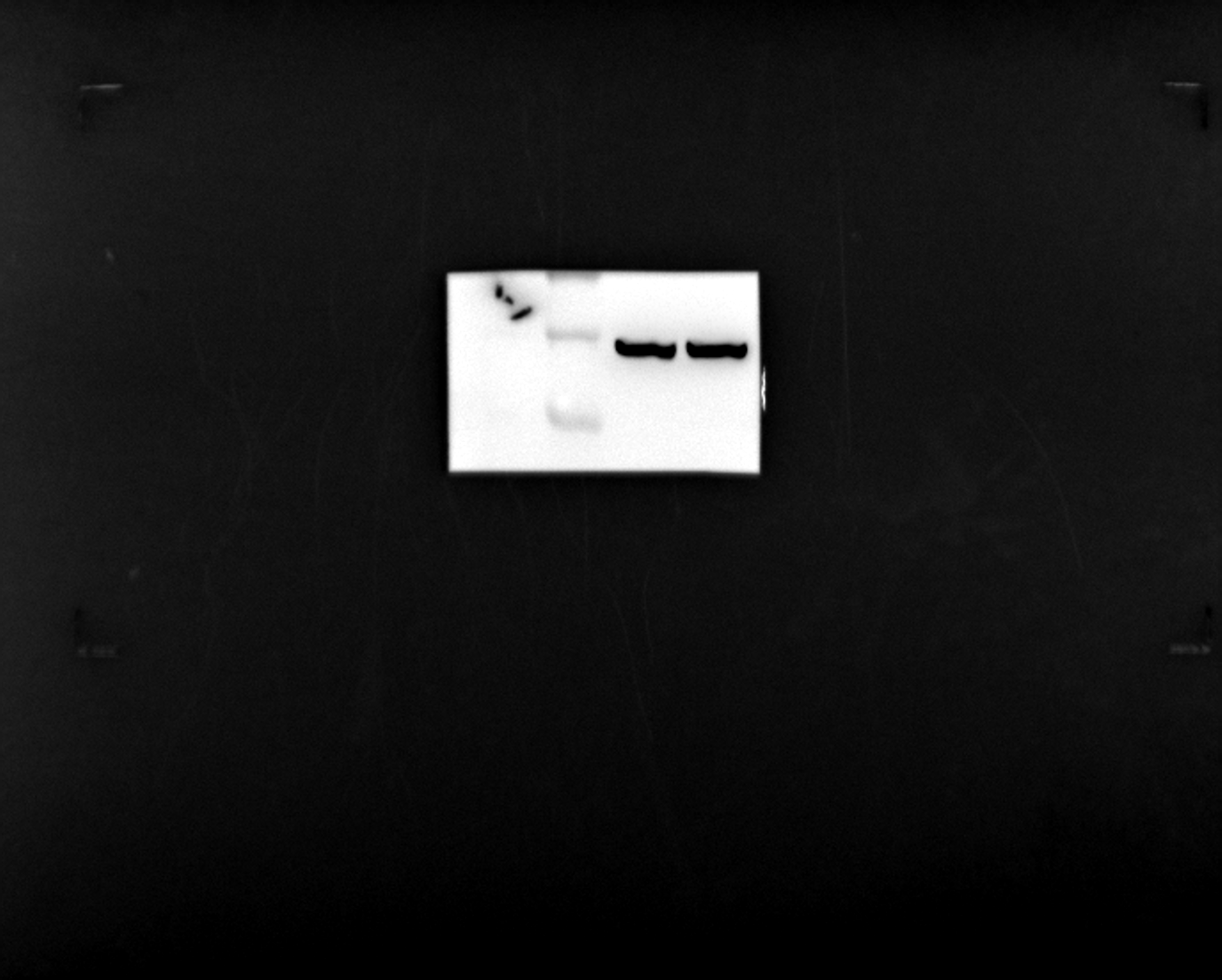

Supplement: Supplementary file 1 — Original western blots [file 41420_2025_2426_MOESM1_ESM.zip › fig4/a/TUBULIN'.Tif]

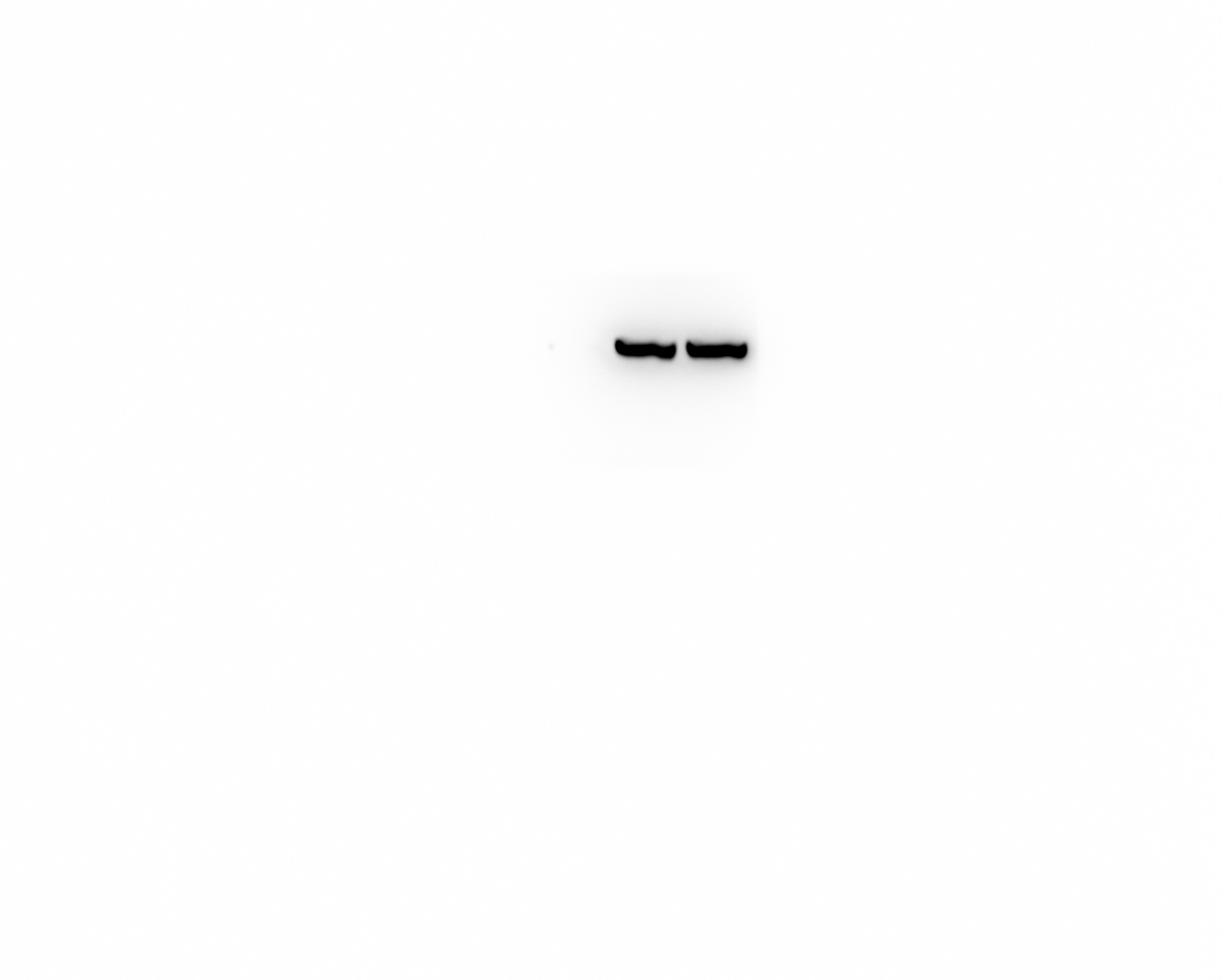

Supplement: Supplementary file 1 — Original western blots [file 41420_2025_2426_MOESM1_ESM.zip › fig4/a/TUBULIN.Tif]

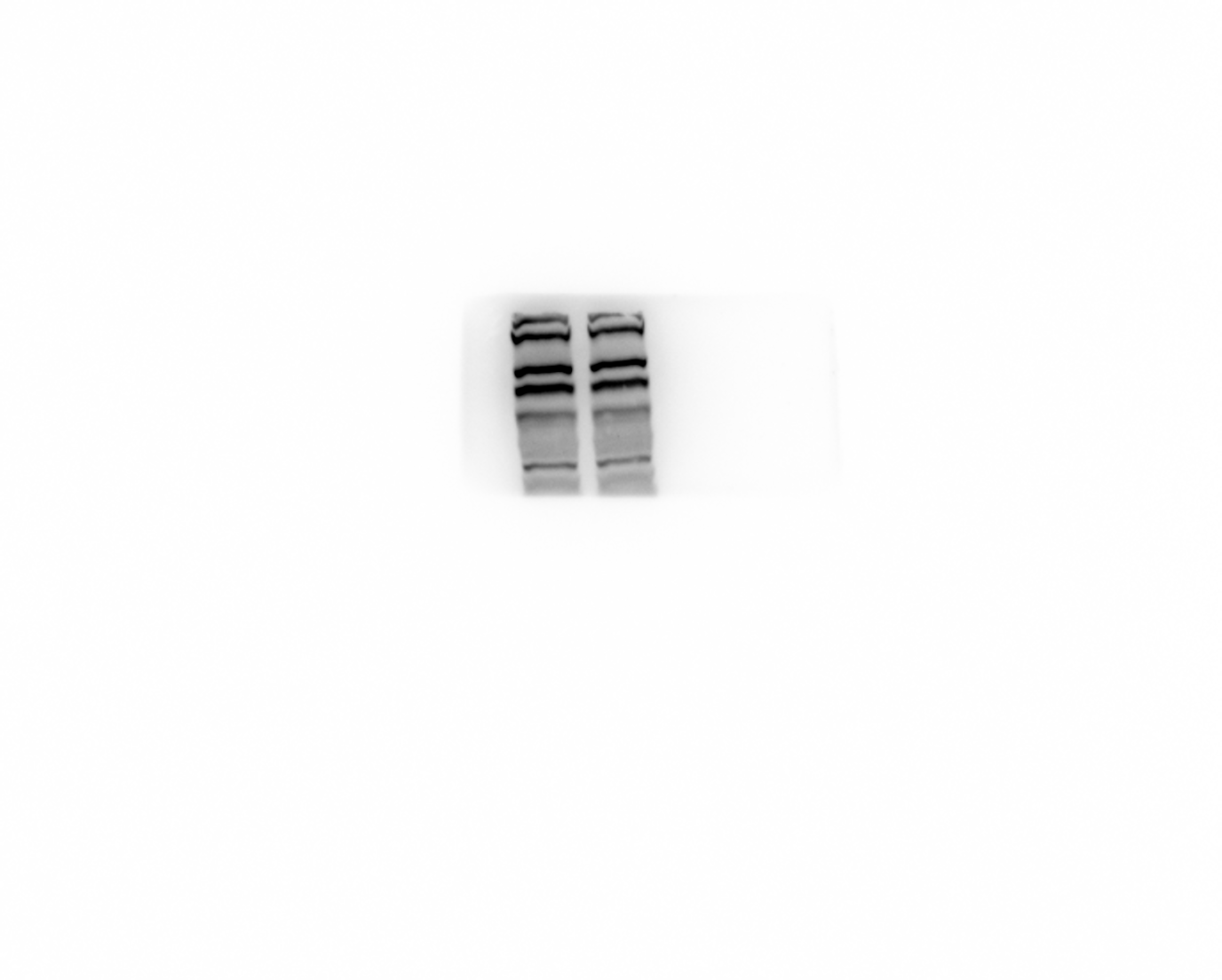

Supplement: Supplementary file 1 — Original western blots [file 41420_2025_2426_MOESM1_ESM.zip › fig4/b/HIF-2A'.Tif]

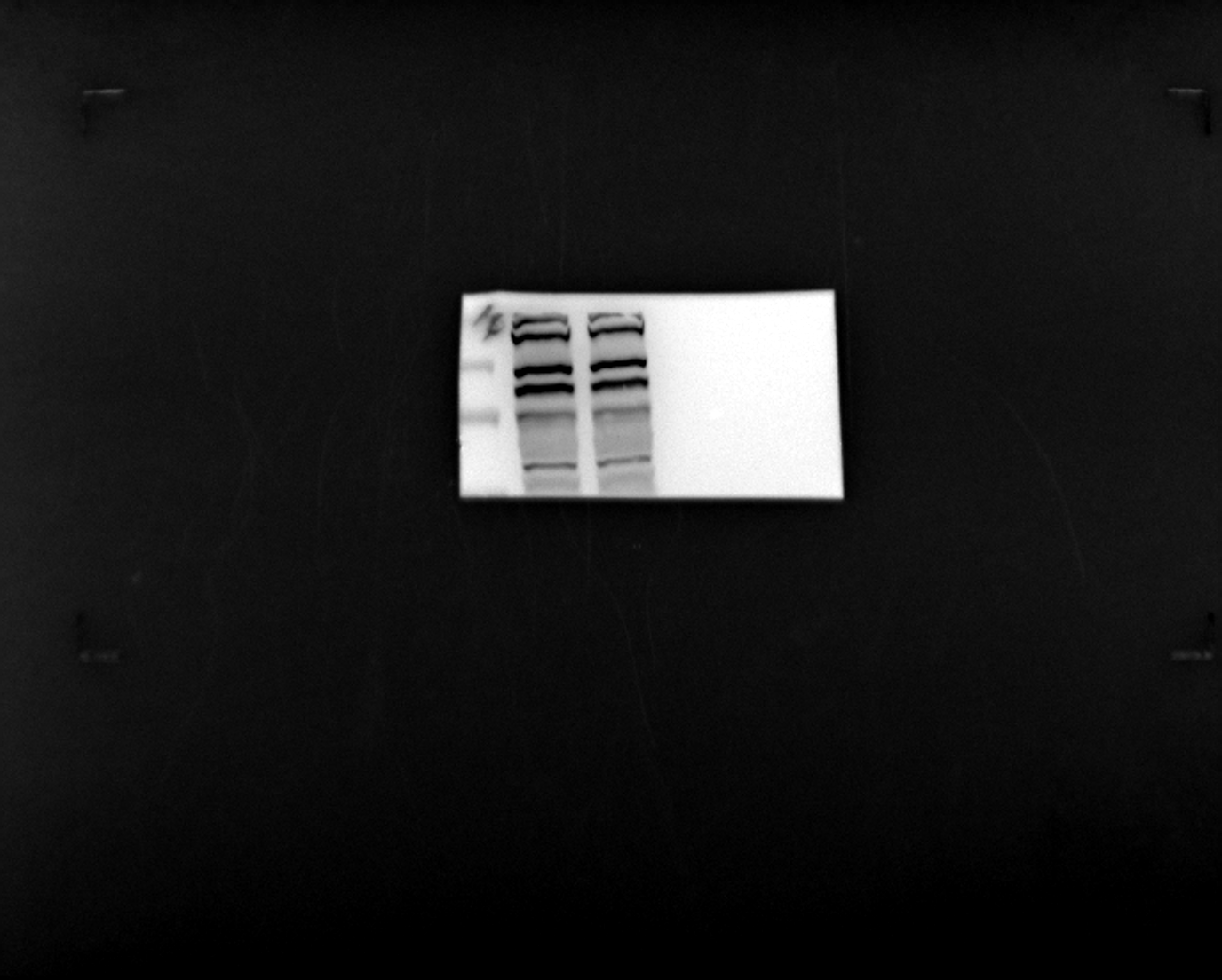

Supplement: Supplementary file 1 — Original western blots [file 41420_2025_2426_MOESM1_ESM.zip › fig4/b/HIF-2A.Tif]

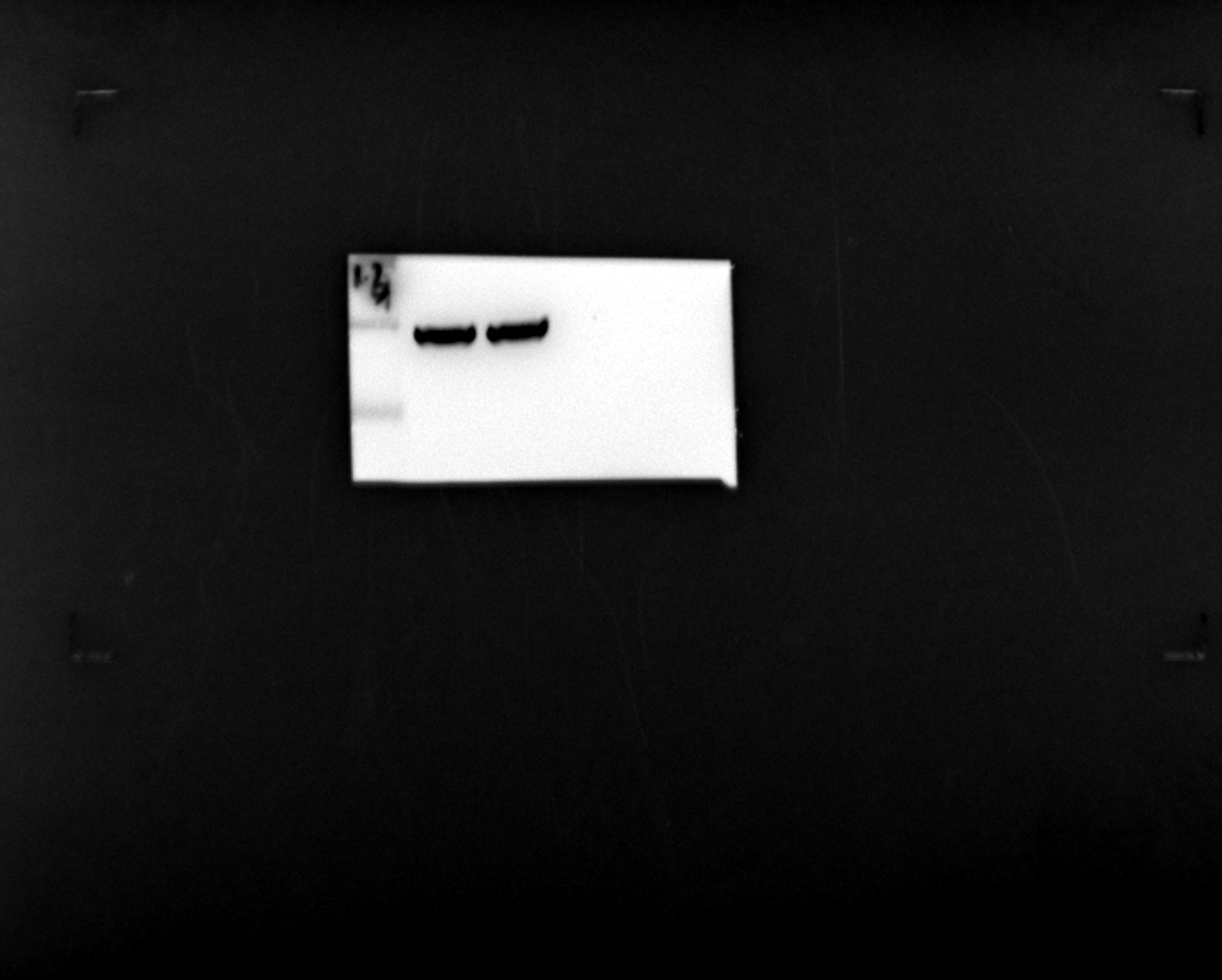

Supplement: Supplementary file 1 — Original western blots [file 41420_2025_2426_MOESM1_ESM.zip › fig4/b/TUBULIN'.Tif]

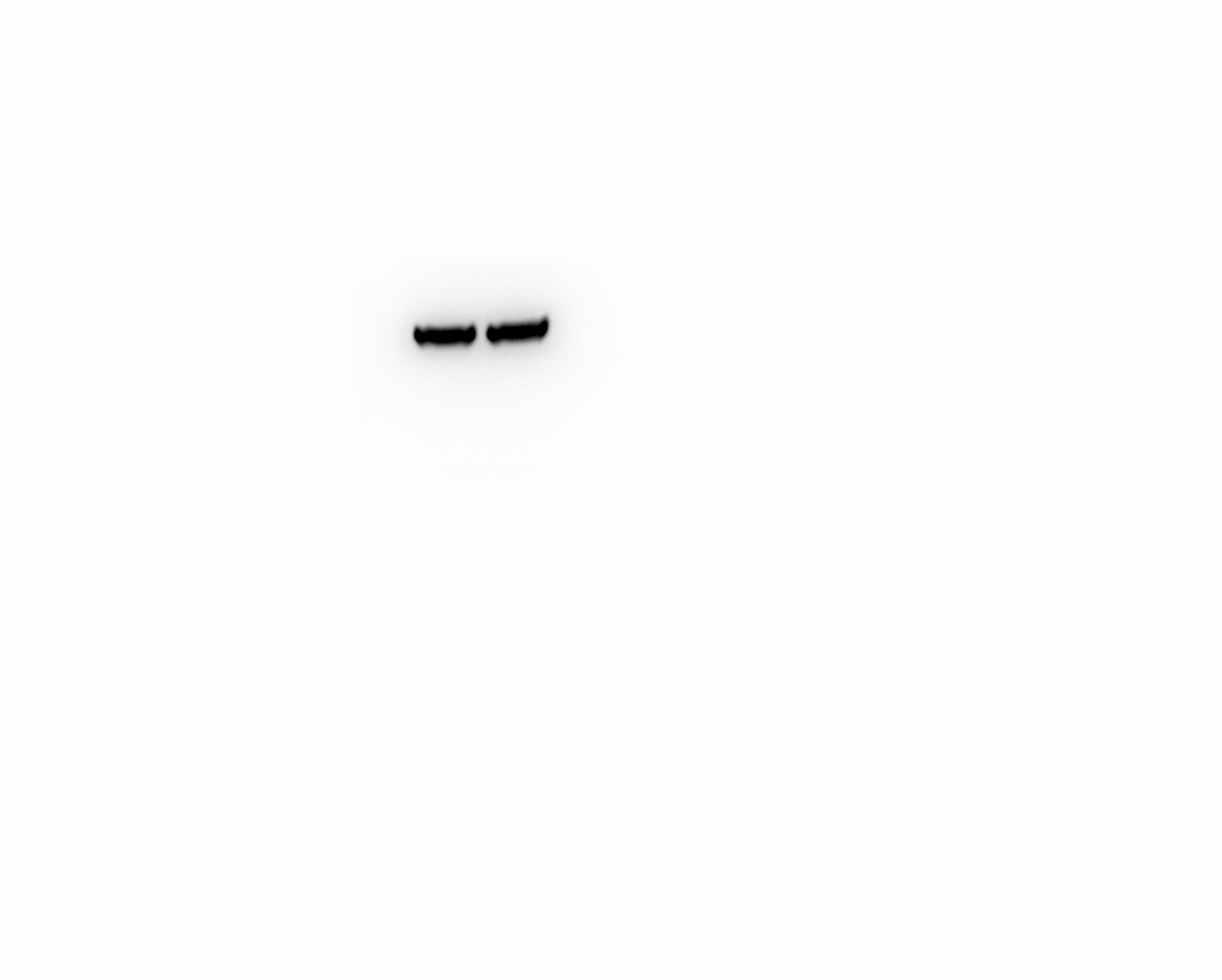

Supplement: Supplementary file 1 — Original western blots [file 41420_2025_2426_MOESM1_ESM.zip › fig4/b/TUBULIN.Tif]
